# Nutritional Data on Selected Food Products Consumed in Oman: An Update of the Food Composition Table and Use for Future Food Consumption Surveys

**DOI:** 10.3390/foods13050787

**Published:** 2024-03-03

**Authors:** Salima Almaamari, Ayoub Al-Jawaldeh, Ibtisam Al Ghammari, Saleh Al Shammakhi, Jokha Al Aamri, Jalila El Ati

**Affiliations:** 1 Nutrition Department, Ministry of Health, Muscat 393, Oman; dr.salima.almamary@gmail.com (S.A.); umwadhah@yahoo.com (I.A.G.); saleh9959@gmail.com (S.A.S.); alaamry99@hotmail.com (J.A.A.); 2 Regional Office for the Eastern Mediterranean, World Health Organization, Cairo 7608, Egypt; aljawaldeha@who.int; 3 SURVEN (Nutrition Surveillance and Epidemiology in Tunisia) Research Laboratory, INNTA (National Institute of Nutrition and Food Technology), 11 Rue Jebel Lakhdar, Bab Saadoun, Tunis 1007, Tunisia; 4 Natural Sciences Department, Faculty of Sciences of Tunis, Campus El Manar, University Tunis El Manar, Tunis 1068, Tunisia

**Keywords:** laboratory values, nutrition label, ultra-processed products, NOVA system, nutrient profile, FoodEx2 system, traffic light label

## Abstract

Food composition data in the Eastern Mediterranean Region countries are often lacking, obsolete, or unreliable. The study aims to provide reliable nutrient data on food products consumed in Oman in order to evaluate their nutritional quality, the consistency of the nutrition labeling and claims, and, ultimately, the use for food consumption surveys and update the current food composition database. Contents of fat, fatty acids, carbohydrates, protein, sugars, and sodium were chemically analyzed in 221 foods and beverages. Products were classified according to their nutritional composition and the extent of processing and coded according to the FoodEx2 system. Labels and laboratory values were compared using the tolerance levels of the European Union. Results indicate that the nutrition labeling aligns with the values obtained in the laboratory, with the exception of 6.3% discrepancies in TFA content, where the reported values are higher than the appropriate reference values. The most frequent category (71.5%) was ultra-processed foods. In terms of inconsistencies in the nutritional claims, 5.1% of food products with claims did not comply with the statement “sugar-free” or “low salt”. Our study provides evidence to support the necessity of comprehensive recommendations for consumers and food industries, which are aimed at enhancing the nutritional quality of products and augmenting consumer awareness.

## 1. Introduction

Food composition tables (FCT) are used for various sectors, including research, education, health, trade, agriculture, industry, and retailing, and are, therefore, useful in manifold ways, such as in nutritional surveillance, food consumption surveys, nutrition labeling, deciding a diet and disease evaluation challenges, developing school menus or standardizing meal planning, setting dietary guidelines, and even assessing the environmental impact of foods [1,2,3,4,5,6,7].

FCT provides information relating to the nutrient composition of foods, with specific reference to energy, major components (water, protein, fat, carbohydrate, alcohol, and ash), inorganic constituents, vitamins, and other components (flavonoids, phytoestrogens phytonutrients, additives, pesticides, and other residues). The nutrient values are either based on chemical analyses performed in analytical laboratories or calculated from the nutrient contents of recipe ingredients using retention and/or yield factors [8]. They are also borrowed values from other tables and databases or presumed values [8].

The Eastern Mediterranean Region (EMR), comprising Afghanistan, Bahrain, Djibouti, Egypt, Iran (Islamic Republic of), Iraq, Jordan, Kuwait, Lebanon, Libya, Morocco, Oman, Pakistan, Palestine, Qatar, Saudi Arabia, Somalia, Sudan, Syrian Arab Republic, Tunisia, United Arab Emirates, and Yemen, is facing the challenge of reducing the growing burden of diet-related non-communicable diseases (NCDs), including type 2 diabetes mellitus, cardiovascular diseases (CVDs), chronic respiratory disease, and some types of cancer [9]. An unhealthy diet is a major risk factor for raised blood pressure, being overweight and obese, raised blood glucose, and raised lipids, which are metabolic and physiological risk factors of NCDs. Increased salt consumption is associated with hypertension and CVDs, and there is evidence that a lower sodium consumption can have a beneficial effect on these morbidities [10,11,12,13,14]. Saturated fatty acids (SFAs) are associated with increased serum levels of low-density lipoprotein, CVDs, and incidences of cancer [15,16]. Trans fatty acids (TFAs) are recognized as harmful nutrients associated with an increased risk of CVDs and mortality [17,18].

In the vast majority of countries of the EMR, the average intake of salt is almost double the WHO’s recommended levels (i.e., less than 5 g per day); sugar intake is also well above WHO’s recommendations (free sugars less than 10% of total energy intake). Moreover, intake of total fat (TF) has steadily increased over the last 50 years, TFA intake is over 1%, and SFA intakes are above the recommended upper limit (10% of total daily energy) [19,20].

Reducing sugar, fat, and salt consumption in line with WHO’s best-buy recommendations is a feasible and meaningful health solution to prevent and control NCDs. The implementation of WHO’s recommendation to reduce salt, sugar, TF, TFA, and SFA consumption has led to a greater focus on reliable nutrient data, hence the need for databases to be reviewed regularly. Data on the composition of foods in the EMR countries are often lacking, obsolete, or unreliable. Some countries do not have food composition data [21,22].

Our study was conducted in the Sultanate of Oman, an EMR country with a land area of 309,500 km^2^ and a population of 5.032 million in 2023, which has recently undergone a rapid increase in the burden of NCDs, with a significant social and economic impact in terms of health care, loss of productivity, and premature mortality [23]. A recent study reported that two-thirds of Omani adults are overweight or obese, one-third is obese, one-third has high blood pressure or is currently under medication, and more than 15% have a diagnosis of raised blood glucose or are on diabetes medication and/or are diagnosed with diabetes [24]. Unhealthy diet behaviors of Omani adults underline the predominance of the burden of NCDs in the country, e.g., 61% did not meet the recommended number of five servings/day of fruits and/or vegetables; 76% often/always added salt to food before or when eating; 25% reported eating processed food high in salt. The most prevalent risk factor is primarily related to high salt, sugar, and fat intake [24].

In the Sultanate of Oman, food composition databases are incomplete, outdated, and unreliable [25,26,27]. Consequently, primary users of FCT in Oman, including nutritionists, epidemiologists, dieticians, and food producers, frequently resort to utilizing international FCTs or accessible databases from neighboring EMR countries, such as Iraq or Iran. 

In this context, our study aimed to (i) classify the selected food products based on their degree of processing and nutritional composition; (ii) compare labels with corresponding laboratory values; (iii) develop a Front-of-Pack nutrition labeling system as a critical tool for informing consumers about the nutritional content of purchased items; (iv) assess the contribution of these products to reference intake; (v) monitor the compliance of nutrition claims, when available; (vi) ultimately, serve as the cornerstone for the development of a comprehensive FCT specifically tailored for the country and for utilization in future food consumption surveys.

## 2. Materials and Methods

The design, sampling and laboratory analyses were performed in 2022.

**Figure 1:**
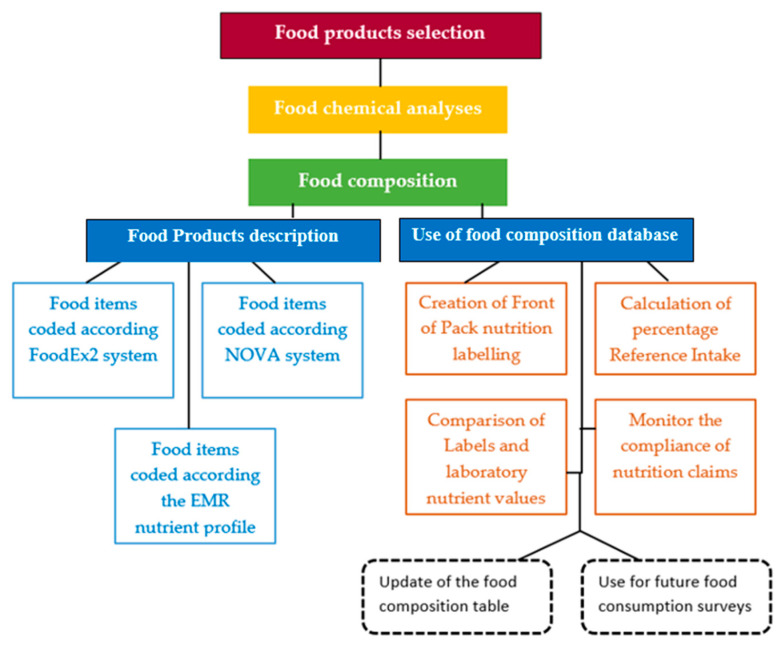
Flow chart of the methods used. The dotted line indicates prospective utilization of the food composition database produced through this investigation.

The following flow chart (Figure 1) presents the design of the methods used to analyze the food composition data provided by laboratory analysis of the selected food products.

### 2.1. Identification of Food Products

In order to obtain a list of the most consumed food products, four of the big hypermarkets from the Governorate of Muscat (Oman’s capital city) were contacted. Muscat governorate markets were chosen due to the presence of many citizens from different governorates of the Sultanate and the compilation of the most common options in foodstuffs. One of those hypermarkets responded to us and sent us the list of all foods and drinks sold in the market, as this type of information is confidential for most markets. The list was sorted according to their sales rate, and the most consumed products were selected. In each food item from the top five, we chose the Omani brands to facilitate the reformulation’s interventions in addition to the other brands in the top five list. A final list of 221 food items was selected from the most consumed products and purchased from the markets and has been prepared to start analyzing in the laboratory. For every food item, we performed the analysis one time with Internal Quality Control sample (know value sample), and our method is based on AOAC method. In case any sample does not meet the value, then we only perform repeated analysis.

### 2.2. Analytical Parameters

Chemical analyses to assess the nutrient contents of the selected food products were carried out in the United Integrated Laboratories, located in Barka near the capital Muscat. For every food item, the contents of total fat (g), SFAs (g), polyunsaturated fatty acids (PUFAs) (g), monounsaturated fatty acids (MUFAs) (g), TFAs (g), carbohydrates (g), total nitrogen (g), total sugars (g), glucose (g), sucrose (g), maltose (g), lactose (g), and sodium (g) were analyzed. Official methods of analysis of Association of Official Analytical Chemist (AOAC) were used to analyze nutrients in foods [28,29,30,31,32] (Table 1).

The energy content of each food item “as sold” was calculated according to the following formula [8]:

Energy kcal (kJ) = [carbohydrates *×* 4 kcal/g (17 kJ/g) + total fat *×* 9 kcal/g (37 kJ/g) + protein *×* 4 kcal/g (17 kJ/g) + fiber *×* 2 kcal/g (4 kJ/g)

### 2.3. Food Products Description

#### 2.3.1. Food Items Coded According to FoodEx2 System

In order to harmonize our food dataset and allow comparison across groups and countries, we used FoodEx2, a standardized system of Global Dietary Database and WHO/FAO GIFT, for classifying and describing food data [33,34,35]. FoodEx2 consists of a core list of food items that represent the minimum level of detail needed for food assessments, and facets provide further detail to the information of the food list term. The nature of the food itself is linked to level of processing: raw primary commodities (RPC), RPC derivatives, and composite foods. RPCs are unprocessed single-component foods or whose nature has not been changed by processing. RPC derivatives are single-component foods that have been physically changed by processing. Composites are foods consisting of multiple components [36].

#### 2.3.2. Food Items Classified According to NOVA System

The NOVA Classification, which was developed in 2010 by Monteiro (Brazil) and is popularized around the world, does not take into account nutritional values but rather the extent of processing of the foods. It distinguishes four food groups according to the extent of their processing [37]. 

#### 2.3.3. Food Items Classified According to the EMR Nutrient Profile

Data provided from chemical laboratory analyses were used to classify food products according to the nutrient profile model developed by the WHO for the EMR [38]. It consists of classifying foods according to their nutritional composition in order to allow differentiation between foods that can form part of a healthy diet and those that are less healthy. 

The model consists of 18 food categories or groups for which thresholds have been established in relation to energy, total fats, saturated fats, total sugars, added sugars, non-sugar sweeteners, and salt. The thresholds not to be exceeded are based on the dietary objectives recommended by the WHO for the prevention of obesity and related non-communicable diseases, as well as on the recommendations concerning sugars and salt [39,40]. If one of the thresholds is exceeded, no marketing action aimed at children should be permitted. This model was used to classify our food items into three groups according to the permission or not of their advertisement: “permitted”, “permitted subject to certain conditions”, and “not permitted”.

### 2.4. Different Uses of Food Composition Data

Three main uses of food composition table are presented in detail throughout the following subsections.

#### 2.4.1. Creation of Front of Pack (FoP) Nutrition Labeling

The FoP color-coded nutrient-based schemes were developed in accordance with the guidance of UK Food Standards Agency [41] and contains

Format “energy + 4”: information on the energy value in kcal and kJ, plus the amounts of total fat, saturates, sugars, and salt in grams per 100 g/mL and per portion of the product “as sold”.

Descriptors “High”, “Medium” or “Low” together with the colors red, amber, or green, respectively, to reinforce their meaning. Criteria from Regulation (EC) No 1924/2006 [42,43] for red (HIGH), amber (MEDIUM), and green (LOW) were used (Table 2).

#### 2.4.2. Calculation of Percentage Reference Intake

Percentage reference intake (% RI) given per 100 g/mL of the product “as sold” using ‘Reference intake’ of an average adult (8400 kJ/2000 kcal) (Table 3). The daily reference intake (RI) for FoP nutrition labels is set by the European Commission and Member States [41]. RIs for fat, saturated fatty acids, sugars, and salt are the maximum amounts that should be consumed per day. 

The food item is considered a high source of the considered nutrient if % RI is >20%, good source when % RI is between 11 and 20%, medium if % RI is between 5 and 10%, and low if % RI is <5% [44].

#### 2.4.3. Comparison of Label and Laboratory Nutrient Values

Available food labeling values on proteins, carbohydrates, total fat, SFAs, sugars, and sodium were compared to the corresponding values obtained by laboratory analyses. Because of the lack of this type of legislation in Sultanate of Oman, nutrition labeling compliance was tested using the tolerance thresholds set by Regulation (EU) No 1169/2011 of the European Parliament, including the uncertainty of measurement associated with a measured value [45], and Regulation (EU) 2019/649 of 24 April 2019, established for TFAs other than TFAs naturally occurring in fat of animal origin [46] (Table 4). For every food item, food labeling value is considered compliant with the analyzed value if this later value is within the lower and higher tolerance of the declared value, which is calculated using the following tolerance thresholds.

Among the analyzed food products, 32.6% (*n* = 74) presented nutritional information with claims. Only compliance of claims on energy, protein, sugar, and salt was tested using content thresholds set by Regulation (EC) No. 1924/2006 for nutritional or health claims [42] (Table 5). For food products (with added vitamins and minerals claims), no laboratory data were available to compare or control the label values.

### 2.5. Statistical Analyses of Data

Management, check, and calculation of the derived variables (energy), and creation of new classifications of the data files were performed using the Stata software (version 14.0; StataCorp, College Station, USA) [47]. 

## 3. Results

### 3.1. Food Product Description

Food products were categorized into eight aggregated groups from the nutrient profile food groups. Out of the total of 221 food products, 25.3% were sweet snacks, cakes, biscuits, chocolate, and sugar confectionery, 20.4% were dairy products, 19.5% were beverages, 12.2% were processed fruit, vegetables, and legumes, 10.0% were sauces and dressings, 8.6% was processed meat, poultry, and fish, 3.2% was bread and cereal products, and 0.9% was natural food (Table 6).

Our analysis found that only 66.5% of these products displayed clear food information to consumers and 55.5% mandatory nutritional information (energy in both kJ and kcal, fat, saturates, carbohydrates, sugars, protein, and salt) [39]. Among these food products, 42.1% had claims on the package (Table 6).

According to the FoodEx2 system classification, 52.0% of the food item products analyzed by our laboratory were composites, 46.6% were raw primary commodity derivatives, and only 1.4% were raw primary commodities (Supplementary Table S1).

The distribution of food products classed according to the NOVA system is detailed in Supplementary Table S2. According to the extent of industrial processing, ultra-processed and processed food products were the most frequent, with an overall average frequency of 88.2% (71.5% and 16.7%, respectively), while unprocessed or minimally processed foods represented 10.9%, and processed culinary ingredients only 0.9%. 

Overall, of the 221 analyzed food products, only 12.2% were exempt from marketing restrictions and considered to be part of a healthy diet according to the nutrient profile model used in the EMR region. For the other items of products, 35.3% were classified as the “not permitted marketing” category (the whole category is classified as not permitted and does not need to check the levels for salt, sugar, SFAs, and total fat). Out of the remaining 52.5% of products, 19.0% were not permitted because of high salt; 10.4% were not permitted because of high salt and total fat; 9.5% were not permitted because of excess sugar; 5.0% were not permitted because of high total fat; 5.0% were not permitted because of excess of salt, total fat, and SFAs; 1.8% were not permitted because of excess salt and sugar; 0.9% were not permitted because of total fat and sugar; 0.5% were not permitted because of total fat and TFAs; 0.4% were not permitted because of SFAs and salt. Results are detailed in Supplementary Table S3. 

### 3.2. Energy and Nutrient Composition of Food Products

The energy and nutrient contents of each food item product assessed by laboratory analyses are detailed in Supplementary Table S4. Table 7 presents the energy, macronutrient, and sodium values in food products classified into eight groups.

On average, these food products contain protein between 0 (Group 1) and 18.3 g (Group 5), total fats between 0 (Group 1) and 22.2 g (Group 2), and carbohydrates between 5 (Group 4) and 82.8 g (Group 1). The highest average SFA content was found in the dairy products group (9.7 g), with a maximum content of 27.8 g. The highest TFA content (4.1 g) was found in (Group 2). The lowest and highest total sugar content was found in Group 1 (80.7 g) and Group 5 (0.5 g). Sucrose content ranged between 0.1 g, reported in both Group 1 and Group 5, and 15.1 g was found in Group 2. Maltose and lactose content did not vary much between the analyzed food items. Sauces and dressings were the highest in sodium content.

### 3.3. Food Products Categorized According to the Front of Pack Nutrition Labeling

The traffic-lights label system based on the levels of total fat, SFAs, sugars, and salt content showed that only 2.7% of food products were considered healthy, as all stated nutrient levels that were low. Out of the remaining items, 32.2% showed medium or high levels of one of the stated nutrients, 13.6% contained medium or high levels of two of the stated nutrients, 33.0% contained medium or high levels of three of the stated nutrients, and 18.5% contained medium or high levels of all of the stated nutrients. Details of the results regarding the FoP categorization are reported in Supplementary Table S5.

### 3.4. Daily Reference Intake Scores Provided by Food Products

For nutrients to be limited, high sources (equal to or above 20% of daily reference intake) were found in 35.8% of the total food products for total fat, 58.8% for SFAs, 21.3% for sugars, and 28.5% for salt, while low sources were found in 52.9%, 43.4%, 47.1%, and 35.3%, respectively (Table 8). 

For energy, 22.2% of the food products were high sources, and 39.8% were low sources. Only 17.2% of the food products were high sources of protein, and 23.5% were for carbohydrates. More details regarding the percentage daily reference intake and scores are reported in Supplementary Table S6.

### 3.5. Accurateness of the Nutritional Labels

In the context of food labeling, all reported nutrient values, specifically protein (95.5% of all food products), carbohydrates (96.0%), total fat (96.0%), saturated fatty acids (84.6%), sugars (86.0%), and sodium (90.0%), were found to be consistent with the analyzed samples in the laboratory. Concerning the laboratory values for TFAs, 6.3% of the total food products presented values exceeding the acceptable threshold of 2% of total fat, which falls within the range of 2.7 to 61.7 g/100 g of total fat. Details regarding the results of lower and higher tolerance of every item are reported in Supplementary Table S7.

### 3.6. Accurateness of the Nutrition Claims

Two types of food products with nutritional claims of “sugar-free” and “low salt” did not comply with the regulation, while the other evaluated food items were compliant (Table 9).

## 4. Discussion

This study provides the first comprehensive comparison of laboratory and label values for proteins, carbohydrates, sugars, SFAs, TFAs, and sodium in major agri-food products that contribute to food consumption in Oman. The findings showed that among the various food products analyzed in this research and categorized using the NOVA classification, the category with the highest frequency (71.5%) consisted of UPFs. This classification was corroborated by the FoodEx2 system, which identified nearly all UPFs (99.4%) as composite foods or RPC derivatives, indicating that these foods had undergone significant processing transformations. This result shows that the FoodEx2 system perfectly analyzes the extent of food processing.

When examining the distribution of UPFs based on the number of red FoP traffic lights, it was observed that 36.1% of items displayed one red traffic light, 40.5% had two, 8.9% had three, and none received all four red traffic lights simultaneously (indicating high levels of fat, SFAs, sugars, and salt). Furthermore, UPFs were found to be prevalent within the green FoP traffic-light labeling category, with figures ranging from 39.9% in products low in fat to 38.6% in products low in saturated fatty acids (SFAs), through to 39.9% in products containing sugar, and 37.3% in products low in salt. Additionally, 14.6% of UPFs displayed no red FoP traffic-light labeling. These included items such as flavored milk, custards, industrial juices, and malt beverages. 

It is well-established that UPFs tend to be high in energy, fats, sugar, and sodium while lacking in dietary fiber and essential micronutrients [48]. Hence, it was expected that the frequency of UPFs would be reduced in the category signifying the highest nutritional quality, indicated either by the color green or the absence of red traffic lights. Our results align with those of the UK National Diet and Nutrition Survey [49], which discovered that not all UPFs exhibited an unhealthy nutrient profile, with more than half of them lacking any red FoP traffic lights. These findings imply that FoP traffic-light labeling, serving as a nutrient profiling system, is capable of distinguishing the nutritional quality of food and beverages to some extent, but it only partially captures the extent and purpose of food processing. In this research, we observed that two food items, both labeled with the same color on Front-of-Pack (FoP) traffic-light labels, actually belong to different NOVA classifications. As an example, cream caramel received green and amber FoP traffic-light labels, but it falls under NOVA 4 (UPFs), whereas Laban up also received green and amber FoP traffic-light labels but is classified under NOVA 1. 

Typically, products labeled with a green FoP traffic light are perceived as healthier, which can lead to greater intentions to purchase. Hence, in the absence of information pertaining to other facets of food, consumers may primarily rely on the nutritional quality of food when making food choices, irrespective of other individual factors. On the other hand, food producers tend to reformulate their products to achieve more favorable FoP labeling by decreasing the sugar, fat, and salt content or augmenting the fiber content to attract consumers, regardless of the degree and scope of processing necessitated for such a transformation.

UPFs are characterized as ready-to-eat products primarily comprised of limited or no whole foods, typically demonstrating low nutritional qualities [50,51]. This poses a substantial concern, particularly in light of the increasing worldwide consumption of UPFs. Previous research has shown an association between higher UPF consumption and adverse health outcomes such as obesity, hypertension, breast cancer, diminished life expectancy, and potential risk on both maternal and neonatal health [52,53,54,55]. Some studies have proposed that exposure to endocrine-disrupting chemicals may represent a potential mechanism linking UPF intake to adverse health outcomes [53,56,57]. In addition, an alternative meta-analysis involving 40 prospective cohort studies assessed the relationship between UPFs and overall mortality. The findings showed a positive correlation between increased intake of sugar-sweetened beverages, artificially sweetened beverages, processed meat, and red meat and all-cause mortality. Conversely, the consumption of breakfast cereals demonstrated an inverse association with all-cause mortality [58].

Nonetheless, in a recent investigation [59], the researchers examined the reliability and efficacy of the NOVA classification system, aiming to ascertain whether this system yields consistent food categorizations among users and its capacity to inform public health policies or furnish valuable insights for consumers. Their findings suggested that the current NOVA criteria do not enable foods to be definitively categorized as ultra-processed, highlighting the need for enhancements in the NOVA classification system to improve the consistency of food assignments.

In the present study, when considering both ultra-processing and the nutritional quality of foods, more than one-quarter of the analyzed food products were classified with amber and red FoP traffic-light labeling. Processing and nutritional quality are two dimensions of food that may independently or together influence the risk of chronic diseases. Some studies have investigated their individual and combined contributions to overall dietary quality. In this sense, a cross-sectional observational study provides evidence that food nutritional quality and processing are not mutually exclusive and should be considered as underpinning the dimensions of the diet [60]. A prospective cohort study found that adults with poor diets and a higher intake of UPFs faced the greatest risk of all-cause and cardiovascular disease mortality [61]. A review of prospective cohort studies demonstrated that the association between UPF consumption and obesity and health-related outcomes remained consistent, even after accounting for dietary quality or patterns [62].

The findings indicate that within the sampled food products, 21.3% exhibited high sugar content, 35.8% had high fat content, 58.8% were rich in saturated fatty acids (SFAs), and 28.5% contained elevated levels of salt. Consequently, consumers are advised to restrict their consumption of these food items and beverages, consuming them less frequently and in moderated quantities.

The results of this study indicate that the nutrient values stated on a label generally align with the values obtained in the laboratory when using the tolerance levels of the European Union, with the exception of a 6.3% discrepancy in TFA content, where the reported values are higher than the appropriate reference values. In terms of inconsistencies in the nutrition claims, 5.1% of food products with claims did not comply with the statements “sugar-free” or “low salt”. These findings underscore the significance of the vigilant monitoring of nutritional labels as a means to implement measures that promote the well-being of consumers.

The limitation of this study lies in the potential bias introduced during the selection of food products, given that a majority of the chosen items were processed since UPFs are accessible in the Oman market. All inquiries were thoroughly deliberated and resolved through unanimous consensus among all the authors.

## 5. Conclusions

The results showed consistency between the NOVA classification and the FoodEx2 system in terms of food processing, but the labeling captures only the nutritional quality of the food and beverages and not the extent of food processing, which is only complementary. In addition, non-compliance with nutritional claims and nutrition information was identified. These findings emphasize the importance of implementing strategies to address the regulation of UPFs and improve labeling and information for consumers. Government strategies should set up, on the one hand, guidelines and regulations to limit the production and marketing of UPFs, especially targeting their negative health effects, and on the other hand, provide clear and informative labeling that includes not only the nutritional content but also any potential health risks associated with UPFs.

Our study provides evidence to support the necessity of comprehensive recommendations directed towards both consumers and food processing industries aimed at enhancing the nutritional quality of products and augmenting consumer awareness. To facilitate informed decision-making regarding food choices, consumers should prioritize minimally processed and whole foods, carefully scrutinize nutritional labels when purchasing packaged foods, opt for products with reduced levels of added sugar, salt, and fat that are rich in essential vitamins, minerals, and fibers, and exercise portion control to mitigate calorie overconsumption. Conversely, food processing industries are urged to improve the nutritional composition of their products by reformulating them to lower the quantities of added sugars, saturated fats, and sodium while simultaneously elevating the levels of beneficial nutrients, such as fibers, vitamins, and minerals. This can be achieved through the integration of healthier ingredients and innovative technologies that uphold optimal nutrition profiles without compromising taste or texture. Furthermore, these industries should prioritize the provision of clear and transparent labeling, facilitate the accurate communication of nutritional information to consumers, adopt responsible marketing practices, and refrain from misleading claims. By adhering to these strategies, consumers can make enlightened choices and harbor confidence in the foods they incorporate into their diets, thereby fostering increased overall well-being.

Our findings underscore the importance of diligent monitoring of nutritional labels as a catalyst for driving enhancements in the nutritional quality of food products and contributing to improved dietary patterns and better health of customers.

## Figures and Tables

**Table 1:** Analytical methods of nutrient in food products [28,29,30,31,32].

Test Parameters	UIL Method of Test	Reference Method
Total Fat (TF)	UIL-SOP-TECH-033	AOCS Official Method Ce 2–66, 2017 [28].
Saturated Fatty Acids (SFAs)	UIL-SOP-TECH-033	
Poly Unsaturated Fatty Acids (PUFAs)	UIL-SOP-TECH-033	
Monounsaturated Fatty Acids (MUFAs)	UIL-SOP-TECH-033	
Trans Fatty Acids (TFAs)	UIL-SOP-TECH-033	
Sodium (Na) ^1^	UIL-SOP-TECH-008	Official methods of analysis of AOAC international, 2008 [29].
Total Sugar	UIL-SOP-TECH-023	Official methods of analysis of AOAC international, 2007 [30].
Glucose	UIL-SOP-TECH-023	
Fructose	UIL-SOP-TECH-023	
Sucrose	UIL-SOP-TECH-023	
Maltose	UIL-SOP-TECH-023	
Total Nitrogen ^2^	UIL-SOP-TECH-014	Official methods of analysis of AOAC international, 2008 [31].
Carbohydrates	UIL-SOP-TECH-016	Official methods of analysis of AOAC international, 2002 [32].

^1^. Salt values are calculated from total sodium (Na) value multiplied by sodium conversion factor: Salt = Na × 2.5 [8]. ^2^. Total protein values are derived from the total nitrogen (N) value multiplied by the nitrogen conversion factor: Total protein = N × 6.25 [8].

**Table 2:** Criteria for 100 g/mL of foods and drinks (per 100 mL) [37].

Food Whether or Not It Is Sold by Volume					Drinks			
Text	Low	Medium	High		Low	Medium	High	
Color code	Green	Amber	Red		Green	Amber	Red	
			>25% of RIs	>30% of RIs			>25% of RIs	>30% of RIs
Total Fat	≤3.0 g/100 g	>3.0 to ≤17.5 g/100 g	>17.5 g/100 g	>21 g/portion	≤1.5 g/100 mL	>1.5 to ≤8.75 g/100 mL	>8.75 g/100 mL	>10.5 g/portion
SFAs	≤1.5 g/100 g	>1.5 to ≤5.0 g/100 g	>5.0 g/100 g	>6.0 g/portion	≤0.75 g/100 mL	>0.75 to ≤2.5 g/100 mL	>2.5 g/100 mL	>3.0 g/portion
Sugars	≤5.0 g/100 g	>5.0 to ≤22.5 g/100 g	>22.5 g/100 g	>27 g/portion	≤2.5 g/100 mL	>2.5 to ≤11.25 g/100 mL	>11.25 g/100 mL	>13.5 g/portion
Salt	≤0.3 g/100 g	>0.3 to ≤1.5 g/100 g	>1.5 g/100 g	>1.8 g/portion	≤0.3 g/100 mL	>0.3 to ≤0.75 g/100 mL	>0.75 g/100 mL	>0.9 g/portion

**Table 3:** Reference intake (RI) ^1^ for FoP nutrition labels [41].

Nutrient	Value ^2^	Nutrient	Value ^2^
Energy	8400 KJ ^3^	Carbohydrates	260 g
Energy	2000 kcal ^3^	Sugars	90 g
Total Fat	70 g	Protein	50 g
Saturated fatty acids	20 g	Salt	6 g

^1^: the calculation of the % RI used the formula: % RI = (Amount of [nutrient] per 100 g/mL/RI × 100. ^2^: these are ‘adult’ values, based on an average-sized woman performing an average amount of physical activity [41]. ^3^: when the % RI is expressed for energy per 100 g/mL, the “Reference intake of an average adult (8400 kJ/2000 kcal)” must accompany the declaration [41].

**Table 4:** Tolerances for food products other than food supplements [45,46].

Nutrient	Tolerances for Food Products	
Protein, carbohydrates, sugars	<10 g/100 g	±2 g
	10–40 g/100 g	±20%
	>40 g/100 g	±8 g
Total Fat	<10 g/100 g	±1.5 g
	10–40 g/100 g	±20%
	>40 g/100 g	±8 g
Saturated fatty acids	<4 g/100 g	±0.8 g
	≥4 g/100 g	±20%
Sodium	<0.5 g/100 g	±0.15 g
	≥0.5 g/100 g	±20%
Trans Fatty Acids	Not exceed 2 g per 100 g of fat	

Example: A food product with a nutrition declaration of sugars of 8.5 g/100 g and no claim made about its sugar content. The range of tolerance is 6.5 to 11 g/100 g according to the criteria laid down in Table 4. The declared value (8.5) is within the range of 6.5 to 11 g/100.

**Table 5:** Tolerances for food products with nutrition claims [42].

Nutrient	Conditions Applying to Nutrition Claims
Low energy	The product does not contain more than 40 kcal (170 kJ)/100 g for solids or more than 20 kcal (80 kJ)/100 mL for liquids.
Sugar-free	The product contains no more than 0.5 g of sugar per 100 g or 100 mL
Source of protein	At least 12% of the energy value of the food is provided by protein
High protein	At least 20% of the energy value of the food is provided by protein
Low salt	the product contains no more than 0.12 g of sodium, or the equivalent value for salt, per 100 g or per 100 mL

**Table 6:** Proportion (%) of nutrition components found on the product label by category of food.

Food Groups	Total Products		Label Clear to Read (%)	Food Packaging Claim (%)	Mandatory Nutritional Information ^1^ (%)
	*n*	%			
Group 1: Natural food	2	0.9	100.0	0.0	0.0
Group 2: cakes, biscuits, chocolate, sugar confectionary	56	25.3	46.4	37.5	76.4
Group 3: Bread and cereal products	7	3.2	14.3	100.0	85.7
Group 4: Dairy products	45	20.4	86.7	53.3	71.1
Group 5: Processed meat, poultry and fish	19	8.6	52.6	63.2	57.9
Group 6: Processed fruit, vegetables and legumes	27	12.2	70.4	18.5	25.9
Group 7: Sauces and dressing	22	10.0	63.6	22.7	54.6
Group 8: Beverages	43	19.5	83.7	44.2	27.9
Total	221	100.0	66.5	42.1	55.5

^1^: energy, fat, saturates, carbohydrates, sugars, protein, and salt.

**Table 7:** Energy and nutrient values for 100 g edible portion of food products distributed into categories.

	Natural Food (*n* = 2)	Sweet Snacks, Cakes, Biscuits, Chocolate and Sugar Confectionary (*n* = 56)	Bread and Cereal Products (*n* = 7)	Dairy Products (*n* = 45)	Processed Meat, Poultry and Fish (*n* = 19)	Processed Fruit, Vegetables and Legumes (*n* = 27)	Sauces and Dressing (*n* = 22)	Beverages (*n* = 43)
Energy (kJ)	1407.4 ^1^	1933.9	1744.8	745.8	761.0	488.2	592.4	461.4
	1393.0–1421.9 ^2^	486.2–2669.3	1624.5–1974.2	121.0–1468.5	450.5–1116.6	212.1–1667.9	11.6–2992.9	55.3–2201.8
Energy (kcal)	331.2	461.6	412.7	179.8	181.8	115.4	142.3	109.2
	327.8–334.6	115.1–643.3	383.0–470.7	28.7–353.5	106.0–268.8	50.1–392.7	2.72–727.5	13.0–526.8
Protein (g)	0	7.3	10.3	6.8	18.3	4.3	0.8	2.1
	0	0.0–26.0	7.2–18.3	1.7–24.3	11.7–25.6	0.4–7.6	0.0–5.2	0.0–24.9
Total Fat (g)	0	22.2	7.2	14.8	9.4	1.7	9.7	2.2
	0	0.0–51.9	2.0–20.9	0.6–34.1	0.0–20.6	0.0–20.8	0.0–79.2	0.0–29.6
*Saturated Fatty Acids (g)*	0	9.5	3.3	9.7	4.0	0.4	2.2	1.4
	0	0.0–26.8	0.4–11.4	0.4–27.8	0.0–10.3	0.0–2.3	0.0–13.6	0.0–20.2
*Polyunsaturated Fatty Acids (g)*	0	4.0	1.3	1.4	2.1	0	0	0
	0	0.0–18.6	0.5–2.7	0.0–7.3	0.0–5.9	0	0	0.0–0.7
*Monounsaturated Fatty Acids (g)*	0	8.8	2.6	3.3	3.4	0.7	2.4	0.5
	0	0.0–30.1	0.1–6.9	0.1–11.4	0.0–8.2	0.0–11.7	0.0–22.0	0.0–6.5
*Trans Fatty Acids (g)*	0	0.1	0	0.2	0	0	0	0
	0	0.0–4.1	0	0.0–1.8	0.0–0.2	0	0	0.0–0.7
Carbohydrates (g)	82.8	58.2	76.6	5.0	6.0	20.8	12.8	20.3
	81.9–83.6	13.7–125.3	62.1–84.2	0.0–15.4	1.0–20.9	9.4–88.3	0.4–41.6	3.0–96.9
*Total Sugar (g)*	80.7	20.4	18.9	2.7	0.5	6.3	9.5	18.7
	80.3–81	0.0–81.5	8.2–27.7	0.0–15.3	0.0–4.0	0.1–63.8	0.0–34.1	2.2–96.9
*Glucose (g)*	37.1	3.9	3.1	0.4	0.4	3.0	3.6	3.0
	37.1–37.2	0.0–81.5	0.2–9.8	0.0–1.8	0.0–3.4	0.0–33.0	0.0–17.8	0.0–31.9
*Fructose (g)*	43.4	1.0	2.4	0	0.1	1.2	2.7	3.0
	42.9–43.8	0.0–6.8	0.0–9.9	0.0–0.2	0.0-0.2	0.1–15.9	0.0–15.6	0.0–28.7
*Sucrose (g)*	0.1	15.1	13.7	0.5	0.1	1.7	3.1	9.5
	0.1–0.1	0–52.5	4.0–28.1	0.0–11.4	0.0–0.6	0.1–8.1	0.0–16.3	0.0–87.9
*Maltose (g)*	0.1	0.6	0.2	0	0	0.4	0.8	0.2
	0.1–0.1	0–5.7	0.0–1.1	0.0–0.2	0.0–0.1	0.0–9.0	0.0–7.7	0.0–3.9
*Lactose (g)*	0.1	1.3	0.1	3.2	0.1	0.1	0.4	0.19
	0.1–0.1	0.0–8.1	0.0–0.3	0.0–45.9	0.0–0.8	0.0–0.5	0.0–2.0	0.0–3.0
Sodium (mg)	0	338.6	433.8	540.8	485.0	690.0	3372.7	44.4
	0	0.0–1542.5	0.3–1364.3	0.0–1898.8	211.2–1301.1	0.0–5040	163.5–19,900	0.0–329.7

^1^: Mean. ^2^: min-max.

**Table 8:** Percentage of daily reference intake scores of analyzed food products.

Nutrient	Percentage Daily Reference Intake Provided by Food Products			
	Low Score ^1^ (%)	Medium Score ^2^ (%)	Good Score ^3^ (%)	High Score ^4^ (%)
Energy	39.8	15.4	22.6	22.2
Protein	33.9	23.5	25.3	17.2
Carbohydrates	47.1	21.3	8.1	23.5
*Sugars*	47.1	13.6	18.1	21.3
Total Fat	52.9	3.6	7.7	35.8
*Saturated Fatty Acids*	43.4	6.8	8.6	58.8
Salt (g)	35.3	14.0	22.2	28.5

^1^: Percentage of daily reference intake is <5%. ^2^: Percentage of daily reference intake between 5 and 10%. ^3^: Percentage of daily reference intake between 11 and 20%. ^4^: Percentage of daily reference intake is >20%.

**Table 9:** Compliance of nutrition claims.

Food Category	Claim	Nutrient Content	Comment
Yogurts, sour milk, cream and other similar foods (1 *item*)	Source of protein	*Protein*: 21.0% of energy value	Compliant
Yogurts, sour milk, cream and other similar foods (1 *item*)	High protein	*Protein*: 24.8% of energy value	Compliant
Processed fruit, vegetables and legumes (1 *item*)	Sugar-free	*Sugar*: 0.74 g/100 g of the product	Non-compliant
Sauce and dressing (1 *item*)	Low salt	Salt: 1.5 g/100 g of the product	Non-compliant
Beverage (1 *item*)	Low energy	Energy: 18.5 kcal/100 mL of the product	Compliant
Cheese (2 *item*)	TFAs ^1^ free	TFAs: ≤0.5% of energy	Compliant

^1^: Trans Fatty Acids.

## Data Availability

The original contributions presented in the study are included in the article/Supplementary Material, further inquiries can be directed to the corresponding author.

## Supplementary Materials

The following supporting information can be downloaded at: https://www.mdpi.com/article/10.3390/foods13050787/s1, Table S1: Food description and coding according FoodEx2 system; Table S2: Classification of food products according to the NOVA system; Table S3: Classification of food items according to the EMR nutrient profile; Table S4: Nutrient composition of sampled food products; Table S5: Creation of Front of Pack (FoP) nutrition labeling in units of mass and units per portion; Table S6: Percentage reference intake (% RI) and Scores of IR information; Table S7: Label and laboratory values comparison.
