# Supplementary material for: Nutritional Data on Selected Food Products Consumed in Oman: An Update of the Food Composition Table and Use for Future Food Consumption Surveys
Source: Foods. 2024 Mar 3;13(5):787. doi: 10.3390/foods13050787 (PMC10930989; doi:10.3390/foods13050787)
Supplement: Supplementary file 1 [file foods-13-00787-s001.zip › foods-2889464-supplementary.pdf]

Supplementary Material

Table S1: Food description and coding according FoodEx2 system.

Sample ID (from lab)	Name of Product & Brand	Food Item	FOOD TYPE FE2	FoodEx2 Code	FoodEx2 Description	Description
UIL.21.14412	Pure honey / Capilano	Food	RPC - Raw Primary Commodities	A033J	Honey	Honey is a natural sweet substance produced by bees from the nectar of plants or from secretions of living parts of plants or excretions of plant-sucking insects on plants. Bees collect it, transform it by combining with specific substances of their own, deposit it, dehydrate it, store it and leave it in honeycombs to ripen and mature.
UIL.21.14414	Natural Honey / Alshifa	Food	RPC - Raw Primary Commodities	A033J	Honey	Honey is a natural sweet substance produced by bees from the nectar of plants or from secretions of living parts of plants or excretions of plant-sucking insects on plants. Bees collect it, transform it by combining with specific substances of their own, deposit it, dehydrate it, store it and leave it in honeycombs to ripen and mature.
UIL.21.13443	Wrigleys Doublemint Chewing Gum	Food	Composites	A035M#F04.A00XZ	Chewing gum, INGREDIENT = Mints	The group includes any type of Chewing gum, i.e. a confectionery product with the characteristic of being chewed but not swallowed. Aromatic fresh herb from the plant classified under the genus Mentha L. spp., commonly known as Mints.
UIL.21.14391	Nutella Hazelnut Spread With Cocoa	Food	Composites	A0C6P	Chocolate spread	The group includes Spreadable chocolate products based on cacao ingredients, different types of fats and very often nut derivatives. For example chocolate-hazelnut spread like nutella and other similar brands are included in this group
UIL.21.13440	Mars Minis	Food	Composites	A034R	Chocolate coated confectionery	The group includes any type of Chocolate coated confectionery, apart from the different bars present on the market and coated with chocolate that are included in a specific bar group.
UIL.21.13441	Extra Peppermint	Food	Composites	A035M#F04.A00XZ	Chewing gum, INGREDIENT = Mints	The group includes any type of Chewing gum, i.e. a confectionery product with the characteristic of being chewed but not swallowed. Aromatic fresh herb from the plant classified under the genus Mentha L. spp., commonly known as Mints.
UIL.21.13438	Snickers Minis	Food	Composites	A034R	Chocolate coated confectionery	The group includes any type of Chocolate coated confectionery, apart from the different bars present on the market and coated with chocolate that are included in a specific bar group.
UIL.21.13435	Kinder Joy	Food	Composites	A034R	Chocolate coated confectionery	The group includes any type of Chocolate coated confectionery, apart from the different bars present on the market and coated with chocolate that are included in a specific bar group.

Sample ID (from lab)	Name of Product & Brand	Food Item	FOOD TYPE FE2	FoodEx2 Code	FoodEx2 Description	Description
UIL.21.13454	Twix Minis	Food	Composites	A034R	Chocolate coated confectionery	The group includes any type of Chocolate coated confectionery, apart from the different bars present on the market and coated with chocolate that are included in a specific bar group.
UIL.21.13437	Kitkat	Food	Composites	A034R	Chocolate coated confectionery	The group includes any type of Chocolate coated confectionery, apart from the different bars present on the market and coated with chocolate that are included in a specific bar group.
UIL.21.13444	Mentos Sugar free Chewing Gum	Food	Composites	A035M#F10.A0CJD	Chewing gum, QUALITATIVE-INFO = Sugars-related info	The group includes any type of Chewing gum, i.e. a confectionery product with the characteristic of being chewed but not swallowed. Product non containing sugar or where sugar is only present in negligible amounts
UIL.21.13560	Cream Caramel Almaraei	Food	Composites	A06MY#F28.A07HJ	Cream brulee flavour, PROCESS = Caramelization / browning	Mixture of chemicals and/or extracts formulated in such a way to reproduce the original Cream brulee flavour.
UIL.21.13550	Danet Cream Caramel	Food	Composites	A06MY#F28.A07HJ	Cream brulee flavour, PROCESS = Caramelization / browning	Mixture of chemicals and/or extracts formulated in such a way to reproduce the original Cream brulee flavour.
UIL.21.13439	Bounty Minis	Food	Composites	A034R	Chocolate coated confectionery	The group includes any type of Chocolate coated confectionery, apart from the different bars present on the market and coated with chocolate that are included in a specific bar group.
UIL.21.14396	Daily Fresh/ Custard Powder	Food	Composites	A02PX#F10.A166Y	Custard, QUALITATIVE-INFO = Fresh	The group includes any type of Custard, such as Creme anglais and Pastry cream. A custard is a liquid thickened or set by the coagulation of egg protein. Since the group includes also desserts based on dairy imitates, information on the characterising ingredients has to be reported with additional facet descriptors.
UIL.21.13442	Batook Specialmint Chewing Gum	Food	Composites	A035M#F04.A00XZ	Chewing gum, INGREDIENT = Mints	The group includes any type of Chewing gum, i.e. a confectionery product with the characteristic of being chewed but not swallowed. Aromatic fresh herb from the plant classified under the genus Mentha L. spp., commonly known as Mints.
UIL.21.14409	Custard Powder/Tiara	Food	Composites	A02PX#F28.A07XY	Custard, PROCESS = Granulation (from powder)	The group includes any type of Custard, such as Creme anglais and Pastry cream. A custard is a liquid thickened or set by the coagulation of egg protein. Since the group includes also desserts based on dairy imitates, information on the characterising ingredients has to be reported with additional facet descriptors.

Sample ID (from lab)	Name of Product & Brand	Food Item	FOOD TYPE FE2	FoodEx2 Code	FoodEx2 Description	Description
UIL.21.14410	Custard Powder / Alalali	Food	Composites	A02PX#F28.A07XY	Custard, PROCESS = Granulation (from powder)	The group includes any type of Custard, such as Creme anglais and Pastry cream. A custard is a liquid thickened or set by the coagulation of egg protein. Since the group includes also desserts based on dairy imitates, information on the characterising ingredients has to be reported with additional facet descriptors.
UIL.21.13458	Nabil Cracker Salted Biscuits	Food	Composites	A005Y#F10.A0CJK	Crackers and breadsticks, QUALITATIVE-INFO = With added salt	The group includes any type of Crackers (i.e. grain flour dough layered and baked in form of salty biscuits) and dry breadsticks. Water crackers, soda crackers, biscuit crackers are included in this group.
UIL.21.14390	Mani (Mixed Nuts)	Food	RPC Derivatives	A0DYP#F10.A169R	Almonds and similar-, QUALITATIVE-INFO = Mixed origin	The group includes Almonds or similar nuts
UIL.21.17408	Cheez Balls (Mr. Krispy)	Food	Composites	A00FC#F04.A02YK	Corn chips, INGREDIENT = Cheese, cheddar	The group includes any type of Corn chips, i.e snacks usually made with cornmeal-based dough.
UIL.21.13433	Alrifai (Mixed Nuts)	Food	RPC Derivatives	A0DYP#F10.A169R	Almonds and similar-, QUALITATIVE-INFO = Mixed origin	The group includes Almonds or similar nuts
UIL.21.14388	Shai (Mix Nut)	Food	RPC Derivatives	A0DYP#F10.A169R	Almonds and similar-, QUALITATIVE-INFO = Mixed origin	The group includes Almonds or similar nuts
UIL.21.14384	Al Mudhish Puffed Corn Cheese Balls	Food	Composites	A006L#F04.A02QE	Puffed corn textured bread, INGREDIENT = Cheese	The group includes any type of bread-like products obtained by compacting puffed granules of corn.
UIL.21.13432	Best Salted Mixed Nuts	Food	RPC Derivatives	A0DYP#F10.A169R$F10.A0CJK	Almonds and similar-, QUALITATIVE-INFO = Mixed origin, QUALITATIVE-INFO = With added salt	The group includes Almonds or similar nuts
UIL.21.14418	Cheese Balls (Pofak Oman)	Food	Composites	A00FC#F04.A06LZ	Corn chips, INGREDIENT = Cheese flavour	The group includes any type of Corn chips, i.e snacks usually made with cornmeal-based dough.
UIL.21.14551	Cheese pastry (Dhahabi)	Food	Composites	A02QE#F04.A00CJ	Cheese, INGREDIENT = Various pastry	The group includes any type of Cheese and some additional products similar to cheese, like mascarpone and clotted cream.
UIL.21.13463	Cheetos / Flamin Hot	Food	Composites	A0EQX#F10.A0EZY	Chips/crisps, QUALITATIVE-INFO = Hot/piquant/spicy	The group includes any type of thin-fried slices or sticks of potatoes, or similar crunchy products obtained by cereal doughs or vegetables, used as snack

Sample ID (from lab)	Name of Product & Brand	Food Item	FOOD TYPE FE2	FoodEx2 Code	FoodEx2 Description	Description
UIL.21.13462	Pringles Original	Food	Composites	A0EQX	Chips/crisps	The group includes any type of thin-fried slices or sticks of potatoes, or similar crunchy products obtained by cereal doughs or vegetables, used as snack.
UIL.21.13431	American Garden Popcorn Butter	Food	RPC Derivatives	A00DC	Popcorn (maize, popped)	The group includes any type of Popcorn (popped maize) made by popping/puffing maize grains.
UIL.21.14389	Popcorn Cheese / American Garden	Food	Composites	A00DC#F04.A02QE	Popcorn (maize, popped), INGREDIENT = Cheese	The group includes any type of Popcorn (popped maize) made by popping/puffing maize grains.
UIL.21.14560	Sohar Chips	Food	Composites	A011L	Potato crisps or sticks	The group includes any type of Potato crisps (also known as chips in American English), made of thin slices of potatoes or potato-based doughs, included those in form of small sticks.
UIL.21.14383	Lay's (Salt)	Food	Composites	A011L#F10.A0CJK	Potato crisps or sticks, QUALITATIVE-INFO = With added salt	The group includes any type of Potato crisps (also known as chips in American English), made of thin slices of potatoes or potato-based doughs, included those in form of small sticks.
UIL.21.14385	potato chips ready salted	Food	Composites	A011L#F10.A0CJF	Potato crisps or sticks, QUALITATIVE-INFO = Salt (sodium)-related info	The group includes any type of Potato crisps (also known as chips in American English), made of thin slices of potatoes or potato-based doughs, included those in form of small sticks.
UIL.21.14386	chips Oman	Food	Composites	A011L	Potato crisps or sticks	The group includes any type of Potato crisps (also known as chips in American English), made of thin slices of potatoes or potato-based doughs, included those in form of small sticks.
UIL.21.13833	vanilla ice cream (Igloo)	Food	Composites	A02QA#F04.A06VS	Ice cream, milk-based, INGREDIENT = Vanilla flavour	The group includes any type of Ice cream, milk-based (no milk-imitate-based).
UIL.21.13832	vanilla ice cream (Mazoon)	Food	Composites	A02QA#F04.A06VS	Ice cream, milk-based, INGREDIENT = Vanilla flavour	The group includes any type of Ice cream, milk-based (no milk-imitate-based).
UIL.21.13299	NESTLE Chocapic	Food	Composites	A00EL#F04.A06MF	Mixed breakfast cereals, INGREDIENT = Chocolate flavour	The group includes any type of Mixed breakfast cereals, including mixed muesli, usually made by cereal grains mixed with other ingredients, like raisins, chocolate, nuts and dried fruit.
UIL.21.13300	KELLOGG'S Coco Pops	Food	Composites	A00EL#F04.A06MF	Mixed breakfast cereals, INGREDIENT = Chocolate flavour	The group includes any type of Mixed breakfast cereals, including mixed muesli, usually made by cereal grains mixed with other ingredients, like raisins, chocolate, nuts and dried fruit.
UIL.21.14387	Kellogg's special (cereal)	Food	Composites	A00EL	Mixed breakfast cereals	The group includes any type of Mixed breakfast cereals, including mixed muesli, usually made by cereal grains mixed with other ingredients, like raisins, chocolate, nuts and dried fruit.

Sample ID (from lab)	Name of Product & Brand	Food Item	FOOD TYPE FE2	FoodEx2 Code	FoodEx2 Description	Description
UIL.21.13298	Nestle Gold Corn Flakes	Food	Composites	A00EL	Mixed breakfast cereals	The group includes any type of Mixed breakfast cereals, including mixed muesli, usually made by cereal grains mixed with other ingredients, like raisins, chocolate, nuts and dried fruit.
UIL.21.13459	Original Oreo	Food	Composites	A009Z	Biscuits, chocolate	The group includes any type of cocoa powder-based biscuits. This term should not be used for chocolate filled biscuits and biscuits containing pieces of chocolate.
UIL.21.13457	Lotus Biscoff	Food	Composites	A009V	Biscuits	The group includes any type of Biscuits and cookies (sweet and semi-sweet).
UIL.21.13453	Tiffany Crunch And Cream	Food	Composites	A00AE	Biscuit with inclusions, filling or coating	The group includes any type of filled biscuit, i.e. biscuits with inclusions, filling or coating such as American cookies.
UIL.21.13452	Nutro Chocolate Flavoured Cream Wafers	Food	Composites	A0BYR#F04.A034L	Wafers, INGREDIENT = Cream chocolate	The group includes any type of plain wafers (i.e. without inclusions, fillings or coatings) and wafer-based biscuits.
UIL.21.13451	Britannia Chocolate Flavoured Premium Crème Wafer	Food	Composites	A0BYR#F04.A034L	Wafers, INGREDIENT = Cream chocolate	The group includes any type of plain wafers (i.e. without inclusions, fillings or coatings) and wafer-based biscuits.
UIL.21.13460	Americana Premium Butter Cookies	Food	Composites	A009Y	Butter biscuits	The group includes any type of Butter biscuits such as småkage and Danish cookies.
UIL.21.14567	Switz Mini Cup Cake Vanilla	Food	Composites	A00AN#F04.A019H	Cakes, INGREDIENT = Vanilla	The group includes any type of cake, i.e. sweets generally constituted by baked sweet dough/batter and possibly containing creams or fillings of different nature, regardless the size.
UIL.21.14566	Atyab Twin Cake Vanilla	Food	Composites	A00AN#F04.A019H	Cakes, INGREDIENT = Vanilla	The group includes any type of cake, i.e. sweets generally constituted by baked sweet dough/batter and possibly containing creams or fillings of different nature, regardless the size.
UIL.21.13461	Danish Butter Cookies	Food	Composites	A009Y	Butter biscuits	The group includes any type of Butter biscuits such as småkage and Danish cookies.
UIL.21.13449	Tiffany Everyday Nice (Sugar Sprinkled Coconut Biscuits)	Food	Composites	A009V#F28.A07HN$F04.A014K$F04.A032H	Biscuits, PROCESS = Sugar coating, INGREDIENT = Coconuts, INGREDIENT = Sucrose (common sugar)	The group includes any type of Biscuits and cookies (sweet and semi-sweet).
UIL.21.13448	Tiffany Glucose (Milk and Honey Biscuits)	Food	Composites	A009V#F04.A06PQ$F04.A02LT	Biscuits, INGREDIENT = Honey flavour, INGREDIENT = Milk	The group includes any type of Biscuits and cookies (sweet and semi-sweet).

Sample ID (from lab)	Name of Product & Brand	Food Item	FOOD TYPE FE2	FoodEx2 Code	FoodEx2 Description	Description
UIL.21.13447	Nabil Nice (sugar sprinkled coconut biscuits)	Food	Composites	A009V#F28.A07HN$F04.A014K$F04.A032H	Biscuits, PROCESS = Sugar coating, INGREDIENT = Coconuts, INGREDIENT = Sucrose (common sugar)	The group includes any type of Biscuits and cookies (sweet and semi-sweet).
UIL.21.13446	Nabil cream wafers (chocolate flavour)	Food	Composites	A0BYR#F04.A034L	Wafers, INGREDIENT = Cream chocolate	The group includes any type of plain wafers (i.e. without inclusions, fillings or coatings) and wafer-based biscuits.
UIL.21.17407	Donut (Switz)	Food	Composites	A00BR	Doughnuts-berliner	The group includes any type of Doughnuts-berliner, usually consisting of a fried sweet dough. Baked (not fried) variants are also present on the market.
UIL.21.17405	Apple puff (Lusine)	Food	Composites	A00CJ#F04.A01DJ	Various pastry, INGREDIENT = Apples	The group includes various pastries not listed elsewhere.
UIL.21.14574	7 days mini croissant	Food	Composites	A00BM	Croissant	The group includes any type of Croissant, made of a layered yeast-leavened dough with unspecified filling (eventually also without).
UIL.21.14552	Apple pastry	Food	Composites	A00CJ#F04.A01DJ	Various pastry, INGREDIENT = Apples	The group includes various pastries not listed elsewhere.
UIL.21.13445	Nabil glucose	Food	Composites	A009V	Biscuits	The group includes any type of Biscuits and cookies (sweet and semi-sweet).
UIL.21.14568	Chocolate jumbo croissant	Food	Composites	A00BN	Croissant, filled with chocolate	The group includes any type of Croissant, filled with chocolate.
UIL.21.17531	Croissant (Dhahabi)	Food	Composites	A00BM	Croissant	The group includes any type of Croissant, made of a layered yeast-leavened dough with unspecified filling (eventually also without).
UIL.21.17406	Cheese Puff (Lusine)	Food	Composites	A00CJ#F04.A02QE	Various pastry, INGREDIENT = Cheese	The group includes various pastries not listed elsewhere.
UIL.21.13548	Almarai Strawberry Laban	Drink	RPC Derivatives	A02NQ#F04.A06TV	Yoghurt drinks, including sweetened and/or flavoured variants, INGREDIENT = Strawberry flavour	The group includes any type of Yoghurt drinks (liquid consistency), including sweetened and/or flavoured variants. Flavouring may be considered as minor ingredient.
UIL.21.13547	Mazoon Strawberry Laban	Drink	RPC Derivatives	A02NQ#F04.A06TV	Yoghurt drinks, including sweetened and/or flavoured variants, INGREDIENT = Strawberry flavour	The group includes any type of Yoghurt drinks (liquid consistency), including sweetened and/or flavoured variants. Flavouring may be considered as minor ingredient.

Sample ID (from lab)	Name of Product & Brand	Food Item	FOOD TYPE FE2	FoodEx2 Code	FoodEx2 Description	Description
UIL.21.13592	Fresh Labneh, Full Fat Al-Marai	Food	RPC Derivatives	A16GH#F10.A077A	Strained yoghurt, QUALITATIVE-INFO = Full fat	The group includes any type of Strained yoghurt, i.e. yogurt that has been strained to remove its whey, resulting in a thicker consistency than unstrained yogurt. For example: Greek yoghurt, Labneh and Yogurt cheese are included in this group. The part consumed/analysed is by default the whole or a portion of it representing the observed heterogeneity.
UIL.21.13594	Fresh Labneh: Full Fat Mazoon	Food	RPC Derivatives	A16GH#F10.A077A	Strained yoghurt, QUALITATIVE-INFO = Full fat	The group includes any type of Strained yoghurt, i.e. yogurt that has been strained to remove its whey, resulting in a thicker consistency than unstrained yogurt. For example: Greek yoghurt, Labneh and Yogurt cheese are included in this group. The part consumed/analysed is by default the whole or a portion of it representing the observed heterogeneity.
UIL.21.13593	Labneh Turkish Recipe: Puck	Food	RPC Derivatives	A16GH	Strained yoghurt	The group includes any type of Strained yoghurt, i.e. yogurt that has been strained to remove its whey, resulting in a thicker consistency than unstrained yogurt. For example: Greek yoghurt, Labneh and Yogurt cheese are included in this group. The part consumed/analysed is by default the whole or a portion of it representing the observed heterogeneity.
UIL.21.13558	Fresh Yougurt Full Fat: Al-Marai	Drink	RPC Derivatives	A02NE#F10.A077A$F10.A166Y	Yoghurt, QUALITATIVE-INFO = Full fat, QUALITATIVE-INFO = Fresh	The group includes any type of Yoghurt.
UIL.21.13555	Mazoon Fresh Yoghurt Full Fat	Food	RPC Derivatives	A02NE#F10.A077A$F10.A166Y	Yoghurt, QUALITATIVE-INFO = Full fat, QUALITATIVE-INFO = Fresh	The group includes any type of Yoghurt.
UIL.21.13559	Youghurt;Full Cream AlRawabi	Drink	RPC Derivatives	A02NE#F10.A077A	Yoghurt, QUALITATIVE-INFO = Full fat	The group includes any type of Yoghurt.
UIL.21.13556	Fresh Yougurt Full Fat: Al-Safwah	Drink	RPC Derivatives	A02NE#F10.A077A$F10.A166Y	Yoghurt, QUALITATIVE-INFO = Full fat, QUALITATIVE-INFO = Fresh	The group includes any type of Yoghurt.
UIL.21.13542	Mazoon Fresh Laban Full Fat	Drink	RPC Derivatives	A02MV#F10.A077A$F10.A166Y	Buttermilk, QUALITATIVE-INFO = Full fat, QUALITATIVE-INFO = Fresh	The group includes any type of Buttermilk.
UIL.21.17403	Activia Full Fat Fresh Laban	Drink	RPC Derivatives	A02NN	Yoghurt, goat milk, plain	The group includes any type of Yoghurt.

Sample ID (from lab)	Name of Product & Brand	Food Item	FOOD TYPE FE2	FoodEx2 Code	FoodEx2 Description	Description
UIL.21.13557	Yoghurt: Full Cream Unikai	Food	RPC Derivatives	A02NE#F10.A077A	Yoghurt, QUALITATIVE-INFO = Full fat	The group includes any type of Yoghurt.
UIL.21.13544	Almarai Fresh Laban Full Fat	Drink	RPC Derivatives	A02MV#F10.A077A$F10.A166Y	Buttermilk, QUALITATIVE-INFO = Full fat, QUALITATIVE-INFO = Fresh	The group includes any type of Buttermilk.
UIL.21.13543	Laban Fresh Full Cream (Asafwah)	Drink	RPC Derivatives	A02MV#F10.A077A$F10.A166Y	Buttermilk, QUALITATIVE-INFO = Full fat, QUALITATIVE-INFO = Fresh	The group includes any type of Buttermilk.
UIL.21.13545	Almarai Laban Up	Drink	RPC Derivatives	A02MV	Buttermilk	The group includes any type of Buttermilk.
UIL.21.13539	Fresh Laban (Alrawabi)	Drink	RPC Derivatives	A02MV#F10.A166Y	Buttermilk, QUALITATIVE-INFO = Fresh	The group includes any type of Buttermilk.
UIL.21.13541	Laban Up (Alsafwa)	Drink	RPC Derivatives	A02MV	Buttermilk	The group includes any type of Buttermilk.
UIL.21.13540	Mazoon Laban Up	Drink	RPC Derivatives	A02MV	Buttermilk	The group includes any type of Buttermilk.
UIL.21.17409	Activia Full Fat Plain Yoghurt	Food	RPC Derivatives	A02NE#F10.A077A	Yoghurt, QUALITATIVE-INFO = Full fat	The group includes any type of Yoghurt.
UIL.21.14131	Cream (Luna)	Food	RPC Derivatives	A02MK	Cream and cream products	The group includes any type of Cream, i.e. the portion of milk with enhanced fat content, usually obtained by centrifugal separation of the skim milk.
UIL.21.13546	Alrawabi Up: Laban Drink	Drink	RPC Derivatives	A02MV	Buttermilk	The group includes any type of Buttermilk.
UIL.21.13549	Laban Drink (Unikai)	Drink	RPC Derivatives	A02MV	Buttermilk	The group includes any type of Buttermilk.
UIL.21.14180	Mazoon Sambharam Spicy Laban Drink	Drink	RPC Derivatives	A02MV#F10.A0EZY	Buttermilk, QUALITATIVE-INFO = Hot/piquant/spicy	The group includes any type of Buttermilk.
UIL.21.14179	Laban Drinks (Alkhamayil)	Drink	RPC Derivatives	A02MV	Buttermilk	The group includes any type of Buttermilk.
UIL.21.17404	Balade Farms Ayran Original Laban	Drink	RPC Derivatives	A02MV	Buttermilk	The group includes any type of Buttermilk.
UIL.21.14553	Cream (Puck)	Food	RPC Derivatives	A02MK	Cream and cream products	The group includes any type of Cream, i.e. the portion of milk with enhanced fat content, usually obtained by centrifugal separation of the skim milk.
UIL.21.13830	Nestle cream	Food	RPC Derivatives	A02MK	Cream and cream products	The group includes any type of Cream, i.e. the portion of milk with enhanced fat content, usually obtained by centrifugal separation of the skim milk and eventual adjustment of the final fat content.
UIL.21.14572	Chedder cheese (Almarai)	Food	RPC Derivatives	A02YK	Cheese, cheddar	The group includes any type of cheddar cheese, a type of hard cheese.

Sample ID (from lab)	Name of Product & Brand	Food Item	FOOD TYPE FE2	FoodEx2 Code	FoodEx2 Description	Description
UIL.21.13835	Haloumi Pinar	Food	RPC Derivatives	A065R	Halloumi	The group includes any type of halloumi, a Cypriot semi-hard, unripened brined cheese made from a mixture of goat's and sheep's milk, and sometimes also cow's milk. Often consumed fried or grilled.
UIL.21.14571	Chedder cheese (Kraft)	Food	RPC Derivatives	A02YK	Cheese, cheddar	The group includes any type of cheddar cheese, a type of hard cheese.
UIL.21.13840	Almarai Burger Slice Cheese	Food	RPC Derivatives	A031B	Processed cheese, sliceable	The group includes any type of Processed cheese, sliceable.
UIL.21.13839	Kraft Slice Cheese Original	Food	RPC Derivatives	A031B	Processed cheese, sliceable	The group includes any type of Processed cheese, sliceable. The ingredients added to melt and eventually flavour the product may be considered minor ingredients.
UIL.21.13834	Haloumi Cheese Al-Marai	Food	RPC Derivatives	A065R	Halloumi	The group includes any type of halloumi, a Cypriot semi-hard, unripened brined cheese made from a mixture of goat's and sheep's milk, and sometimes also cow's milk.
UIL.21.13848	Kraft Cheddar Cheese Spread Original	Food	RPC Derivatives	A031C#F04.A02YK	Processed cheese, spreadable, INGREDIENT = Cheese, cheddar	The group includes any type of Processed cheese, spreadable. The ingredients added to melt and eventually flavour the product may be considered minor ingredients.
UIL.21.13844	Almarai Cream Cheese Cheddar	Food	RPC Derivatives	A02QZ#F04.A02YK	Cream cheese, INGREDIENT = Cheese, cheddar	The group includes any type of Cream cheese, a fresh cheese with creamy texture, such as philadelfia or boursin.
UIL.21.13838	Cheese Triangles Puck	Food	RPC Derivatives	A02QE	Cheese	The group includes any type of cheese and some additional products similar to cheese, like mascarpone and clotted cream. Cheese is produced through the coagulation of milk protein (casein), which is separated from the milk's whey.
UIL.21.13837	Cheese Triangles Al Maraai	Food	RPC Derivatives	A02QE	Cheese	The group includes any type of Cheese and some additional products similar to cheese, like mascarpone and clotted cream. Cheese is produced through the coagulation of milk protein (casein), which is separated from the milk's whey.
UIL.21.13847	Puck Cream Cheese	Food	RPC Derivatives	A02QZ	Cream cheese	The group includes any type of Cream cheese, a fresh cheese with creamy texture, such as philadelfia or boursin.
UIL.21.13846	Puck Cream Cheese Cheddar	Food	RPC Derivatives	A02QZ#F04.A02YK	Cream cheese, INGREDIENT = Cheese, cheddar	The group includes any type of Cream cheese, a fresh cheese with creamy texture, such as philadelfia or boursin.
UIL.21.13836	Triangle Cheese Abu Alwald	Food	RPC Derivatives	A02QE	Cheese	The group includes any type of Cheese and some additional products similar to cheese, like mascarpone and clotted cream. Cheese is produced through the coagulation of milk protein (casein), which is separated from the milk's whey.
UIL.21.13845	Almarai Cream Cheese	Food	RPC Derivatives	A02QZ	Cream cheese	The group includes any type of Cream cheese, a fresh cheese with creamy texture, such as philadelfia or boursin.

Sample ID (from lab)	Name of Product & Brand	Food Item	FOOD TYPE FE2	FoodEx2 Code	FoodEx2 Description	Description
UIL.21.14554	Pinar Processed Cream Cheese Spread	Food	RPC Derivatives	A031C#F10.A077F	Processed cheese, spreadable, QUALITATIVE-INFO = Single cream	The group includes any type of Processed cheese, spreadable. The ingredients added to melt and eventually flavour the product may be considered minor ingredients.
UIL.21.13841	Puck Slice Cheese Original	Food	RPC Derivatives	A031B	Processed cheese, sliceable	The group includes any type of Processed cheese, sliceable. The ingredients added to melt and eventually flavour the product may be considered minor ingredients.
UIL.21.13843	Mazoon Spread Cheese	Food	RPC Derivatives	A031C	Processed cheese, spreadable	The group includes any type of Processed cheese, spreadable. The ingredients added to melt and eventually flavour the product may be considered minor ingredients.
UIL.21.13842	Mazoon Spread Cheese Cheddar	Food	RPC Derivatives	A031C#F04.A02YK	Processed cheese, spreadable, INGREDIENT = Cheese, cheddar	The group includes any type of Processed cheese, spreadable. The ingredients added to melt and eventually flavour the product may be considered minor ingredients.
UIL.21.13456	Britannia Toastea Wheat Rusk	Food	Composites	A006M#F04.A003X	Rusk, INGREDIENT = Wheat flour	The group includes any type of Rusk, which is a sweet sliced bread browned in an oven.
UIL.21.13455	Tiffany Whole Wheat Rusks	Food	Composites	A006M#F04.A003X$F10.A06HR	Rusk, INGREDIENT = Wheat flour, QUALITATIVE-INFO = Integral /not refined	The group includes any type of Rusk, which is a sweet sliced bread browned in an oven.
UIL.21.13429	Indomie Fried Noodles	Food	Composites	A007R#F28.A07GR	Asian-style noodles other than glass noodles, PROCESS = Frying	The group covers any type of Asian-style noodles other than glass noodles (transparent noodles).
UIL.21.14573	Family Corned Beef	Food	Composites	A0B9G	Corned beef, cooked	The group includes any type of Corned beef, cooked.
UIL.21.14202	Al-Islami Ground Beef	Food	RPC Derivatives	A023C	Ham, beef	The group includes any type of Ham from beef, usually made by curing bovine meat.
UIL.21.14201	Corned Beef (Taybat)	Food	Composites	A0B9G	Corned beef, cooked	The group includes any type of Corned beef, cooked.
UIL.21.14205	Al-Safa Chicken Franks	Food	Composites	A024F#F04.A04DS	Sausages, INGREDIENT = Chicken, minced meat	The group includes any type of Sausages, i.e. more or less coarse mixtures of lean meats, fatty tissues and other ingredients, often filled into casings.
UIL.21.14200	Beef Burger (Taybat)	Food	Composites	A03ZL	Hamburger with bread	The group includes any type of Hamburger sandwich, i.e. hamburger meat with bread.
UIL.21.14204	Americana Beef Hot dog	Food	Composites	A024F#F04.A0EYM	Sausages, INGREDIENT = Charcuterie meat products	The group includes any type of Hot dog with bread, usually consisting in a cooked sausage stuffed in a sliced bun.
UIL.21.14198	Sadia Chicken Burger	Food	Composites	A03XF#F04.A04DS	Meat burger (no sandwich), INGREDIENT=Chicken, minced meat	The group includes any type of Meat burger (no sandwich), i.e. patties of ground meat.

Sample ID (from lab)	Name of Product & Brand	Food Item	FOOD TYPE FE2	FoodEx2 Code	FoodEx2 Description	Description
UIL.21.14203	Al-Kabeer Jumbo Hot Dogs	Food	Composites	A024F	Sausages	The group includes any type of Sausages, i.e. mixtures of lean meats, fatty tissues often filled into casings.
UIL.21.14194	Al-Safa Breaded Chicken Finger	Food	Composites	A04DS#F28.A07HK	Chicken, minced meat, PROCESS = Breading	The group includes any type of chicken/fowl minced meat.
UIL.21.14206	Sadia Chicken Franks	Food	Composites	A024F#F04.A04DS	Sausages, INGREDIENT = Chicken, minced meat	The group includes any type of Sausages, i.e. more or less coarse mixtures of lean meats, fatty tissues and other ingredients, often filled into casings.
UIL.21.14199	Beef Burger Sadia	Food	Composites	A03ZL	Hamburger with bread	The group includes any type of Hamburger sandwich, i.e. hamburger meat with bread.
UIL.21.14195	Sadia Chicken Nuggets Crispy	Food	Composites	A04DS#F28.A07HK	Chicken, minced meat, PROCESS = Breading	The group includes any type of chicken/fowl minced meat.
UIL.21.14196	Al-Safa Chicken Burger	Food	Composites	A03XF#F04.A04DS	Meat burger (no sandwich), INGREDIENT = Chicken, minced meat	The group includes any type of Meat burger (no sandwich), i.e. patties of ground meat.
UIL.21.13292	Tuna Alalali In Water	Food	RPC Derivatives	A0FBT#F28.A07GN	Canned tunas and similar, PROCESS = Bain-marie cooking (in water bath)	The group includes any type of Canned tunas and similar.
UIL.21.13294	Skipjack Tuna California Garden In Sun Flower Oil	Food	RPC Derivatives	A0FBT#F27.A02EF$F04.A015F	Canned tunas and similar, SOURCE-COMMODITIES = Tuna, skipjack, INGREDIENT = Oilseeds	The group includes any type of Canned tunas and similar.
UIL.21.13295	Light Chunks Tuna California Garden In Water	Food	RPC Derivatives	A0FBT#F28.A07KT$F28.A07GN	Canned tunas and similar, PROCESS = Portioning, PROCESS = Bain-marie cooking (in water bath)	The group includes any type of Canned tunas and similar.
UIL.21.13291	Skipjack Tuna Alalali In Sun Flower Oil	Food	RPC Derivatives	A0FBT#F27.A02EF$F04.A015F	Canned tunas and similar, SOURCE-COMMODITIES = Tuna, skipjack, INGREDIENT = Oilseeds	The group includes any type of Canned tunas and similar.
UIL.21.13293	White Tuna Meat In Sun Flower Oil Delmonte	Food	RPC Derivatives	A0FBT#F04.A015L$F10.A0F2R	Canned tunas and similar, INGREDIENT = Sunflower seeds, QUALITATIVE-INFO = White	The group includes any type of Canned tunas and similar.
UIL.21.13434	White Tuna Meat In Brine	Food	RPC Derivatives	A0FBT#F28.A07KC$F10.A0F2R	Canned tunas and similar, PROCESS = Pickling, QUALITATIVE-INFO = White	The group includes any type of Canned tunas and similar.
UIL.21.14397	Diamond Strawberry Jam	Food	Composites	A01MN	Jam, strawberries	The group includes any type of Jam obtained from Strawberries.

Sample ID (from lab)	Name of Product & Brand	Food Item	FOOD TYPE FE2	FoodEx2 Code	FoodEx2 Description	Description
UIL.21.14405	Mango Thokku Pickle In Oil / Priya	Food	Composites	A046C#F04.A015F	Mango pickle, INGREDIENT = Oilseeds	The group includes any type of Mango pickle.
UIL.21.14404	Tomato Paste / Alalali	Food	RPC Derivatives	A00ZF	Tomato paste	The group includes any type of Tomato paste or concentrate. The part consumed/analysed is by default the whole or a portion of it representing the observed heterogeneity.
UIL.21.13278	Baked Beans California Garden	Food	RPC Derivatives	A01BB#F28.A07GX	Canned or jarred common beans, PROCESS = Baking	The group includes any type of canned or jarred common beans.
UIL.21.14399	Tomato Paste / Almudhish	Food	RPC Derivatives	A00ZF	Tomato paste	The group includes any type of Tomato paste or concentrate. The part consumed/analysed is by default the whole or a portion of it representing the observed heterogeneity.
UIL.21.13276	Green Giant Sweet Corn	Food	RPC Derivatives	A00ZP	Sweet corn canned	The group includes any type of Sweet corn canned.
UIL.21.13290	Sweet Corn American Garden	Food	RPC Derivatives	A00ZP	Sweet corn canned	The group includes any type of Sweet corn canned.
UIL.21.13282	Baked Beans Almazraa	Food	RPC Derivatives	A01BB#F28.A07GX	Canned or jarred common beans, PROCESS = Baking	The group includes any type of canned or jarred common beans.
UIL.21.14400	Tomato Paste / Jumbo	Food	RPC Derivatives	A00ZF	Tomato paste	The group includes any type of Tomato paste or concentrate. The part consumed/analysed is by default the whole or a portion of it representing the observed heterogeneity.
UIL.21.14556	Luna Baked Beans In Tomato Sauce	Food	RPC Derivatives	A01BB#F28.A07GX$F04.A044C	Canned or jarred common beans, PROCESS = Baking, INGREDIENT = Tomato-containing cooked sauces	The group includes any type of canned or jarred common beans.
UIL.21.13280	Sweet Corn California Garden	Food	RPC Derivatives	A00ZP	Sweet corn canned	The group includes any type of Sweet corn canned.
UIL.21.14559	Peas processed	Food	RPC Derivatives	A012G#F28.A0C0R	Peas (without pods) and similar-, PROCESS = Processed	The group includes Peas, also known as green pea, or similar legumes
UIL.21.14555	La Ming Beans In Tomato Sauce	Food	RPC Derivatives	A01BB#F04.A044C	Canned or jarred common beans, INGREDIENT = Tomato-containing cooked sauces	The group includes any type of canned or jarred common beans.
UIL.21.13288	Sweet Corn Libbys	Food	RPC Derivatives	A00ZP	Sweet corn canned	The group includes any type of Sweet corn canned.
UIL.21.13279	Processed Peas California Garden	Food	RPC Derivatives	A012G#F28.A0C0R	Peas (without pods) and similar-, PROCESS = Processed	The group includes Peas, also known as green pea, or similar legumes

Sample ID (from lab)	Name of Product & Brand	Food Item	FOOD TYPE FE2	FoodEx2 Code	FoodEx2 Description	Description
UIL.21.13287	Processed Peas Almazraa	Food	RPC Derivatives	A012G#F28.A0C0R	Peas (without pods) and similar-, PROCESS = Processed	The group includes Peas, also known as green pea, or similar legumes
UIL.21.14557	Foul Medammas (American Garden)	Food	RPC Derivatives	A012A#F28.A0BYP	Broad beans (without pods), PROCESS = Canning / jarring	Legumes without pod from the plant classified under the species Vicia faba L. (any subspp. and var.), commonly known as Broad beans or Fava beans or Horse beans or Tic beans.
UIL.21.14558	Lunafoul Medames Extra Grade Fava Beans	Food	RPC Derivatives	A012A#F28.A0BYP	Broad beans (without pods), PROCESS = Canning / jarring	Legumes without pod from the plant classified under the species Vicia faba L. (any subspp. and var.), commonly known as Broad beans or Fava beans or Horse beans or Tic beans.
UIL.21.13277	Plain Medammes California Garden	Food	RPC Derivatives	A012A#F28.A0BYP	Broad beans (without pods), PROCESS = Canning / jarring	Legumes without pod from the plant classified under the species Vicia faba L. (any subspp. and var.), commonly known as Broad beans or Fava beans or Horse beans or Tic beans.
UIL.21.13281	Chick Peas California Garden	Food	RPC Derivatives	A01BE	Canned or jarred chickpea	The group includes any type of canned or jarred chick pea.
UIL.21.13286	Chick Peas Almazraa	Food	RPC Derivatives	A01BE	Canned or jarred chickpea	The group includes any type of canned or jarred chick pea.
UIL.21.13283	Foul Medamas Almazraa	Food	RPC Derivatives	A012A#F28.A0BYP	Broad beans (without pods), PROCESS = Canning / jarring	Legumes without pod from the plant classified under the species Vicia faba L. (any subspp. and var.), commonly known as Broad beans or Fava beans or Horse beans or Tic beans.
UIL.21.13284	Chick Peas Luna	Food	RPC Derivatives	A01BE	Canned or jarred chickpea	The group includes any type of canned or jarred chick pea.
UIL.21.13289	Boildchick Peas Mara	Food	RPC Derivatives	A01BE#F28.A07GL	Canned or jarred chickpea, PROCESS = Boiling	The group includes any type of canned or jarred chick pea.
UIL.21.13285	Green Peas Luna	Food	RPC Derivatives	A01BC#F10.A0F2Q	Canned or jarred peas, QUALITATIVE-INFO = Green	The group includes any type of canned or jarred peas.
UIL.21.14403	Mango Pickles In Oil / Ahmed	Food	Composites	A046C#F04.A015F	Mango pickle, INGREDIENT = Oilseeds	The group includes any type of Mango pickle.
UIL.21.14406	Mango Pickle / Aeroplane	Food	Composites	A046C	Mango pickle	The group includes any type of Mango pickle.
UIL.21.14394	American Garden BBQ Sauce Original	Food	Composites	A045B#F02.A0EPG	Barbecue or steak sauces, PART-NATURE = BBQ-type sauces (as part-nature)	The group includes any type of Barbecue sauce or steak sauces, usually used as condiment or marinade for grilled/barbecued meat.
UIL.21.14395	Heinz Classic BBQ Smokey And Rich	Food	Composites	A045B#F02.A0EPG	Barbecue or steak sauces, PART-NATURE = BBQ-type sauces (as part-nature)	The group includes any type of Barbecue sauce or steak sauces, usually used as condiment or marinade for grilled/barbecued meat.
UIL.21.14411	Tomato Ketchup/ Jumbo	Food	Composites	A044P	Tomato ketchup and related sauces	The group includes any type of Tomato ketchup (typically made from tomatoes, a sweetener, vinegar) and derived sauces.
UIL.21.14413	Tomato Ketchup / Heinz	Food	Composites	A044P	Tomato ketchup and related sauces	The group includes any type of Tomato ketchup (typically made from tomatoes, a sweetener, vinegar) and derived sauces.

Sample ID (from lab)	Name of Product & Brand	Food Item	FOOD TYPE FE2	FoodEx2 Code	FoodEx2 Description	Description
UIL.21.14408	Tomato Ketchup / Hayat	Food	Composites	A044P	Tomato ketchup and related sauces	The group includes any type of Tomato ketchup (typically made from tomatoes, a sweetener, vinegar) and derived sauces.
UIL.21.14392	Delicio French Dressing	Food	Composites	A045K	Salad dressing	The group includes any type of Salad dressing.
UIL.21.14393	Delicio 1000 Island	Food	Composites	A045K	Salad dressing	The group includes any type of Salad dressing.
UIL.21.14402	Hot Sauce / Excellence	Food	Composites	A044Q	Tabasco sauce	The group includes any type of Tabasco sauce, i.e. a sauce based on tabasco peppers
UIL.21.13430	Maggi Chicken Stock	Food	Composites	A043G	Stock cubes or granulate, chicken	The group includes any type of chicken stock cubes or granulate.
UIL.21.14407	Pizza Sauce Olive& Mushrooms / Alalali	Food	Composites	A044C#F04.A01BP$F04.A06RJ	Tomato-containing cooked sauces, INGREDIENT = Table olives, INGREDIENT = Mushroom flavour	The group includes any type of Tomato-containing cooked sauce
UIL.21.14415	Classic Mayonnaise / Mazola	Food	Composites	A044X	Mayonnaise sauce	The group includes any type of Mayonnaise sauce, usually made with vegetable oil and egg yolk.
UIL.21.14416	Creamy Classic Mayonnaise / Heinz	Food	Composites	A044X	Mayonnaise sauce	The group includes any type of Mayonnaise sauce, usually made with vegetable oil and egg yolk.
UIL.21.14401	Tabasco Hot Sauce	Food	Composites	A044Q	Tabasco sauce	The group includes any type of Tabasco sauce, i.e. a sauce based on tabasco peppers
UIL.21.14398	Soy Sauce Classic / American Garden	Food	Composites	A044R	Soy sauce	The group includes any type of Soy sauce (Soya sauce, Tamari), made from fermented soybeans and other ingredients
UIL.21.14417	Hot Sause / American Garden	Food	Composites	A044Q	Tabasco sauce	The group includes any type of Tabasco sauce, i.e. a sauce based on tabasco peppers
UIL.21.13296	Hayat Hot Sauce	Food	Composites	A044Q	Tabasco sauce	The group includes any type of Tabasco sauce, i.e. a sauce based on tabasco peppers
UIL.21.13297	Jumbo Hot Sauce	Food	Composites	A044Q	Tabasco sauce	The group includes any type of Tabasco sauce, i.e. a sauce based on tabasco peppers
UIL.21.14564	Maggi Beef Flavour	Food	Composites	A043H	Stock cube or granulate, beef	The group includes any type of beef stock cubes or granulate.
UIL.21.14565	Maggi Chicken Stock Less Salt	Food	Composites	A043G#F10.A0B8M	Stock cubes or granulate, chicken, QUALITATIVE-INFO = Low sodium / reduced salt	The group includes any type of chicken stock cubes or granulate
UIL.21.14561	Knour CHICKEN Stock	Food	Composites	A043G	Stock cubes or granulate, chicken	The group includes any type of chicken stock cubes or granulate
UIL.21.14562	Knour Beef Flavoured Stock	Food	Composites	A043H	Stock cube or granulate, beef	The group includes any type of beef stock cubes or granulate.
UIL.21.14563	Knour Vegetable Stock Cubes	Food	Composites	A043J	Stock cubes or granulate, vegetables	The group includes any type of vegetable stock cubes or granulate.

Sample ID (from lab)	Name of Product & Brand	Food Item	FOOD TYPE FE2	FoodEx2 Code	FoodEx2 Description	Description
UIL.21.17402	Tang (Orange) (Powder)	Food	Composites	A03FJ#F28.A07XY	Soft drink, orange flavour, PROCESS = Granulation (from powder)	The group includes any type of soft drink with orange flavour.
UIL.21.13850	Al Deyafa Orange Flavour (Powder)	Food	RPC Derivatives	A03FJ#F28.A07MR	Soft drink, orange flavour, PROCESS = Reconstitution from concentrate, powder or other dehydrated form	The group includes any type of soft drink with orange flavour.
UIL.21.13828	Al Deyafa Fruit Cordial	Drink	RPC Derivatives	A03AP	Juice, mixed fruit	The group includes any type of Juice from mixed fruit.
UIL.21.14138	Al Rawabi Red Grape Juice	Drink	RPC Derivatives	A03AK#F10.A077K$F10.A0F2S	Juice, grape, QUALITATIVE-INFO = Without added sugar, QUALITATIVE-INFO = Red	The group includes any type of juice made from Grape including
UIL.21.14135	Almarai Graps And Berries (Farms Select)	Drink	RPC Derivatives	A03BA	Juice, berry-grape	The group includes any type of juice based on grape juice with variable amounts of more valuable berry juices.
UIL.21.13829	Mango Juice Mazoon 200ml	Drink	RPC Derivatives	A039Y	Juice, mango	The group includes any type of juice made from Mango.
UIL.21.13827	Natural Mango Asafwa 200ml	Drink	RPC Derivatives	A039Y	Juice, mango	The group includes any type of juice made from Mango.
UIL.21.14142	Fruit Nectar (Lacnor)	Drink	Composites	A03BL	Mixed fruit nectars	The group includes any type of fruit nectars made from mixed fruit.
UIL.21.14132	Al Rabie Fruit Cocktail Nectar	Drink	Composites	A03BL	Mixed fruit nectars	The group includes any type of fruit nectars made from mixed fruit.
UIL.21.13851	Vimto	Drink	Composites	A03FQ	Cola-type drinks	The group includes any type of soft drinks with cola flavour, usually sweetened and carbonated.
UIL.21.14140	Sun Top Orange Fruit Drink	Drink	RPC Derivatives	A03AM	Juice, orange	The group includes any type of juice made from Orange.
UIL.21.14134	Al Marai Mango Juice	Drink	RPC Derivatives	A039Y	Juice, mango	The group includes any type of juice made from Mango.
UIL.21.13824	Mixed Fruit Juice (Mazoon)	Drink	RPC Derivatives	A03AP	Juice, mixed fruit	The group includes any type of Juice from mixed fruit.
UIL.21.14143	Fruit Drink (Orange) , Rani	Drink	RPC Derivatives	A03AM	Juice, orange	The group includes any type of juice made from Orange.
UIL.21.14139	A'safwah Mixed Fruit Juice	Drink	RPC Derivatives	A03AP	Juice, mixed fruit	The group includes any type of Juice from mixed fruit.
UIL.21.14141	Orange Drink (Top Fruit)	Drink	RPC Derivatives	A03AM	Juice, orange	The group includes any type of juice made from Orange.
UIL.21.14136	Nada Mango Juice	Drink	RPC Derivatives	A039Y	Juice, mango	The group includes any type of juice made from Mango.
UIL.21.14137	Nada Orange Juice	Drink	RPC Derivatives	A03AM	Juice, orange	The group includes any type of juice made from Orange.
UIL.21.13821	Asafwa Natural Orange 200 ml	Drink	RPC Derivatives	A03AM	Juice, orange	The group includes any type of juice made from Orange.
UIL.21.13822	Almarai Orange Juice 100% 300 ml	Drink	RPC Derivatives	A03AM#F10.A06HR	Juice, orange, QUALITATIVE-INFO = Integral /not refined	The group includes any type of juice made from Orange.

Sample ID (from lab)	Name of Product & Brand	Food Item	FOOD TYPE FE2	FoodEx2 Code	FoodEx2 Description	Description
UIL.21.13825	Top fruit Fruit Cocktail 200ml	Drink	RPC Derivatives	A03AP	Juice, mixed fruit	The group includes any type of Juice from mixed fruit.
UIL.21.14133	Al Rawabi Apple Juice	Drink	RPC Derivatives	A039M	Juice, apple	The group includes any type of juice made from Apple.
UIL.21.13826	Orange No Add Sugar (Top Fruit)	Drink	RPC Derivatives	A03AM#F10.A077K	Juice, orange, QUALITATIVE-INFO = Without added sugar	The group includes any type of juice made from Orange.
UIL.21.13823	Alrawabi Orange 1005juice	Drink	RPC Derivatives	A03AM#F10.A077K	Juice, orange, QUALITATIVE-INFO = Without added sugar	The group includes any type of juice made from Orange.
UIL.21.13450	Galaxy Smooth Milk	Drink	RPC - Raw Primary Commodities	A02LT#F10.A0CJQ	Milk, QUALITATIVE-INFO = Soft	The group includes Milk, i.e. a white nutritious liquid secreted by mammals and used as food source by human beings.
UIL.21.14569	Milk Powder (Majan)	Food	RPC Derivatives	A02PJ	Milk powder	he group includes any type of Milk powder.
UIL.21.14570	Milk Powder (Almudhish)	Food	RPC Derivatives	A02PJ	Milk powder	he group includes any type of Milk powder.
UIL.21.13552	Strwaberry Milk (Asafwah)	Drink	Composites	A02MP#F04.A06TV	Flavoured milks, INGREDIENT = Strawberry flavour	The group includes any type of Flavoured milk, i.e. milk containing sweetening and flavouring ingredients
UIL.21.13553	Strawberry flavored milk	Drink	Composites	A02MP#F04.A06TV	Flavoured milks, INGREDIENT = Strawberry flavour	The group includes any type of Flavoured milk, i.e. milk containing sweetening and flavouring ingredients
UIL.21.13554	Strawberry Fresh Milk (Alrawabi)	Drink	Composites	A02MP#F04.A06TV$F10.A166Y	Flavoured milks, INGREDIENT = Strawberry flavour, QUALITATIVE-INFO = Fresh	The group includes any type of Flavoured milk, i.e. milk containing sweetening and flavouring ingredients
UIL.21.14171	Strawberry Flavored Milk (Lacnor)	Drink	Composites	A02MP#F04.A06TV	Flavoured milks, INGREDIENT = Strawberry flavour	The group includes any type of Flavoured milk, i.e. milk containing sweetening and flavouring ingredients
UIL.21.14170	Strawberry Milk (Nada)	Drink	Composites	A02MP#F04.A06TV	Flavoured milks, INGREDIENT = Strawberry flavour	The group includes any type of Flavoured milk, i.e. milk containing sweetening and flavouring ingredients
UIL.21.14129	Evaporated Milk (Rainbow)	Drink	RPC Derivatives	A02PG	Condensed milk (sometimes with added sugars)	The group includes any type of Condensed milk, i.e. milk with reduced water content and often added sugars (as minor ingredients).
UIL.21.14130	Tea Milk (Almudhish)	Drink	Composites	A02MP#F04.A03LC	Flavoured milks, INGREDIENT = Fermented tea infusion	The group includes any type of Flavoured milk, i.e. milk containing sweetening and flavouring ingredients
UIL.21.13551	Mazoon Fresh Milk Strawberry Flavour	Drink	Composites	A02MP#F04.A06TV$F10.A166Y	Flavoured milks, INGREDIENT = Strawberry flavour, QUALITATIVE-INFO = Fresh	The group includes any type of Flavoured milk, i.e. milk containing sweetening and flavouring ingredients

Sample ID (from lab)	Name of Product & Brand	Food Item	FOOD TYPE FE2	FoodEx2 Code	FoodEx2 Description	Description
UIL.21.13849	Power Horse Energy	Drink	Composites	A03GA	Energy drinks	The group includes any type of Energy drinks, non-alcoholic functional beverages usually containing caffeine and other ingredients such as vitamins and taurine.
UIL.21.14173	Energy drink (Red bull)	Drink	Composites	A03GA	Energy drinks	The group includes any type of Energy drinks, non-alcoholic functional beverages usually containing caffeine and other ingredients such as vitamins and taurine.
UIL.21.14178	Vitaene Extra (Pokka)	Drink	Composites	A03GA#F10.A077L	Energy drinks, QUALITATIVE-INFO = Sugar free	The group includes any type of Energy drinks, non-alcoholic functional beverages usually containing caffeine and other ingredients such as vitamins and taurine.
UIL.21.14176	Mountain Dew	Drink	Composites	A03FJ#F10.A0CQG	Soft drink, orange flavour, QUALITATIVE-INFO = With added caffeine	The group includes any type of soft drink with orange flavour.
UIL.21.14175	Pepsi	Drink	Composites	A03FQ	Cola-type drinks	The group includes any type of soft drinks with cola flavour, usually sweetened and carbonated. Though by default cola beverages are with caffeine, also the variants without caffeine are included.
UIL.21.14174	Coca cola	Drink	Composites	A03FS	Cola mix, flavoured cola	The group includes any type of Cola Mix flavoured cola, i.e. a soft drink made by mixing a fruit-flavored syrup into cola, such as Cherry cola.
UIL.21.14172	Lipton Peach Ice tea	Drink	Composites	A16GP#F04.A06SB	Tea extract (liquid), INGREDIENT = Peach flavour	The group includes any type of tea extract in liquid form. The part consumed/analysed is by default the whole marketed unit or a homogeneous representative portion.
UIL.21.14177	Malt beverage (Barbican)	Drink	Composites	A03EX#F04.A06QL	Soft drink, flavoured, no fruit, INGREDIENT = Malt flavour	The group includes any type of flavoured soft drink, not containing fruit pulp or juice.

Table S2: Classification of food products according the NOVA system.

Sample ID (from lab)	Name of Product & Brand	Nutrient Profile Group	NOVA Groups	NOVA Classification
UIL.21.14412	Pure Honey / Capilano	0	2	Processed culinary ingredients
UIL.21.14414	Natural Honey / Alshifa	0	2	Processed culinary ingredients
UIL.21.14410	Custard Powder / Alalali	1	4	Ultra-processed food and drink products
UIL.21.14391	Nutella Hazelnut Spread with Cocoa	1	4	Ultra-processed food and drink products
UIL.21.14396	Daily Fresh/ Custard Powder	1	4	Ultra-processed food and drink products
UIL.21.13435	Kinder Joy	1	4	Ultra-processed food and drink products
UIL.21.13437	Kitkat	1	4	Ultra-processed food and drink products
UIL.21.13438	Snickers Minis	1	4	Ultra-processed food and drink products
UIL.21.13439	Bounty Minis	1	4	Ultra-processed food and drink products
UIL.21.13440	Mars Minis	1	4	Ultra-processed food and drink products
UIL.21.13441	Extra Peppermint	1	4	Ultra-processed food and drink products
UIL.21.13442	Batook Specialmint Chewing Gum	1	4	Ultra-processed food and drink products
UIL.21.13443	Wrigleys Doublemint Chewing Gum	1	4	Ultra-processed food and drink products
UIL.21.13444	Mentos Sugarfree Chewing Gum	1	4	Ultra-processed food and drink products
UIL.21.13550	Danet Cream Caramel	1	4	Ultra-processed food and drink products
UIL.21.13454	Twix Minis	1	4	Ultra-processed food and drink products
UIL.21.14409	Custard Powder / Tiara	1	4	Ultra-processed food and drink products
UIL.21.13560	Cream Caramel Almaraei	1	4	Ultra-processed food and drink products
UIL.21.13463	Cheetos / Flamin Hot	2	4	Ultra-processed food and drink products
UIL.21.14383	Lay's (Salt)	2	4	Ultra-processed food and drink products
UIL.21.14384	Al Mudhish Puffed Corn Cheese Balls	2	4	Ultra-processed food and drink products
UIL.21.14385	Potato Chips Ready Salted	2	4	Ultra-processed food and drink products
UIL.21.14386	Chips Oman	2	4	Ultra-processed food and drink products
UIL.21.14388	Shai (Mix Nut)	2	3	Processed foods
UIL.21.14389	Popcorn Cheese / American Garden	2	4	Ultra-processed food and drink products
UIL.21.14390	Mani (Mixed Nuts)	2	3	Processed foods
UIL.21.13433	Alrifai (Mixed Nuts)	2	3	Processed foods
UIL.21.13431	American Garden Popcorn Butter	2	4	Ultra-processed food and drink products
UIL.21.13432	Best Salted Mixed Nuts	2	3	Processed foods
UIL.21.13458	Nabil Cracker Salted Biscuits	2	4	Ultra-processed food and drink products
UIL.21.13462	Pringles Original	2	4	Ultra-processed food and drink products
UIL.21.17408	Cheese Balls (Mr Krispy)	2	4	Ultra-processed food and drink products
UIL.21.14418	Cheese Balls (Pofak Oman)	2	4	Ultra-processed food and drink products
UIL.21.14551	Cheese Pastry (Dhahabi)	2	4	Ultra-processed food and drink products
UIL.21.14560	Sohar Chips	2	4	Ultra-processed food and drink products

Sample ID (from lab)	Name of Product & Brand	Nutrient Profile Group	NOVA Groups	NOVA Classification
UIL.21.13833	Vanilla Ice cream (Igloo)	4	4	Ultra-processed food and drink products
UIL.21.13832	Vanilla Ice cream (Mazoon)	4	4	Ultra-processed food and drink products
UIL.21.14387	Kelloggs Special (Cereal)	5	4	Ultra-processed food and drink products
UIL.21.13298	Nestle Gold Corn Flakes	5	4	Ultra-processed food and drink products
UIL.21.13299	Nestle Chocapic	5	4	Ultra-processed food and drink products
UIL.21.13300	Kelloggs Coco Pops	5	4	Ultra-processed food and drink products
UIL.21.13448	Tiffany Glucose (Milk and Honey Biscuits)	6	4	Ultra-processed food and drink products
UIL.21.14574	7 Days Mini Croissant	6	4	Ultra-processed food and drink products
UIL.21.13445	Nabil Glucose	6	4	Ultra-processed food and drink products
UIL.21.13457	Lotus Biscoff	6	4	Ultra-processed food and drink products
UIL.21.13459	Original Oreo	6	4	Ultra-processed food and drink products
UIL.21.13460	Americana Premium Butter Cookies	6	4	Ultra-processed food and drink products
UIL.21.13461	Danish Butter Cookies	6	4	Ultra-processed food and drink products
UIL.21.13446	Nabil Cream Wafers (Chocolate Flavour)	6	4	Ultra-processed food and drink products
UIL.21.13447	Nabil Nice (Sugar Sprinkled Coconut Biscuits)	6	4	Ultra-processed food and drink products
UIL.21.14567	Switz Mini Cupcake Vanilla	6	4	Ultra-processed food and drink products
UIL.21.14566	Atyab Twin Cake Vanilla	6	4	Ultra-processed food and drink products
UIL.21.13449	Tiffany Everyday Nice (Sugar Sprinkled Coconut Biscuits)	6	4	Ultra-processed food and drink products
UIL.21.13451	Britannia Chocolate Flavoured Premium Crème Wafer	6	4	Ultra-processed food and drink products
UIL.21.13452	Nutro Chocolate Flavoured Cream Wafers	6	4	Ultra-processed food and drink products
UIL.21.13453	Tiffany Crunch And Cream	6	4	Ultra-processed food and drink products
UIL.21.17407	Donut (Switz)	6	4	Ultra-processed food and drink products
UIL.21.17406	Cheese Puff (Lusine)	6	4	Ultra-processed food and drink products
UIL.21.17405	Apple Puff (Lusine)	6	4	Ultra-processed food and drink products
UIL.21.14552	Apple Pastry	6	4	Ultra-processed food and drink products
UIL.21.14568	Chocolate Jumbo Croissant	6	4	Ultra-processed food and drink products
UIL.21.17531	Croissant (Dhahabi)	6	4	Ultra-processed food and drink products
UIL.21.13830	Nestle Cream	7	4	Ultra-processed food and drink products
UIL.21.13592	Fresh Labnah, Full Fat Al-Marai	7	1	Unprocessed or minimally processed foods
UIL.21.13593	Labneh Turkish Reciepe: Puck	7	4	Ultra-processed food and drink products
UIL.21.13594	Fresh Labnah: Full Fat Mazoon	7	1	Unprocessed or minimally processed foods
UIL.21.13558	Fresh Yougurt, Full Fat: Al-Marai	7	1	Unprocessed or minimally processed foods
UIL.21.13556	Fresh Yougurt, Full Fat: Al-Safwah	7	1	Unprocessed or minimally processed foods
UIL.21.13559	Youghurt; Full Cream Alrawabi	7	1	Unprocessed or minimally processed foods
UIL.21.13557	Yoghurt: Full Cream Unikai	7	1	Unprocessed or minimally processed foods
UIL.21.13546	Alrawabi Up: Laban Drink	7	1	Unprocessed or minimally processed foods
UIL.21.13544	Almarai Fresh Laban Full Fat	7	1	Unprocessed or minimally processed foods

Sample ID (from lab)	Name of Product & Brand	Nutrient Profile Group	NOVA Groups	NOVA Classification
UIL.21.13548	Almarai Strawberry Laban	7	4	Ultra-processed food and drink products
UIL.21.13545	Almarai Laban Up	7	1	Unprocessed or minimally processed foods
UIL.21.13542	Mazoon Fresh Laban Full Fat	7	1	Unprocessed or minimally processed foods
UIL.21.13547	Mazoon Strawberry Laban	7	4	Ultra-processed food and drink products
UIL.21.13540	Mazoon Laban Up	7	1	Unprocessed or minimally processed foods
UIL.21.13555	Mazoon Fresh Yoghurt Full Fat	7	1	Unprocessed or minimally processed foods
UIL.21.14180	Mazoon Sambharam Spicy Laban Drink	7	4	Ultra-processed food and drink products
UIL.21.17409	Activia Full Fat Plain Yoghurt	7	1	Unprocessed or minimally processed foods
UIL.21.17404	Balade Farms Ayran Original Laban	7	1	Unprocessed or minimally processed foods
UIL.21.17403	Activia Full Fat Fresh Laban	7	1	Unprocessed or minimally processed foods
UIL.21.14553	Cream (Puck)	7	4	Ultra-processed food and drink products
UIL.21.13549	Laban Drink (Unikai)	7	1	Unprocessed or minimally processed foods
UIL.21.13541	Laban Up (Alsafwa)	7	1	Unprocessed or minimally processed foods
UIL.21.13543	Laban Fresh Full Creeam (Asafwah)	7	1	Unprocessed or minimally processed foods
UIL.21.13539	Fresh Laban (Alrawabi)	7	1	Unprocessed or minimally processed foods
UIL.21.14131	Cream (Luna)	7	4	Ultra-processed food and drink products
UIL.21.14179	Laban Drinks (Alkhamayil)	7	1	Unprocessed or minimally processed foods
UIL.21.13836	Triangle Cheese Abu Alwald	9	4	Ultra-processed food and drink products
UIL.21.13838	Cheese Triangles Puck	9	4	Ultra-processed food and drink products
UIL.21.13837	Cheese Triangles Al Maraai	9	4	Ultra-processed food and drink products
UIL.21.13834	Haloumi Cheese Al-Marai	9	4	Ultra-processed food and drink products
UIL.21.13835	Haloumi Pinar	9	4	Ultra-processed food and drink products
UIL.21.13848	Kraft Cheddar Cheese Spread Original	9	4	Ultra-processed food and drink products
UIL.21.13843	Mazoon Spread Cheese	9	4	Ultra-processed food and drink products
UIL.21.13842	Mazoon Spread Cheese Cheddar	9	4	Ultra-processed food and drink products
UIL.21.13847	Puck Cream Cheese	9	4	Ultra-processed food and drink products
UIL.21.13846	Puck Cream Cheese Cheddar	9	4	Ultra-processed food and drink products
UIL.21.13845	Almarai Cream Cheese	9	4	Ultra-processed food and drink products
UIL.21.13844	Almarai Cream Cheese Cheddar	9	4	Ultra-processed food and drink products
UIL.21.13839	Kraft Slice Cheese Original	9	4	Ultra-processed food and drink products
UIL.21.13841	Puck Slice Cheese Original	9	4	Ultra-processed food and drink products
UIL.21.13840	Almarai Burger Slice Cheese	9	4	Ultra-processed food and drink products
UIL.21.14554	Pinar Processed Cream Cheese Spread	9	4	Ultra-processed food and drink products
UIL.21.14571	Chedder Cheese (Kraft)	9	4	Ultra-processed food and drink products
UIL.21.14572	Chedder Cheese (Almarai)	9	4	Ultra-processed food and drink products
UIL.21.13455	Tiffany Whole Wheat Rusks	11	4	Ultra-processed food and drink products
UIL.21.13456	Britannia Toastea Wheat Rusk	11	4	Ultra-processed food and drink products

Sample ID (from lab)	Name of Product & Brand	Nutrient Profile Group	NOVA Groups	NOVA Classification
UIL.21.13429	Indomie Fried Noodles	12	1	Unprocessed or minimally processed foods
UIL.21.14195	Sadia Chicken Nuggets Crispy	14	4	Ultra-processed food and drink products
UIL.21.14198	Sadia Chicken Burger	14	4	Ultra-processed food and drink products
UIL.21.14199	Beef Burger Sadia	14	4	Ultra-processed food and drink products
UIL.21.14203	Al-Kabeer Jumbo Hotdogs	14	4	Ultra-processed food and drink products
UIL.21.14202	Al-Islami Ground Beef	14	4	Ultra-processed food and drink products
UIL.21.14205	Al-Safa Chicken Franks	14	4	Ultra-processed food and drink products
UIL.21.14206	Sadia Chicken Franks	14	4	Ultra-processed food and drink products
UIL.21.14204	Americana Beef Hotdog	14	4	Ultra-processed food and drink products
UIL.21.14196	Al-Safa Chicken Burger	14	4	Ultra-processed food and drink products
UIL.21.14194	Al-Safa Breaded Chicken Finger	14	4	Ultra-processed food and drink products
UIL.21.14573	Family Corned Beef	14	4	Ultra-processed food and drink products
UIL.21.14200	Beef Burger (Taybat)	14	4	Ultra-processed food and drink products
UIL.21.14201	Corned Beef (Taybat)	14	4	Ultra-processed food and drink products
UIL.21.13434	White Tuna Meat In Brine	15	3	Processed foods
UIL.21.13291	Skipjack Tuna Alalali In Sun Flower Oil	15	3	Processed foods
UIL.21.13292	Tuna Alalali In Water	15	3	Processed foods
UIL.21.13293	White Tuna Meat In Sun Flower Oil Delmonte	15	3	Processed foods
UIL.21.13294	Skipjack Tuna California Garden In Sun Flower Oil	15	3	Processed foods
UIL.21.13295	Light Chunks Tuna California Garden In Water	15	3	Processed foods
UIL.21.14397	Diamond Strawberry Jam	17	3	Processed foods
UIL.21.14556	Luna Baked Beans In Tomato Sauce	17	3	Processed foods
UIL.21.14558	Lunafoul Medames Extra Grade Fava Beans	17	3	Processed foods
UIL.21.14555	LA MING Beans In Tomato Sauce	17	3	Processed foods
UIL.21.13276	Green Giant Sweet Corn	17	3	Processed foods
UIL.21.13285	Green Peas Luna	17	3	Processed foods
UIL.21.13286	Chick Peas Almazraa	17	3	Processed foods
UIL.21.13287	Processed Peas Almazraa	17	3	Processed foods
UIL.21.13288	Sweet Corn Libbys	17	3	Processed foods
UIL.21.13289	Boildchick Peas Mara	17	3	Processed foods
UIL.21.13290	Sweet Corn American Garden	17	3	Processed foods
UIL.21.13277	Plain Medammes California Garden	17	3	Processed foods
UIL.21.13278	Baked Beans California Garden	17	3	Processed foods
UIL.21.13279	Processed Peas California Garden	17	3	Processed foods
UIL.21.13280	Sweet Corn California Garden	17	3	Processed foods
UIL.21.13281	Chick Peas California Garden	17	3	Processed foods
UIL.21.13282	Baked Beans Almazraa	17	3	Processed foods

Sample ID (from lab)	Name of Product & Brand	Nutrient Profile Group	NOVA Groups	NOVA Classification
UIL.21.13283	Foul Medamas Almazraa	17	3	Processed foods
UIL.21.13284	Chick Peas Luna	17	3	Processed foods
UIL.21.14399	Tomato Paste / Almudhish	17	3	Processed foods
UIL.21.14400	Tomato Paste / Jumbo	17	3	Processed foods
UIL.21.14403	Mango Pickles In Oil / Ahmed	17	3	Processed foods
UIL.21.14404	Tomato Paste / Alalali	17	3	Processed foods
UIL.21.14557	Foul Medammas (American Garden)	17	3	Processed foods
UIL.21.14559	Peas Processed	17	3	Processed foods
UIL.21.14405	Mango Thokku Pickle In Oil / Priya	17	3	Processed foods
UIL.21.14406	Mango Pickle / Aeroplane	17	3	Processed foods
UIL.21.14408	Tomato Ketchup / Hayat	18	4	Ultra-processed food and drink products
UIL.21.14411	Tomato Ketchup/ Jumbo	18	4	Ultra-processed food and drink products
UIL.21.14413	Tomato Ketchup / Heinz	18	4	Ultra-processed food and drink products
UIL.21.14415	Classic Mayonnaise / Mazola	18	4	Ultra-processed food and drink products
UIL.21.14416	Creamy Classic Mayyonaise / Heinz	18	4	Ultra-processed food and drink products
UIL.21.14417	Hot Sause / American Garden	18	4	Ultra-processed food and drink products
UIL.21.14563	Knor Vegetable Stock Cubes	18	4	Ultra-processed food and drink products
UIL.21.14392	Delicio French Dressing	18	4	Ultra-processed food and drink products
UIL.21.14393	Delicio 1000 Island	18	4	Ultra-processed food and drink products
UIL.21.14394	American Garden BBQ Sauce Original	18	4	Ultra-processed food and drink products
UIL.21.14395	Heinz Classic BBQ Smokey And Rich	18	4	Ultra-processed food and drink products
UIL.21.14565	Maggi Chicken Stock Less Salt	18	4	Ultra-processed food and drink products
UIL.21.13296	Hayat Hot Sauce	18	4	Ultra-processed food and drink products
UIL.21.13297	Jumbo Hot Sauce	18	4	Ultra-processed food and drink products
UIL.21.13430	Maggi Chicken Stock	18	4	Ultra-processed food and drink products
UIL.21.14564	Maggi Beef Flavour	18	4	Ultra-processed food and drink products
UIL.21.14562	Knour Beef Flavoured Stock	18	4	Ultra-processed food and drink products
UIL.21.14561	Knour CHICKEN Stock	18	4	Ultra-processed food and drink products
UIL.21.14398	Soy Sauce Classic / American Garden	18	4	Ultra-processed food and drink products
UIL.21.14402	Hot Sauce / Excellnce	18	4	Ultra-processed food and drink products
UIL.21.14407	Pizza Sauce Olive& Mushrooms / Alalali	18	4	Ultra-processed food and drink products
UIL.21.14401	Tabasco Hot Sauce	18	4	Ultra-processed food and drink products
UIL.21.13850	Al Deyafa Orang Flavour (Powder)	3a	4	Ultra-processed food and drink products
UIL.21.13851	Vimto	3a	4	Ultra-processed food and drink products
UIL.21.13828	Al Deyafa Fruit Cordial	3a	4	Ultra-processed food and drink products
UIL.21.14133	Al Rawabi Apple Juice	3a	4	Ultra-processed food and drink products
UIL.21.14134	Al Marai Mango Juice	3a	4	Ultra-processed food and drink products

Sample ID (from lab)	Name of Product & Brand	Nutrient Profile Group	NOVA Groups	NOVA Classification
UIL.21.14132	Al Rabie Fruit Cocktail Nectar	3a	4	Ultra-processed food and drink products
UIL.21.14135	Almarai Graps And Berries (Farms Select)	3a	4	Ultra-processed food and drink products
UIL.21.14137	Nada Orange Juice	3a	4	Ultra-processed food and drink products
UIL.21.14136	Nada Mango Juice	3a	4	Ultra-processed food and drink products
UIL.21.14138	Al Rawabi Red Grape Juice	3a	4	Ultra-processed food and drink products
UIL.21.14139	A'safwah Mixed Fruit Juice	3a	4	Ultra-processed food and drink products
UIL.21.13829	Mango Juice Mazoon 200ml	3a	4	Ultra-processed food and drink products
UIL.21.13827	Natural Mango Asafwa 200ml	3a	4	Ultra-processed food and drink products
UIL.21.13823	Alrawabi Orange 1005 juice	3a	4	Ultra-processed food and drink products
UIL.21.13825	Topfruit Fruit Cocktail 200ml	3a	4	Ultra-processed food and drink products
UIL.21.13822	Almarai Orange Juice 100% 300 ml	3a	4	Ultra-processed food and drink products
UIL.21.13821	Asafwa Natural Orange 200 ml	3a	4	Ultra-processed food and drink products
UIL.21.14140	Sun Top Orange Fruit Drink	3a	4	Ultra-processed food and drink products
UIL.21.17402	Tang (Orange) (Powder)	3a	4	Ultra-processed food and drink products
UIL.21.13824	Mixed Fruit Juice (Mazoon)	3a	4	Ultra-processed food and drink products
UIL.21.14143	Fruit Drink (Orange) Rani	3a	4	Ultra-processed food and drink products
UIL.21.14141	Orange Drink (Top Fruit)	3a	4	Ultra-processed food and drink products
UIL.21.14142	Fruit Nectar (Lacnor)	3a	4	Ultra-processed food and drink products
UIL.21.13826	Orange No Add Sugar (Top Fruit)	3a	4	Ultra-processed food and drink products
UIL.21.13551	Mazoon Fresh Milk Strawberry Flavour	3c	4	Ultra-processed food and drink products
UIL.21.13450	Galaxy Smooth Milk	3c	4	Ultra-processed food and drink products
UIL.21.13552	Strwaberry Milk (Asafwah)	3c	4	Ultra-processed food and drink products
UIL.21.13554	Strwberry Fresh Milk (Alrawabi)	3c	4	Ultra-processed food and drink products
UIL.21.13553	Strawberry Flavored Milk	3c	4	Ultra-processed food and drink products
UIL.21.14570	Milk Powder (Almudhish)	3c	1	Unprocessed or minimally processed foods
UIL.21.14569	Milk Powder (Majan)	3c	1	Unprocessed or minimally processed foods
UIL.21.14129	Evaporated Milk (Rainbow)	3c	4	Ultra-processed food and drink products
UIL.21.14130	Tea Milk (Almudhish)	3c	4	Ultra-processed food and drink products
UIL.21.14170	Strawberry Milk (Nada)	3c	4	Ultra-processed food and drink products
UIL.21.14171	Strawberry Flavored Milk (Lacnor)	3c	4	Ultra-processed food and drink products
UIL.21.13849	Power Horse Energy	3d	4	Ultra-processed food and drink products
UIL.21.14173	Energy Drink (Red Bull)	3d	4	Ultra-processed food and drink products
UIL.21.14172	Lipton Peach Ice Tea	3e	1	Unprocessed or minimally processed foods
UIL.21.14177	Malt Beverage (Barbican)	3e	4	Ultra-processed food and drink products
UIL.21.14178	Vitaene Extra (Pokka)	3e	4	Ultra-processed food and drink products
UIL.21.14176	Mountain Dew	3e	4	Ultra-processed food and drink products
UIL.21.14175	Pepsi	3e	4	Ultra-processed food and drink products
UIL.21.14174	Coca Cola	3e	4	Ultra-processed food and drink products

Table S3: Classification of food items according the EMR nutrient profile.

Sample ID (from lab)	Name of Product & Brand	Nutrient Profile Group	Marketing Permitted/not permitted	Food category	Amounts for 100 g/ml of the product				
					Total fat (g)	Total Sugars (g)	Energy (kcal)	Sat fat (g)	Salt (g)
UIL.21.14414	Natural Honey / Alshifa	0	not include in NP	NO NP Class	0.00	80.30	334.56	0.00	0.00
UIL.21.14412	Pure honey / Capilano	0	not include in NP	NO NP Class	0.00	81.00	327.76	0.00	0.00
UIL.21.13442	Batook Specialmint Chewing Gum	1	not permitted	CHOCOLATE AND SUGAR CONFECTIONARY; ENERGY BARS; SWEET TOPPINGS AND DESSERTS	0.40	2.00	370.00	0.08	0.00
UIL.21.13439	Bounty Minis	1	not permitted	CHOCOLATE AND SUGAR CONFECTIONARY; ENERGY BARS; SWEET TOPPINGS AND DESSERTS	14.34	15.02	438.02	8.38	0.82
UIL.21.13560	Cream Caramel Almaraei	1	not permitted	CHOCOLATE AND SUGAR CONFECTIONARY; ENERGY BARS; SWEET TOPPINGS AND DESSERTS	2.23	20.92	124.03	1.66	0.15
UIL.21.14410	Custard Powder / Alalali	1	not permitted	CHOCOLATE AND SUGAR CONFECTIONARY; ENERGY BARS; SWEET TOPPINGS AND DESSERTS	0.01	0.10	363.13	0.00	0.21
UIL.21.14409	Custard Powder/Tiara	1	not permitted	CHOCOLATE AND SUGAR CONFECTIONARY; ENERGY BARS; SWEET TOPPINGS AND DESSERTS	0.23	0.21	355.15	0.14	0.00
UIL.21.14396	Daily Fresh/ Custard Powder	1	not permitted	CHOCOLATE AND SUGAR CONFECTIONARY; ENERGY BARS; SWEET TOPPINGS AND DESSERTS	0.64	3.07	340.00	0.21	1.78
UIL.21.13550	Danet Cream Caramel	1	not permitted	CHOCOLATE AND SUGAR CONFECTIONARY; ENERGY BARS; SWEET TOPPINGS AND DESSERTS	2.21	17.80	115.05	1.59	0.16
UIL.21.13441	Extra Peppermint	1	not permitted	CHOCOLATE AND SUGAR CONFECTIONARY; ENERGY BARS; SWEET TOPPINGS AND DESSERTS	0.53	52.63	510.00	0.11	0.00
UIL.21.13435	Kinder Joy	1	not permitted	CHOCOLATE AND SUGAR CONFECTIONARY; ENERGY BARS; SWEET TOPPINGS AND DESSERTS	32.22	49.62	545.78	15.15	0.49
UIL.21.13437	Kitkat	1	not permitted	CHOCOLATE AND SUGAR CONFECTIONARY; ENERGY BARS; SWEET TOPPINGS AND DESSERTS	25.29	45.37	515.01	13.76	0.19
UIL.21.13440	Mars Minis	1	not permitted	CHOCOLATE AND SUGAR CONFECTIONARY; ENERGY BARS; SWEET TOPPINGS AND DESSERTS	17.92	57.77	473.31	11.54	0.56
UIL.21.13444	Mentos Sugar free Chewing Gum	1	not permitted	CHOCOLATE AND SUGAR CONFECTIONARY; ENERGY BARS; SWEET TOPPINGS AND DESSERTS	0.57	32.00	382.29	0.11	0.00
UIL.21.14391	Nutella Hazelnut Spread With Cocoa	1	not permitted	CHOCOLATE AND SUGAR CONFECTIONARY; ENERGY BARS; SWEET TOPPINGS AND DESSERTS	31.20	57.94	546.32	10.53	0.11
UIL.21.13438	Snickers Minis	1	not permitted	CHOCOLATE AND SUGAR CONFECTIONARY; ENERGY BARS; SWEET TOPPINGS AND DESSERTS	26.80	52.53	509.20	6.73	0.67
UIL.21.13454	Twix Minis	1	not permitted	CHOCOLATE AND SUGAR CONFECTIONARY; ENERGY BARS; SWEET TOPPINGS AND DESSERTS	25.35	48.60	523.35	11.45	0.47
UIL.21.13443	Wrigleys Doublemint Chewing Gum	1	not permitted	CHOCOLATE AND SUGAR CONFECTIONARY; ENERGY BARS; SWEET TOPPINGS AND DESSERTS	0.38	81.54	372.69	0.08	0.00
UIL.21.14384	Al Mudhish Puffed Corn Cheese Balls	2	not permitted (high salt)	SAVOURY SNACKS	35.75	4.42	568.08	16.58	1.58
UIL.21.13433	Alrifai (Mixed Nuts)	2	not permitted (high salt)	SAVOURY SNACKS	32.43	7.18	537.42	4.64	0.61
UIL.21.13431	American Garden Popcorn Butter	2	not permitted (high salt)	SAVOURY SNACKS	24.42	0.30	471.21	10.91	2.50
UIL.21.13432	Best Salted Mixed Nuts	2	not permitted (high salt)	SAVOURY SNACKS	51.89	4.28	643.33	8.31	0.85
UIL.21.14418	Cheese Balls (Pofak Oman)	2	not permitted (high salt)	SAVOURY SNACKS	39.15	4.17	579.83	16.79	1.50
UIL.21.14551	Cheese pastry (Dhahabi)	2	not permitted (high salt)	SAVOURY SNACKS	18.54	3.85	378.54	4.74	0.78
UIL.21.13463	Cheetos / Flamin Hot	2	not permitted (high salt)	SAVOURY SNACKS	34.63	1.07	573.70	9.73	1.78
UIL.21.17408	Cheez Balls (Mr. Krispy)	2	not permitted (high salt)	SAVOURY SNACKS	34.33	7.47	583.67	7.67	3.92
UIL.21.14386	chips Oman	2	not permitted (high salt)	SAVOURY SNACKS	38.77	0.00	565.61	16.89	1.28
UIL.21.14383	Lay's (Salt)	2	not permitted (high salt)	SAVOURY SNACKS	35.50	0.00	564.36	16.07	1.51
UIL.21.14390	Mani (Mixed Nuts)	2	not permitted (high salt)	SAVOURY SNACKS	49.33	8.55	623.81	7.92	0.80
UIL.21.13458	Nabil Cracker Salted Biscuits	2	not permitted (high salt)	SAVOURY SNACKS	19.26	20.04	475.26	13.50	2.54

Sample ID (from lab)	Name of Product & Brand	Nutrient Profile Group	Marketing Permitted/not permitted	Food category	Amounts for 100 g/ml of the product				
					Total fat (g)	Total Sugars (g)	Energy (kcal)	Sat fat (g)	Salt (g)
UIL.21.14389	Popcorn Cheese / American Garden	2	not permitted (high salt)	SAVOURY SNACKS	34.21	0.00	528.27	15.67	2.51
UIL.21.14385	potato chips ready salted	2	not permitted (high salt)	SAVOURY SNACKS	36.11	0.00	561.96	15.75	1.36
UIL.21.13462	Pringles Original	2	not permitted (high salt)	SAVOURY SNACKS	33.49	1.05	551.77	3.53	1.15
UIL.21.14388	Shai (Mix Nut)	2	not permitted (high salt)	SAVOURY SNACKS	48.44	6.85	619.56	6.87	0.38
UIL.21.14560	Sohar Chips	2	not permitted (high salt)	SAVOURY SNACKS	32.36	0.00	547.20	14.31	2.41
UIL.21.13833	vanilla ice cream (Igloo)	4	not permitted	EDIBLE ICES	11.13	18.60	197.25	6.68	0.17
UIL.21.13832	vanilla ice cream (Mazoon)	4	not permitted	EDIBLE ICES	7.66	12.37	136.22	4.46	0.12
UIL.21.13300	KELLOGG'S Coco Pops	5	not permitted(high sugars)	BREAKFAST CEREALS	2.48	24.48	382.96	1.15	0.00
UIL.21.14387	Kellogg's special (cereal)	5	not permitted(high salt)	BREAKFAST CEREALS	2.21	13.92	384.56	0.59	1.77
UIL.21.13299	NESTLE Chocapic	5	not permitted(high sugars)	BREAKFAST CEREALS	4.79	24.95	406.39	1.50	0.26
UIL.21.13298	Nestle Gold Corn Flakes	5	permitted	BREAKFAST CEREALS	1.96	10.57	383.36	0.38	1.08
UIL.21.14574	7 days mini croissant	6	not permitted	CAKES, SWEET BISCUITS AND PASTRIES; OTHER SWEET BAKER'S WARES, AND DRY MIXES FOR MAKING	28.55	16.82	471.82	16.73	0.52
UIL.21.13460	Americana Premium Butter Cookies	6	not permitted	CAKES, SWEET BISCUITS AND PASTRIES; OTHER SWEET BAKER'S WARES, AND DRY MIXES FOR MAKING	20.27	29.40	489.15	9.16	0.29
UIL.21.14552	Apple pastry	6	not permitted	CAKES, SWEET BISCUITS AND PASTRIES; OTHER SWEET BAKER'S WARES, AND DRY MIXES FOR MAKING	15.74	16.32	360.86	8.37	0.03
UIL.21.17405	Apple puff (Lusine)	6	not permitted	CAKES, SWEET BISCUITS AND PASTRIES; OTHER SWEET BAKER'S WARES, AND DRY MIXES FOR MAKING	13.10	19.66	345.16	5.89	0.70
UIL.21.14566	Atyab Twin Cake Vanilla	6	not permitted	CAKES, SWEET BISCUITS AND PASTRIES; OTHER SWEET BAKER'S WARES, AND DRY MIXES FOR MAKING	18.43	27.82	413.99	7.17	0.56
UIL.21.13451	Britannia Chocolate Flavoured Premium Crème Wafer	6	not permitted	CAKES, SWEET BISCUITS AND PASTRIES; OTHER SWEET BAKER'S WARES, AND DRY MIXES FOR MAKING	25.38	33.81	513.88	15.75	0.63
UIL.21.17406	Cheese Puff (Lusine)	6	not permitted	CAKES, SWEET BISCUITS AND PASTRIES; OTHER SWEET BAKER'S WARES, AND DRY MIXES FOR MAKING	22.03	8.77	383.63	10.30	1.56
UIL.21.14568	Chocolate jumbo croissant	6	not permitted	CAKES, SWEET BISCUITS AND PASTRIES; OTHER SWEET BAKER'S WARES, AND DRY MIXES FOR MAKING	23.69	12.08	422.01	13.05	0.51
UIL.21.17531	Croissant (Dhahabi)	6	not permitted	CAKES, SWEET BISCUITS AND PASTRIES; OTHER SWEET BAKER'S WARES, AND DRY MIXES FOR MAKING	23.18	11.95	437.90	10.21	1.58
UIL.21.13461	Danish Butter Cookies	6	not permitted	CAKES, SWEET BISCUITS AND PASTRIES; OTHER SWEET BAKER'S WARES, AND DRY MIXES FOR MAKING	25.44	26.04	510.56	18.34	0.32
UIL.21.17407	Donut (Switz)	6	not permitted	CAKES, SWEET BISCUITS AND PASTRIES; OTHER SWEET BAKER'S WARES, AND DRY MIXES FOR MAKING	17.57	19.74	405.00	5.57	0.61
UIL.21.13457	Lotus Biscoff	6	not permitted	CAKES, SWEET BISCUITS AND PASTRIES; OTHER SWEET BAKER'S WARES, AND DRY MIXES FOR MAKING	19.36	36.41	491.67	13.59	1.05
UIL.21.13446	Nabil cream wafers (chocolate flavour)	6	not permitted	CAKES, SWEET BISCUITS AND PASTRIES; OTHER SWEET BAKER'S WARES, AND DRY MIXES FOR MAKING	21.29	20.63	495.05	12.74	0.37
UIL.21.13445	Nabil glucose	6	not permitted	CAKES, SWEET BISCUITS AND PASTRIES; OTHER SWEET BAKER'S WARES, AND DRY MIXES FOR MAKING	14.36	14.88	460.72	7.42	0.00
UIL.21.13447	Nabil Nice (sugar sprinkled coconut biscuits)	6	not permitted	CAKES, SWEET BISCUITS AND PASTRIES; OTHER SWEET BAKER'S WARES, AND DRY MIXES FOR MAKING	15.36	21.16	463.32	7.12	0.95
UIL.21.13452	Nutro Chocolate Flavoured Cream Wafers	6	not permitted	CAKES, SWEET BISCUITS AND PASTRIES; OTHER SWEET BAKER'S WARES, AND DRY MIXES FOR MAKING	30.20	36.17	535.93	26.80	0.52
UIL.21.13459	Original Oreo	6	not permitted	CAKES, SWEET BISCUITS AND PASTRIES; OTHER SWEET BAKER'S WARES, AND DRY MIXES FOR MAKING	19.47	42.11	482.84	8.79	1.48
UIL.21.14567	Switz Mini Cup Cake Vanilla	6	not permitted	CAKES, SWEET BISCUITS AND PASTRIES; OTHER SWEET BAKER'S WARES, AND DRY MIXES FOR MAKING	29.41	29.00	487.53	12.75	1.03

Sample ID (from lab)	Name of Product & Brand	Nutrient Profile Group	Marketing Permitted/not permitted	Food category	Amounts for 100 g/ml of the product				
					Total fat (g)	Total Sugars (g)	Energy (kcal)	Sat fat (g)	Salt (g)
UIL.21.13453	Tiffany Crunch And Cream	6	not permitted	CAKES, SWEET BISCUITS AND PASTRIES; OTHER SWEET BAKER'S WARES, AND DRY MIXES FOR MAKING	24.36	36.23	512.36	21.51	0.31
UIL.21.13449	Tiffany Everyday Nice (Sugar Sprinkled Coconut Biscuits)	6	not permitted	CAKES, SWEET BISCUITS AND PASTRIES; OTHER SWEET BAKER'S WARES, AND DRY MIXES FOR MAKING	20.32	25.48	493.87	9.16	0.90
UIL.21.13448	Tiffany Glucose (Milk and Honey Biscuits)	6	not permitted	CAKES, SWEET BISCUITS AND PASTRIES; OTHER SWEET BAKER'S WARES, AND DRY MIXES FOR MAKING	17.84	22.90	479.26	9.16	0.91
UIL.21.17403	Activia Full Fat Fresh Laban	7	permitted	YOGHURTS, SOUR MILK, CREAM AND OTHER SIMILAR FOODS	3.28	4.46	60.92	2.26	0.13
UIL.21.17409	Activia Full Fat Plain Yoghurt	7	permitted	YOGHURTS, SOUR MILK, CREAM AND OTHER SIMILAR FOODS	2.08	3.32	44.77	1.53	0.09
UIL.21.13544	Almarai Fresh Laban Full Fat	7	not permitted (high totalfat & high saturatedfat & high salt)	YOGHURTS, SOUR MILK, CREAM AND OTHER SIMILAR FOODS	3.64	4.09	62.42	2.57	0.14
UIL.21.13545	Almarai Laban Up	7	not permitted (high salt)	YOGHURTS, SOUR MILK, CREAM AND OTHER SIMILAR FOODS	1.15	3.99	38.21	0.81	0.80
UIL.21.13548	Almarai Strawberry Laban	7	not permitted (high salt & high sugar)	YOGHURTS, SOUR MILK, CREAM AND OTHER SIMILAR FOODS	1.36	15.29	87.91	0.91	0.14
UIL.21.13546	Alrawabi Up: Laban Drink	7	not permitted (high salt)	YOGHURTS, SOUR MILK, CREAM AND OTHER SIMILAR FOODS	1.79	3.04	37.63	1.20	0.00
UIL.21.17404	Balade Farms Ayran Original Laban	7	not permitted (high salt)	YOGHURTS, SOUR MILK, CREAM AND OTHER SIMILAR FOODS	1.27	0.00	31.63	1.02	0.95
UIL.21.14131	Cream (Luna)	7	not permitted (high saturatedfat & high salt)	YOGHURTS, SOUR MILK, CREAM AND OTHER SIMILAR FOODS	21.71	3.10	219.39	16.71	0.00
UIL.21.14553	Cream (Puck)	7	not permitted (high total fat & high saturated fat & high salt)	YOGHURTS, SOUR MILK, CREAM AND OTHER SIMILAR FOODS	24.51	0.00	242.71	11.62	0.01
UIL.21.13539	Fresh Laban (Alrawabi)	7	not permitted (high totalfat & high saturatedfat & high salt)	YOGHURTS, SOUR MILK, CREAM AND OTHER SIMILAR FOODS	3.27	3.97	61.03	2.29	0.00
UIL.21.13592	Fresh Labneh, Full Fat Al-Marai	7	not permitted (high totalfat & high saturatedfat & high salt)	YOGHURTS, SOUR MILK, CREAM AND OTHER SIMILAR FOODS	12.85	7.33	174.75	8.82	0.57
UIL.21.13594	Fresh Labneh: Full Fat Mazoon	7	not permitted (high totalfat & high saturatedfat & high salt)	YOGHURTS, SOUR MILK, CREAM AND OTHER SIMILAR FOODS	11.13	6.63	154.97	7.82	0.54
UIL.21.13558	Fresh Yougurt Full Fat: Al-Marai	7	not permitted (high totalfat & high saturatedfat & high salt)	YOGHURTS, SOUR MILK, CREAM AND OTHER SIMILAR FOODS	3.61	5.62	72.44	2.57	0.18
UIL.21.13556	Fresh Yougurt Full Fat: Al-Safwah	7	not permitted (high totalfat)	YOGHURTS, SOUR MILK, CREAM AND OTHER SIMILAR FOODS	3.23	4.78	66.59	2.01	0.18
UIL.21.13549	Laban Drink (Unikai)	7	not permitted (high salt)	YOGHURTS, SOUR MILK, CREAM AND OTHER SIMILAR FOODS	0.74	3.02	32.58	0.48	4.76
UIL.21.14179	Laban Drinks (Alkhamayil)	7	not permitted (high salt)	YOGHURTS, SOUR MILK, CREAM AND OTHER SIMILAR FOODS	0.58	0.89	29.86	0.37	0.00
UIL.21.13543	Laban Fresh Full Cream (Asafwah)	7	not permitted (high totalfat & high saturatedfat & high salt)	YOGHURTS, SOUR MILK, CREAM AND OTHER SIMILAR FOODS	3.31	4.07	63.43	2.37	0.17
UIL.21.13541	Laban Up (Alsafwa)	7	not permitted (high salt)	YOGHURTS, SOUR MILK, CREAM AND OTHER SIMILAR FOODS	1.55	3.89	40.19	1.04	0.87
UIL.21.13593	Labneh Turkish Recipe: Puck	7	not permitted (high totalfat & high saturatedfat & high salt)	YOGHURTS, SOUR MILK, CREAM AND OTHER SIMILAR FOODS	14.21	6.43	189.21	9.82	0.79
UIL.21.13542	Mazoon Fresh Laban Full Fat	7	not permitted (high totalfat)	YOGHURTS, SOUR MILK, CREAM AND OTHER SIMILAR FOODS	3.30	4.48	64.62	2.22	0.13
UIL.21.13555	Mazoon Fresh Yoghurt Full Fat	7	not permitted (high totalfat & high saturatedfat & high salt)	YOGHURTS, SOUR MILK, CREAM AND OTHER SIMILAR FOODS	3.21	5.04	68.05	2.29	0.15
UIL.21.13540	Mazoon Laban Up	7	not permitted (high salt)	YOGHURTS, SOUR MILK, CREAM AND OTHER SIMILAR FOODS	0.89	3.70	30.49	0.60	0.88
UIL.21.14180	Mazoon Sambharam Spicy Laban Drink	7	not permitted (high salt)	YOGHURTS, SOUR MILK, CREAM AND OTHER SIMILAR FOODS	0.85	1.68	28.73	0.54	0.90
UIL.21.13547	Mazoon Strawberry Laban	7	not permitted (high totalfat)	YOGHURTS, SOUR MILK, CREAM AND OTHER SIMILAR FOODS	3.49	14.27	102.01	2.30	0.14

Sample ID (from lab)	Name of Product & Brand	Nutrient Profile Group	Marketing Permitted/not permitted	Food category	Amounts for 100 g/ml of the product				
					Total fat (g)	Total Sugars (g)	Energy (kcal)	Sat fat (g)	Salt (g)
UIL.21.13830	Nestle cream	7	not permitted (high total fat & high saturated fat)	YOGHURTS, SOUR MILK, CREAM AND OTHER SIMILAR FOODS	23.44	0.00	242.92	13.40	0.00
UIL.21.13557	Yoghurt: Full Cream Unikai	7	not permitted (high totalfat & high saturatedfat & high salt)	YOGHURTS, SOUR MILK, CREAM AND OTHER SIMILAR FOODS	3.19	4.41	62.11	2.41	0.21
UIL.21.13559	Youghurt;Full Cream AlRawabi	7	not permitted (high totalfat & high saturatedfat & high salt)	YOGHURTS, SOUR MILK, CREAM AND OTHER SIMILAR FOODS	3.31	4.85	68.79	2.37	0.22
UIL.21.13840	Almarai Burger Slice Cheese	9	not permitted (high total fat & high salt)	CHEESE	25.30	0.00	311.50	15.15	3.50
UIL.21.13845	Almarai Cream Cheese	9	not permitted (high total fat & high salt)	CHEESE	34.10	0.00	344.77	25.13	1.89
UIL.21.13844	Almarai Cream Cheese Cheddar	9	not permitted (high total fat & high salt)	CHEESE	30.87	0.00	336.87	15.50	2.93
UIL.21.14572	Chedder cheese (Almarai)	9	not permitted (high total fat & high salt)	CHEESE	24.97	0.00	296.30	16.77	4.82
UIL.21.14571	Chedder cheese (Kraft)	9	not permitted (high total fat & high salt)	CHEESE	25.56	0.00	306.84	17.84	4.57
UIL.21.13837	Cheese Triangles Al Maraai	9	not permitted (high total fat & high salt)	CHEESE	27.30	0.00	301.17	17.87	2.42
UIL.21.13838	Cheese Triangles Puck	9	not permitted (high total fat & high salt)	CHEESE	25.52	0.00	286.88	12.95	2.79
UIL.21.13834	Haloumi Cheese Al-Marai	9	not permitted (high total fat & high salt)	CHEESE	27.10	0.00	353.50	24.20	3.19
UIL.21.13835	Haloumi Pinar	9	not permitted (high total fat & high salt)	CHEESE	25.87	0.00	345.91	14.12	4.82
UIL.21.13848	Kraft Cheddar Cheese Spread Original	9	not permitted (high total fat & high salt)	CHEESE	28.21	0.00	304.09	19.12	3.09
UIL.21.13839	Kraft Slice Cheese Original	9	not permitted (high total fat & high salt)	CHEESE	21.56	0.00	284.32	13.17	3.44
UIL.21.13843	Mazoon Spread Cheese	9	not permitted (high total fat)	CHEESE	33.48	0.00	340.52	24.12	1.01
UIL.21.13842	Mazoon Spread Cheese Cheddar	9	not permitted (high total fat)	CHEESE	33.52	0.00	339.60	23.82	1.00
UIL.21.14554	Pinar Processed Cream Cheese Spread	9	not permitted (high total fat & high salt)	CHEESE	31.02	0.00	312.26	27.80	1.80
UIL.21.13847	Puck Cream Cheese	9	not permitted (high total fat & high salt)	CHEESE	31.85	0.00	331.21	20.97	2.12
UIL.21.13846	Puck Cream Cheese Cheddar	9	not permitted (high total fat & high salt)	CHEESE	32.01	0.00	332.65	19.20	2.02
UIL.21.13841	Puck Slice Cheese Original	9	not permitted (high total fat & high salt)	CHEESE	27.66	0.00	330.90	14.04	1.55
UIL.21.13836	Triangle Cheese Abu Alwald	9	not permitted (high total fat & high salt)	CHEESE	21.02	0.00	254.50	11.92	1.89
UIL.21.13456	Britannia Toastea Wheat Rusk	11	not permitted (high sugar)	BREAD, BREAD PRODUCTS AND CRISP BREADS10	9.74	26.71	436.06	4.35	0.57
UIL.21.13455	Tiffany Whole Wheat Rusks	11	not permitted (high sugar)	BREAD, BREAD PRODUCTS AND CRISP BREADS9	8.49	23.11	424.65	3.80	0.57
UIL.21.13429	Indomie Fried Noodles	12	not permitted (high total fat & high salt)	FRESH, DRIED OR COOKED PASTA, RICE AND GRAINS	20.92	8.24	470.68	11.35	3.47
UIL.21.14202	Al-Islami Ground Beef	14	not permitted	PROCESSED MEAT, POULTRY AND SIMILAR	9.25	1.38	139.57	4.16	1.07
UIL.21.14203	Al-Kabeer Jumbo Hot Dogs	14	not permitted	PROCESSED MEAT, POULTRY AND SIMILAR	7.69	0.00	154.69	4.31	1.37
UIL.21.14194	Al-Safa Breaded Chicken Finger	14	not permitted	PROCESSED MEAT, POULTRY AND SIMILAR	8.31	0.00	228.71	2.56	1.23
UIL.21.14196	Al-Safa Chicken Burger	14	not permitted	PROCESSED MEAT, POULTRY AND SIMILAR	10.41	0.00	176.89	3.01	1.00
UIL.21.14205	Al-Safa Chicken Franks	14	not permitted	PROCESSED MEAT, POULTRY AND SIMILAR	14.88	0.00	206.53	8.06	3.30
UIL.21.14204	Americana Beef Hot dog	14	not permitted	PROCESSED MEAT, POULTRY AND SIMILAR	12.56	0.00	181.68	5.27	1.53
UIL.21.14200	Beef Burger (Taybat)	14	not permitted	PROCESSED MEAT, POULTRY AND SIMILAR	13.52	0.00	223.72	6.99	1.58
UIL.21.14199	Beef Burger Sadia	14	not permitted	PROCESSED MEAT, POULTRY AND SIMILAR	20.57	0.00	268.77	10.32	1.16
UIL.21.14201	Corned Beef (Taybat)	14	not permitted	PROCESSED MEAT, POULTRY AND SIMILAR	8.42	0.54	176.90	4.14	0.54
UIL.21.14573	Family Corned Beef	14	not permitted	PROCESSED MEAT, POULTRY AND SIMILAR	10.59	4.02	215.19	6.54	1.08
UIL.21.14198	Sadia Chicken Burger	14	not permitted	PROCESSED MEAT, POULTRY AND SIMILAR	15.24	0.00	221.12	5.93	1.51
UIL.21.14206	Sadia Chicken Franks	14	not permitted	PROCESSED MEAT, POULTRY AND SIMILAR	15.65	0.00	203.41	6.77	1.17
UIL.21.14195	Sadia Chicken Nuggets Crispy	14	not permitted	PROCESSED MEAT, POULTRY AND SIMILAR	10.35	0.00	224.19	3.21	1.06

Sample ID (from lab)	Name of Product & Brand	Nutrient Profile Group	Marketing Permitted/not permitted	Food category	Amounts for 100 g/ml of the product				
					Total fat (g)	Total Sugars (g)	Energy (kcal)	Sat fat (g)	Salt (g)
UIL.21.13295	Light Chunks Tuna California Garden In Water	15	not permitted (high salt)	PROCESSED FISH	1.07	0.84	108.19	0.80	0.55
UIL.21.13291	Skipjack Tuna Alalali In Sun Flower Oil	15	permitted	PROCESSED FISH	11.02	0.80	207.50	1.46	1.22
UIL.21.13294	Skipjack Tuna California Garden In Sun Flower Oil	15	permitted	PROCESSED FISH	2.98	0.89	135.02	0.36	0.77
UIL.21.13292	Tuna Alalali In Water	15	permitted	PROCESSED FISH	0.01	0.89	106.01	0.00	1.22
UIL.21.13434	White Tuna Meat In Brine	15	permitted	PROCESSED FISH	0.61	0.10	115.65	0.46	0.97
UIL.21.13293	White Tuna Meat In Sun Flower Oil Delmonte	15	permitted	PROCESSED FISH	5.58	0.36	161.06	0.80	1.08
UIL.21.13282	Baked Beans Almazraa	17	permitted	PROCESSED FRUIT, VEGETABLES AND LEGUMES	0.63	6.12	127.15	0.16	1.00
UIL.21.13278	Baked Beans California Garden	17	permitted	PROCESSED FRUIT, VEGETABLES AND LEGUMES	1.20	10.40	81.26	0.38	0.79
UIL.21.13289	Boildchick Peas Mara	17	permitted	PROCESSED FRUIT, VEGETABLES AND LEGUMES	1.31	0.74	73.71	0.26	0.39
UIL.21.13286	Chick Peas Almazraa	17	permitted	PROCESSED FRUIT, VEGETABLES AND LEGUMES	0.61	0.81	94.18	0.11	0.75
UIL.21.13281	Chick Peas California Garden	17	not permitted (high add sugar)	PROCESSED FRUIT, VEGETABLES AND LEGUMES	0.64	0.84	114.15	0.13	0.68
UIL.21.13284	Chick Peas Luna	17	permitted	PROCESSED FRUIT, VEGETABLES AND LEGUMES	1.60	0.76	99.00	0.74	0.88
UIL.21.14397	Diamond Strawberry Jam	17	not permitted (high sugar)	PROCESSED FRUIT, VEGETABLES AND LEGUMES	0.31	63.84	270.23	0.27	0.04
UIL.21.13283	Foul Medamas Almazraa	17	not permitted (high add sugar)	PROCESSED FRUIT, VEGETABLES AND LEGUMES	0.01	0.77	81.21	0.00	1.10
UIL.21.14557	Foul Medammas (American Garden)	17	not permitted (high add sugar)	PROCESSED FRUIT, VEGETABLES AND LEGUMES	0.01	1.22	93.52	0.00	0.87
UIL.21.13276	Green Giant Sweet Corn	17	permitted	PROCESSED FRUIT, VEGETABLES AND LEGUMES	1.63	7.48	83.33	0.38	0.52
UIL.21.13285	Green Peas Luna	17	permitted	PROCESSED FRUIT, VEGETABLES AND LEGUMES	0.42	0.61	86.50	0.12	0.60
UIL.21.14555	La Ming Beans In Tomato Sauce	17	permitted	PROCESSED FRUIT, VEGETABLES AND LEGUMES	0.98	2.40	392.74	0.23	0.00
UIL.21.14556	Luna Baked Beans In Tomato Sauce	17	permitted	PROCESSED FRUIT, VEGETABLES AND LEGUMES	0.30	3.96	88.74	0.19	0.81
UIL.21.14558	Lunafoul Medames Extra Grade Fava Beans	17	permitted	PROCESSED FRUIT, VEGETABLES AND LEGUMES	0.41	1.17	99.61	0.21	0.84
UIL.21.14406	Mango Pickle / Aeroplane	17	not permitted (high salt)	PROCESSED FRUIT, VEGETABLES AND LEGUMES	4.08	0.10	81.04	1.23	12.80
UIL.21.14403	Mango Pickles In Oil / Ahmed	17	not permitted (high total fat & high salt)	PROCESSED FRUIT, VEGETABLES AND LEGUMES	20.80	0.50	236.80	2.30	7.66
UIL.21.14405	Mango Thokku Pickle In Oil / Priya	17	not permitted (high sugar & high salt)	PROCESSED FRUIT, VEGETABLES AND LEGUMES	4.21	22.40	166.33	1.03	11.36
UIL.21.14559	Peas processed	17	not permitted (high add sugar)	PROCESSED FRUIT, VEGETABLES AND LEGUMES	0.01	2.46	84.81	0.00	0.55
UIL.21.13277	Plain Medammes California Garden	17	not permitted (high add sugar)	PROCESSED FRUIT, VEGETABLES AND LEGUMES	0.01	0.93	94.19	0.00	0.87
UIL.21.13287	Processed Peas Almazraa	17	permitted	PROCESSED FRUIT, VEGETABLES AND LEGUMES	0.01	1.64	79.67	0.00	0.83
UIL.21.13279	Processed Peas California Garden	17	permitted	PROCESSED FRUIT, VEGETABLES AND LEGUMES	0.01	1.64	83.92	0.15	0.84
UIL.21.13290	Sweet Corn American Garden	17	permitted	PROCESSED FRUIT, VEGETABLES AND LEGUMES	1.29	6.41	90.54	0.29	0.45
UIL.21.13280	Sweet Corn California Garden	17	permitted	PROCESSED FRUIT, VEGETABLES AND LEGUMES	1.08	3.08	98.22	0.28	0.34
UIL.21.13288	Sweet Corn Libbys	17	permitted	PROCESSED FRUIT, VEGETABLES AND LEGUMES	0.55	2.09	50.06	0.16	0.57
UIL.21.14404	Tomato Paste / Alalali	17	not permitted (high sugar & high salt)	PROCESSED FRUIT, VEGETABLES AND LEGUMES	0.72	12.30	81.48	0.38	1.04
UIL.21.14399	Tomato Paste / Almudhish	17	not permitted (high sugar)	PROCESSED FRUIT, VEGETABLES AND LEGUMES	1.02	10.40	97.14	0.39	0.00
UIL.21.14400	Tomato Paste / Jumbo	17	permitted	PROCESSED FRUIT, VEGETABLES AND LEGUMES	0.65	4.14	85.25	0.25	0.77
UIL.21.14394	American Garden BBQ Sauce Original	18	not permitted (high salt)	SAUCES AND DRESSINGS	0.03	34.14	166.92	0.01	2.51
UIL.21.14415	Classic Mayonnaise / Mazola	18	not permitted (high total fat & high salt)	SAUCES AND DRESSINGS	79.21	3.71	727.50	13.64	1.27
UIL.21.14416	Creamy Classic Mayonnaise / Heinz	18	not permitted (high total fat & high salt)	SAUCES AND DRESSINGS	35.85	3.46	371.49	7.32	2.05
UIL.21.14393	Delicio 1000 Island	18	not permitted (high total fat & high salt)	SAUCES AND DRESSINGS	36.93	14.29	374.36	6.64	2.64
UIL.21.14392	Delicio French Dressing	18	not permitted (high total fat & high salt)	SAUCES AND DRESSINGS	36.86	19.79	398.00	6.64	2.84
UIL.21.13296	Hayat Hot Sauce	18	not permitted (high salt)	SAUCES AND DRESSINGS	0.70	0.72	11.90	0.00	6.64
UIL.21.14395	Heinz Classic BBQ Smokey And Rich	18	not permitted (high salt)	SAUCES AND DRESSINGS	0.35	28.68	141.63	0.15	1.26

Sample ID (from lab)	Name of Product & Brand	Nutrient Profile Group	Marketing Permitted/not permitted	Food category	Amounts for 100 g/ml of the product				
					Total fat (g)	Total Sugars (g)	Energy (kcal)	Sat fat (g)	Salt (g)
UIL.21.14402	Hot Sauce / Excellence	18	not permitted (high salt)	SAUCES AND DRESSINGS	0.01	9.79	43.89	0.00	5.82
UIL.21.14417	Hot Sause / American Garden	18	not permitted (high salt)	SAUCES AND DRESSINGS	0.20	2.00	12.20	0.04	7.09
UIL.21.13297	Jumbo Hot Sauce	18	not permitted (high salt)	SAUCES AND DRESSINGS	0.10	0.58	5.70	0.00	4.09
UIL.21.14562	Knour Beef Flavoured Stock	18	not permitted (high salt)	SAUCES AND DRESSINGS	0.00	0.00	3.08	0.00	1.17
UIL.21.14561	Knour CHICKEN Stock	18	not permitted (high salt)	SAUCES AND DRESSINGS	0.00	0.00	2.72	0.00	1.37
UIL.21.14563	Knour Vegetable Stock Cubes	18	not permitted (high salt)	SAUCES AND DRESSINGS	0.00	0.00	2.92	0.00	1.12
UIL.21.14564	Maggi Beef Flavour	18	not permitted (high salt)	SAUCES AND DRESSINGS	0.00	0.00	51.76	0.00	50.55
UIL.21.13430	Maggi Chicken Stock	18	not permitted (high total fat & high salt)	SAUCES AND DRESSINGS	21.89	8.23	275.69	12.87	46.28
UIL.21.14565	Maggi Chicken Stock Less Salt	18	not permitted (high salt)	SAUCES AND DRESSINGS	0.00	0.00	116.40	0.00	37.54
UIL.21.14407	Pizza Sauce Olive& Mushrooms / Alalali	18	not permitted (high add sugar)	SAUCES AND DRESSINGS	1.58	6.38	61.02	0.36	0.91
UIL.21.14398	Soy Sauce Classic / American Garden	18	not permitted (high salt)	SAUCES AND DRESSINGS	0.20	2.00	12.20	0.04	7.25
UIL.21.14401	Tabasco Hot Sauce	18	not permitted (high salt)	SAUCES AND DRESSINGS	0.01	2.65	17.81	0.00	1.75
UIL.21.14408	Tomato Ketchup / Hayat	18	not permitted (high salt)	SAUCES AND DRESSINGS	0.07	22.20	109.40	0.01	2.07
UIL.21.14413	Tomato Ketchup / Heinz	18	not permitted (high salt & high add sugar)	SAUCES AND DRESSINGS	0.01	22.58	98.89	0.00	1.83
UIL.21.14411	Tomato Ketchup/ Jumbo	18	not permitted (high add sugar)	SAUCES AND DRESSINGS	0.31	26.94	124.15	0.19	0.42
UIL.21.13828	Al Deyafa Fruit Cordial	3a	not permitted	BEVERAGES: a) Fruit juices	0.00	60.59	257.32	0.00	0.04
UIL.21.13850	Al Deyafa Orange Flavour (Powder)	3a	not permitted	BEVERAGES: a) Fruit juices	0.00	94.69	382.96	0.00	0.00
UIL.21.14134	Al Marai Mango Juice	3a	not permitted	BEVERAGES: a) Fruit juices	0.10	11.81	64.02	0.00	0.00
UIL.21.14132	Al Rabie Fruit Cocktail Nectar	3a	not permitted	BEVERAGES: a) Fruit juices	0.00	12.47	61.96	0.00	0.00
UIL.21.14133	Al Rawabi Apple Juice	3a	not permitted	BEVERAGES: a) Fruit juices	0.10	9.15	43.34	0.02	0.00
UIL.21.14138	Al Rawabi Red Grape Juice	3a	not permitted	BEVERAGES: a) Fruit juices	0.10	13.96	62.62	0.00	0.00
UIL.21.14135	Almarai Graps And Berries (Farms Select)	3a	not permitted	BEVERAGES: a) Fruit juices	0.05	13.61	57.81	0.00	0.00
UIL.21.13822	Almarai Orange Juice 100% 300 ml	3a	not permitted	BEVERAGES: a) Fruit juices	0.00	9.39	47.95	0.00	0.00
UIL.21.13823	Alrawabi Orange 1005juice	3a	not permitted	BEVERAGES: a) Fruit juices	0.00	6.65	44.00	0.00	0.15
UIL.21.13821	Asafwa Natural Orange 200 ml	3a	not permitted	BEVERAGES: a) Fruit juices	0.00	10.25	51.76	0.00	0.42
UIL.21.14139	A'safwah Mixed Fruit Juice	3a	not permitted	BEVERAGES: a) Fruit juices	0.10	11.56	57.50	0.00	0.00
UIL.21.14143	Fruit Drink (Orange) , Rani	3a	not permitted	BEVERAGES: a) Fruit juices	0.10	11.56	54.06	0.00	0.02
UIL.21.14142	Fruit Nectar (Lacnor)	3a	not permitted	BEVERAGES: a) Fruit juices	0.10	12.73	54.06	0.00	0.00
UIL.21.13829	Mango Juice Mazoon 200ml	3a	not permitted	BEVERAGES: a) Fruit juices	0.00	13.44	65.76	0.00	0.00
UIL.21.13824	Mixed Fruit Juice (Mazoon)	3a	not permitted	BEVERAGES: a) Fruit juices	0.00	11.77	52.96	0.00	0.00
UIL.21.14136	Nada Mango Juice	3a	not permitted	BEVERAGES: a) Fruit juices	0.01	10.50	52.21	0.00	0.00
UIL.21.14137	Nada Orange Juice	3a	not permitted	BEVERAGES: a) Fruit juices	0.05	10.46	44.33	0.00	0.00
UIL.21.13827	Natural Mango Asafwa 200ml	3a	not permitted	BEVERAGES: a) Fruit juices	0.00	13.13	54.96	0.00	0.01
UIL.21.14141	Orange Drink (Top Fruit)	3a	not permitted	BEVERAGES: a) Fruit juices	0.10	10.72	48.94	0.00	0.01
UIL.21.13826	Orange No Add Sugar (Top Fruit)	3a	not permitted	BEVERAGES: a) Fruit juices	0.00	9.14	41.80	0.00	0.02
UIL.21.14140	Sun Top Orange Fruit Drink	3a	not permitted	BEVERAGES: a) Fruit juices	0.10	11.84	52.98	0.00	0.01
UIL.21.17402	Tang (Orange) (Powder)	3a	not permitted	BEVERAGES: a) Fruit juices	0.00	96.92	387.84	0.00	0.46
UIL.21.13825	Top fruit Fruit Cocktail 200ml	3a	not permitted	BEVERAGES: a) Fruit juices	0.00	9.28	45.52	0.00	0.06
UIL.21.13851	Vimto	3a	not permitted	BEVERAGES: a) Fruit juices	0.00	12.39	50.32	0.00	0.00
UIL.21.14129	Evaporated Milk (Rainbow)	3c	not permitted (high total fat)	BEVERAGES: c) Milk Drinks	8.85	9.54	145.69	6.25	0.27

Sample ID (from lab)	Name of Product & Brand	Nutrient Profile Group	Marketing Permitted/not permitted	Food category	Amounts for 100 g/ml of the product				
					Total fat (g)	Total Sugars (g)	Energy (kcal)	Sat fat (g)	Salt (g)
UIL.21.13450	Galaxy Smooth Milk	3c	not permitted (high add sugar)	BEVERAGES: c) Milk Drinks	0.38	73.08	388.08	0.08	0.00
UIL.21.13551	Mazoon Fresh Milk Strawberry Flavour	3c	not permitted (high total fat)	BEVERAGES: c) Milk Drinks	3.21	6.47	84.17	2.36	0.14
UIL.21.14570	Milk Powder (Almudhish)	3c	not permitted (high total fat)	BEVERAGES: c) Milk Drinks	29.52	38.95	526.76	19.24	0.71
UIL.21.14569	Milk Powder (Majan)	3c	not permitted (high total fat)	BEVERAGES: c) Milk Drinks	29.21	47.83	523.25	20.19	0.84
UIL.21.13553	Strawberry flavored milk	3c	not permitted (high add sugar)	BEVERAGES: c) Milk Drinks	2.50	12.03	83.21	1.74	0.15
UIL.21.14171	Strawberry Flavored Milk (Lacnor)	3c	not permitted (high total fat & high add sugar)	BEVERAGES: c) Milk Drinks	3.26	9.84	84.46	1.94	0.18
UIL.21.13554	Strawberry Fresh Milk (Alrawabi)	3c	not permitted (high total fat)	BEVERAGES: c) Milk Drinks	3.28	11.77	91.56	2.32	0.38
UIL.21.14170	Strawberry Milk (Nada)	3c	not permitted (high total fat & high add sugar)	BEVERAGES: c) Milk Drinks	3.21	9.67	84.33	2.27	0.12
UIL.21.13552	Strwaberry Milk (Asafwah)	3c	permitted	BEVERAGES: c) Milk Drinks	2.42	14.14	92.06	1.78	0.15
UIL.21.14130	Tea Milk (Almudhish)	3c	not permitted (high total fat)	BEVERAGES: c) Milk Drinks	8.12	9.28	136.88	4.05	0.27
UIL.21.14173	Energy drink (Red bull)	3d	not permitted	BEVERAGES: d) Energy Drinks	0.01	10.15	46.45	0.00	0.11
UIL.21.13849	Power Horse Energy	3d	not permitted	BEVERAGES: d) Energy Drinks	0.00	11.28	47.42	0.00	0.17
UIL.21.14174	Coca cola	3e	not permitted (high sugar)	BEVERAGES: e) Other Drinks	0.01	9.78	43.93	0.00	0.04
UIL.21.14172	Lipton Peach Ice tea	3e	not permitted (high sugar)	BEVERAGES: e) Other Drinks	0.01	4.40	18.53	0.00	0.03
UIL.21.14177	Malt beverage (Barbican)	3e	not permitted (high sugar)	BEVERAGES: e) Other Drinks	0.01	2.22	13.01	0.00	0.03
UIL.21.14176	Mountain Dew	3e	not permitted (high sugar)	BEVERAGES: e) Other Drinks	0.01	11.55	49.37	0.00	0.04
UIL.21.14175	Pepsi	3e	not permitted (high sugar)	BEVERAGES: e) Other Drinks	0.01	9.98	40.93	0.00	0.03
UIL.21.14178	Vitaene Extra (Pokka)	3e	not permitted (high sugar)	BEVERAGES: e) Other Drinks	0.01	13.36	59.21	0.00	0.00

Table S4: Nutrient composition of sampled food products.

Sample ID (from lab)	Name of Product & Brand	Amounts per 100 g/ml from Laboratory Analysis														Amounts per 100 g/ml calculated		
		Total Fat (g)	SFA (g)	PUFA (g)	MUFA (g)	TFA (g)	Sodium (mg)	Total Sugars (g)	Glucose (g)	Fructose (g)	Sucrose (g)	Maltosa (g)	Lactose (g)	Protein (g)	Carbo-hydrate (g)	Energy (kJ)	Energy (kcal)	Salt (g)
UIL.21.14412	Pure Honey / Capilano	0.00	0.00	0.00	0.00	0.00	0.00	81.00	37.20	43.80	0.10	0.10	0.10	0.00	81.94	1392.98	327.76	0.00
UIL.21.14414	Natural Honey / Alshifa	0.00	0.00	0.00	0.00	0.00	0.01	80.30	37.09	42.94	0.10	0.10	0.10	0.00	83.64	1421.88	334.56	0.00
UIL.21.13443	Wrigleys Doublemint Chewing Gum	0.38	0.08	0.08	0.08	0.08	0.04	81.54	81.54	3.85	3.85	3.85	3.85	0.77	91.54	1583.46	372.69	0.00
UIL.21.14391	Nutella Hazelnut Spread With Cocoa	31.20	10.53	6.07	14.05	0.09	43.88	57.94	0.10	0.10	52.54	0.10	5.40	5.40	60.98	2282.86	546.32	0.11
UIL.21.13440	Mars Minis	17.92	11.54	0.02	0.02	0.02	222.31	57.77	10.38	0.77	47.31	0.77	0.77	4.31	73.69	1989.15	473.31	0.56
UIL.21.13441	Extra Peppermint	0.53	0.11	0.11	0.11	0.11	0.05	52.63	52.63	5.26	5.26	5.26	5.26	1.05	125.26	2166.84	510.00	0.00
UIL.21.13438	Snickers Minis	26.80	6.73	3.80	12.13	4.13	263.33	52.53	10.27	0.67	42.20	0.67	0.67	9.27	57.73	2130.60	509.20	0.67
UIL.21.13435	Kinder Joy	32.22	15.15	3.60	13.46	0.00	191.50	49.62	0.00	0.10	49.62	0.10	0.10	8.72	55.23	2279.29	545.78	0.49
UIL.21.13454	Twix Minis	25.35	11.45	3.95	10.00	0.01	186.00	48.60	6.50	0.00	36.30	0.00	5.90	4.85	68.95	2192.55	523.35	0.47
UIL.21.13437	Kitkat	25.29	13.76	1.63	9.89	0.00	73.00	45.37	0.10	0.10	45.37	0.10	0.10	8.03	63.82	2157.18	515.01	0.19
UIL.21.13444	Mentos Sugar free Chewing Gum	0.57	0.11	0.11	0.11	0.11	0.06	32.00	4.00	5.71	28.00	5.71	5.71	1.14	93.14	1624.00	382.29	0.00
UIL.21.13560	Cream Caramel Almaraei	2.23	1.66	0.11	0.40	0.05	60.20	20.92	1.89	1.52	12.15	0.00	5.37	3.23	22.76	524.34	124.03	0.15
UIL.21.13550	Danet Cream Caramel	2.21	1.59	0.11	23.00	0.00	62.50	17.80	1.94	1.12	10.24	0.00	4.50	3.41	20.38	486.20	115.05	0.16
UIL.21.13439	Bounty Minis	14.34	8.38	2.02	6.57	0.00	321.20	15.02	1.64	0.10	13.38	0.10	0.10	9.54	67.70	1843.66	438.02	0.82
UIL.21.14396	Daily Fresh/ Custard Powder	0.64	0.21	0.20	0.23	0.00	699.82	3.07	3.07	0.10	0.10	0.10	0.10	0.33	83.23	1444.20	340.00	1.78
UIL.21.13442	Batook Specialmint Chewing Gum	0.40	0.08	0.08	0.08	0.08	0.04	2.00	2.00	4.00	4.00	4.00	4.00	1.20	90.40	1572.00	370.00	0.00
UIL.21.14409	Custard Powder/Tiara	0.23	0.14	0.06	0.03	0.00	0.13	0.21	0.10	0.10	0.21	0.10	0.10	0.32	87.95	1509.10	355.15	0.00
UIL.21.14410	Custard Powder / Alalali	0.01	0.00	0.00	0.00	0.00	81.20	0.10	0.10	0.10	0.10	0.10	0.10	0.01	90.75	1543.29	363.13	0.21
UIL.21.13458	Nabil Cracker Salted Biscuits	19.26	13.50	1.50	4.27	0.00	998.60	20.04	0.85	0.92	18.27	0.10	0.10	9.58	65.90	1995.78	475.26	2.54
UIL.21.14390	Mani (Mixed Nuts)	49.33	7.92	18.57	22.84	0.00	316.23	8.55	1.78	0.10	6.34	0.10	0.43	26.01	18.95	2589.53	623.81	0.80
UIL.21.17408	Cheese Balls (Mr. Krispy)	34.33	7.67	15.67	11.00	0.01	1541.53	7.47	1.40	1.53	3.73	0.67	0.67	6.80	61.87	2437.67	583.67	3.92

Sample ID (from lab)	Name of Product & Brand	Amounts per 100 g/ml from Laboratory Analysis													Amounts per 100 g/ml calculated		
		Total Fat (g)	SFA (g)	PUFA (g)	MUFA (g)	TFA (g)	Sodium (mg)	Total Sugars (g)	Glucose (g)	Fructose (g)	Sucrose (g)	Maltosa (g)	Lactose (g)	Protein (g)	Carbo-hydrate (g)	Energy (kJ)	Energy (kcal)	Salt (g)
UIL.21.13433	Alrifai Mixed Nuts	32.43	4.64	9.36	18.43	0.00	240.00	7.18	0.29	0.36	6.89	0.36	0.36	21.39	40.00	2243.49	537.42	0.61
UIL.21.14388	Shai (Mix Nut)	48.44	6.87	11.49	30.08	0.00	150.60	6.85	0.18	0.10	6.09	0.10	0.48	25.21	20.69	2572.58	619.56	0.38
UIL.21.14384	Al Mudhish Puffed Corn Cheese Balls	35.75	16.58	4.00	15.17	0.02	621.00	4.42	2.00	0.08	0.25	0.83	2.00	6.42	55.17	2369.67	568.08	1.58
UIL.21.13432	Best Salted Mixed Nuts	51.89	8.31	15.15	28.43	0.00	336.10	4.28	0.39	0.10	3.89	0.10	0.10	24.52	19.56	2669.29	643.33	0.85
UIL.21.14418	Cheese Balls (Pofak Oman)	39.15	16.79	6.37	15.99	0.00	589.13	4.17	1.06	0.10	0.20	0.10	2.91	9.89	46.98	2415.34	579.83	1.50
UIL.21.14551	Cheese Pastry (Dhahabi)	18.54	4.74	7.99	5.81	0.00	306.93	3.85	0.65	1.53	1.06	0.62	0.10	11.57	41.35	1585.62	378.54	0.78
UIL.21.13463	Cheetos / Flamin Hot	34.63	9.73	4.40	19.37	0.01	700.67	1.07	1.07	0.33	0.33	0.33	0.33	5.93	59.57	2394.93	573.70	1.78
UIL.21.13462	Pringles Original	33.49	3.53	10.01	19.95	0.00	452.50	1.05	0.16	0.13	0.75	0.10	0.10	4.53	58.06	2303.16	551.77	1.15
UIL.21.13431	American Garden Popcorn Butter	24.42	10.91	4.82	8.73	0.01	985.15	0.30	0.30	0.30	0.30	0.30	0.30	7.70	55.15	1972.12	471.21	2.50
UIL.21.14389	Popcorn Cheese / American Garden	34.21	15.67	7.24	11.30	0.01	988.85	0.00	0.00	0.00	0.00	0.00	0.00	6.58	48.52	2202.39	528.27	2.51
UIL.21.14560	Sohar Chips	32.36	14.31	5.25	12.80	0.00	950.68	0.00	0.00	0.00	0.00	0.00	0.00	3.18	60.81	2285.15	547.20	2.41
UIL.21.14383	Lay's (Salt)	35.50	16.07	4.57	14.79	0.01	594.36	0.00	0.00	0.00	0.00	0.00	0.00	7.50	53.71	2354.14	564.36	1.51
UIL.21.14385	Potato Chips Ready Salted	36.11	15.75	4.96	15.39	0.01	535.71	0.00	0.00	0.00	0.00	0.00	0.00	7.11	52.14	2343.21	561.96	1.36
UIL.21.14386	Chips Oman	38.77	16.89	5.29	16.58	0.00	503.20	0.00	0.00	0.00	0.00	0.00	0.00	6.54	47.63	2355.38	565.61	1.28
UIL.21.13833	Vanilla Ice cream (Igloo)	11.13	6.68	0.81	3.64	0.00	67.74	18.60	0.19	0.19	13.13	0.19	5.47	3.74	20.53	824.38	197.25	0.17
UIL.21.13832	Vanilla Ice cream (Mazoon)	7.66	4.46	0.54	2.67	0.00	45.60	12.37	2.39	1.36	5.75	2.87	0.10	3.09	13.73	569.36	136.22	0.12
UIL.21.13299	Nestle Chocapic	4.79	1.50	0.91	2.37	0.00	102.00	24.95	0.51	0.14	24.29	0.10	0.10	8.76	82.06	1721.17	406.39	0.26
UIL.21.13300	Kelloggs Coco Pops	2.48	1.15	0.48	0.85	0.00	0.31	24.48	0.22	0.07	28.07	0.10	0.10	9.54	80.62	1624.48	382.96	0.00
UIL.21.14387	Kelloggs Special (Cereal)	2.21	0.59	0.82	0.08	0.01	695.69	13.92	1.72	0.33	10.82	1.05	0.26	18.26	72.92	1631.64	384.56	1.77
UIL.21.13298	Nestle Gold Corn Flakes	1.96	0.38	1.08	0.50	0.00	426.00	10.57	0.73	0.79	8.43	0.10	0.10	7.24	84.19	1626.83	383.36	1.08
UIL.21.13459	Original Oreo	19.47	8.79	2.74	7.95	0.01	582.11	42.11	0.47	0.16	41.47	0.00	0.00	5.32	71.58	2027.74	482.84	1.48
UIL.21.13457	Lotus Biscoff	19.36	13.59	1.54	4.23	0.03	412.82	36.41	0.13	0.00	36.28	0.00	0.00	5.64	73.72	2065.38	491.67	1.05
UIL.21.13453	Tiffany Crunch And Cream	24.36	21.51	0.57	2.27	0.00	120.30	36.23	0.10	0.10	28.09	0.00	8.13	8.65	64.63	2147.08	512.36	0.31

Sample ID (from lab)	Name of Product & Brand	Amounts per 100 g/ml from Laboratory Analysis														Amounts per 100 g/ml calculated		
		Total Fat (g)	SFA (g)	PUFA (g)	MUFA (g)	TFA (g)	Sodium (mg)	Total Sugars (g)	Glucose (g)	Fructose (g)	Sucrose (g)	Maltosa (g)	Lactose (g)	Protein (g)	Carbo-hydrate (g)	Energy (kJ)	Energy (kcal)	Salt (g)
UIL.21.13452	Nutro chocolate flavoured cream wafers	30.20	26.80	0.83	2.53	0.00	204.00	36.17	0.33	0.33	31.27	0.33	4.90	3.50	62.53	2239.97	535.93	0.52
UIL.21.13451	Britannia chocolate flavoured premium crème wafer	25.38	15.75	1.25	8.34	0.00	247.50	33.81	0.31	0.31	24.25	0.31	0.31	6.88	64.50	2152.25	513.88	0.63
UIL.21.13460	Americana premium butter cookies	20.27	9.16	2.33	8.77	0.00	112.50	29.40	0.70	0.48	28.22	0.00	0.00	6.17	70.51	2053.55	489.15	0.29
UIL.21.14567	Switz Mini cupcake vanilla	29.41	12.75	4.91	11.53	0.01	404.19	29.00	0.34	0.91	27.78	0.31	0.31	7.13	48.59	2035.25	487.53	1.03
UIL.21.14566	Atyab twin cake vanilla	18.43	7.17	2.14	9.12	0.00	221.84	27.82	2.09	0.10	25.73	0.10	0.10	5.82	56.21	1736.42	413.99	0.56
UIL.21.13461	Danish Butter Cookies	25.44	18.34	1.20	5.09	0.85	125.60	26.04	0.15	0.15	25.73	0.10	0.10	5.52	64.88	2138.08	510.56	0.32
UIL.21.13449	Tiffany Everyday Nice (Sugar Sprinkled Coconut Biscuits)	20.32	9.16	2.58	3.55	0.01	354.84	25.48	0.32	0.32	25.16	0.32	0.32	6.45	71.29	2073.55	493.87	0.90
UIL.21.13448	Tiffany Glucose (Milk and Honey Biscuits)	17.84	9.16	2.26	6.45	0.01	356.45	22.90	0.32	0.32	22.26	0.32	0.32	7.42	72.26	2014.55	479.26	0.91
UIL.21.13447	Nabil Nice (Sugar Sprinkled Coconut Biscuits)	15.36	7.12	2.94	5.32	0.00	375.20	21.16	0.75	0.54	19.87	0.10	0.10	9.52	71.75	1949.91	463.32	0.95
UIL.21.13446	Nabil Cream Wafers (Chocolate Flavour)	21.29	12.74	2.27	6.28	0.00	147.20	20.63	0.25	0.10	20.38	0.10	0.10	5.61	70.25	2077.35	495.05	0.37
UIL.21.17407	Donut (Switz)	17.57	5.57	7.86	4.14	0.01	240.66	19.74	4.14	3.51	12.09	0.29	0.29	6.63	55.09	1699.29	405.00	0.61
UIL.21.17405	Apple Puff (Lusine)	13.10	5.89	4.57	2.64	0.00	275.09	19.66	2.03	1.71	15.91	0.14	0.14	8.90	47.91	1450.54	345.16	0.70
UIL.21.14574	7 Days Mini Croissant	28.55	16.73	3.00	8.82	0.02	203.00	16.82	3.64	2.27	10.36	0.55	0.91	9.45	44.27	1969.55	471.82	0.52
UIL.21.14552	Apple Pastry	15.74	8.37	2.63	4.74	0.00	12.45	16.32	5.13	6.81	2.73	1.65	0.10	5.91	48.89	1513.98	360.86	0.03
UIL.21.13445	Nabil Glucose	14.36	7.42	2.02	4.92	0.00	0.01	14.88	0.41	0.34	14.12	0.10	0.10	9.30	73.57	1940.11	460.72	0.00
UIL.21.14568	Chocolate Jumbo Croissant	23.69	13.05	2.74	7.90	0.00	199.78	12.08	2.13	2.42	7.21	0.32	0.10	7.75	44.45	1763.93	422.01	0.51
UIL.21.17531	Croissant (Dhahabi)	23.18	10.21	9.85	3.12	0.00	621.51	11.95	3.12	1.89	6.94	0.10	0.10	7.21	50.11	1832.10	437.90	1.58
UIL.21.17406	Cheese Puff (Lusine)	22.03	10.30	4.43	7.31	0.00	616.04	8.77	2.31	1.71	4.74	0.14	0.14	10.17	36.17	1602.89	383.63	1.56
UIL.21.13548	Almarai Strawberry Laban	1.36	0.91	0.09	0.35	0.00	55.94	15.29	0.00	0.00	11.43	0.00	3.87	3.52	15.40	371.94	87.91	0.14
UIL.21.13547	Mazoon Strawberry Laban	3.49	2.30	0.18	0.93	0.00	54.30	14.27	0.10	0.10	8.91	0.10	5.36	3.28	14.37	429.18	102.01	0.14
UIL.21.13592	Fresh Labnah; Full Fat Al-Marai	12.85	8.82	1.07	2.98	0.04	223.64	7.33	1.18	0.18	0.18	0.18	6.15	5.91	8.85	726.60	174.75	0.57

Sample ID (from lab)	Name of Product & Brand	Amounts per 100 g/ml from Laboratory Analysis														Amounts per 100 g/ml calculated		
		Total Fat (g)	SFA (g)	PUFA (g)	MUFA (g)	TFA (g)	Sodium (mg)	Total Sugars (g)	Glucose (g)	Fructose (g)	Sucrose (g)	Maltosa (g)	Lactose (g)	Protein (g)	Carbo-hydrate (g)	Energy (kJ)	Energy (kcal)	Salt (g)
UIL.21.13594	Fresh Labnah: Full Fat Mazoon	11.13	7.82	0.73	2.56	0.01	213.50	6.63	1.79	0.10	0.13	0.10	4.71	6.54	7.16	644.71	154.97	0.54
UIL.21.13593	Labneh Turkish Reciepe: Puck	14.21	9.82	0.88	3.47	0.03	311.50	6.43	1.19	0.10	0.10	0.10	5.25	8.41	6.92	786.38	189.21	0.79
UIL.21.13558	Fresh Yougurt Full Fat: Al-Marai	3.61	2.57	0.82	0.20	0.01	71.29	5.62	0.59	0.06	0.06	0.06	4.97	4.23	5.75	303.34	72.44	0.18
UIL.21.13555	Mazoon Fresh Yoghurt Full Fat	3.21	2.29	0.17	0.73	0.01	58.30	5.04	0.41	0.10	0.10	0.10	4.63	3.41	6.38	285.20	68.05	0.15
UIL.21.13559	Youghurt; Full Cream Alrawabi	3.31	2.37	0.18	0.76	0.01	86.20	4.85	0.47	0.10	0.10	0.06	4.32	4.31	5.44	288.22	68.79	0.22
UIL.21.13556	Fresh Yougurt Full Fat: Al-Safwah	3.23	2.01	0.18	1.04	0.01	72.00	4.78	0.79	0.10	0.09	0.03	3.86	3.56	5.82	278.97	66.59	0.18
UIL.21.13542	Mazoon Fresh Laban Full Fat	3.30	2.22	0.22	0.83	0.00	51.55	4.48	0.47	0.10	0.12	0.10	3.88	4.09	4.64	270.51	64.62	0.13
UIL.21.17403	Activia Full Fat Fresh Laban	3.28	2.26	0.30	0.72	0.00	52.32	4.46	0.49	0.07	0.10	0.10	3.90	3.20	4.65	254.81	60.92	0.13
UIL.21.13557	Yoghurt: Full Cream Unikai	3.19	2.41	0.13	0.64	0.01	82.60	4.41	0.64	0.10	0.12	0.10	3.65	3.52	4.83	259.98	62.11	0.21
UIL.21.13544	Almarai Fresh Laban Full Fat	3.64	2.57	0.19	0.87	0.00	55.33	4.09	0.52	0.06	0.14	0.06	3.42	3.07	4.33	260.74	62.42	0.14
UIL.21.13543	Laban Fresh Full Creeam (Asafwah)	3.31	2.37	0.24	0.89	0.00	68.50	4.07	0.55	0.10	0.15	0.10	3.38	3.31	5.10	265.44	63.43	0.17
UIL.21.13545	Almarai Laban Up	1.15	0.81	0.07	0.27	0.00	316.10	3.99	1.53	0.05	0.05	0.05	2.46	2.71	4.27	160.94	38.21	0.80
UIL.21.13539	Fresh Laban (Alrawabi)	3.27	2.29	0.20	0.86	0.00	0.06	3.97	0.49	0.07	0.10	0.10	3.40	3.28	4.62	255.29	61.03	0.00
UIL.21.13541	Laban Up (Alsafwa)	1.55	1.04	0.11	0.39	0.00	340.60	3.89	1.71	0.10	0.10	0.10	2.20	2.60	3.96	168.87	40.19	0.87
UIL.21.13540	Mazoon Laban Up	0.89	0.60	0.05	0.23	0.00	345.30	3.70	1.30	0.10	0.10	0.10	2.40	1.92	3.70	128.47	30.49	0.88
UIL.21.17409	Activia Full Fat Plain Yoghurt	2.08	1.53	0.12	0.43	0.00	34.91	3.32	0.28	0.07	0.07	0.07	3.04	2.77	3.75	187.69	44.77	0.09
UIL.21.14131	Cream (Luna)	21.71	16.71	0.75	4.69	0.00	0.07	3.10	0.10	0.10	0.10	0.10	3.10	2.03	3.97	905.27	219.39	0.00
UIL.21.13546	Alrawabi Up:Laban Drink	1.79	1.20	0.11	0.47	0.01	0.32	3.04	1.10	0.10	0.10	0.10	1.93	2.15	3.23	157.69	37.63	0.00
UIL.21.13549	Laban Drink (Unikai)	0.74	0.48	0.04	0.19	0.00	1875.60	3.02	0.00	0.00	0.00	0.00	3.02	3.19	3.29	137.54	32.58	4.76
UIL.21.14180	Mazoon Sambharam Spicy Laban Drink	0.85	0.54	0.05	0.26	0.00	355.00	1.68	0.08	0.10	0.10	0.10	1.60	1.94	3.33	121.04	28.73	0.90
UIL.21.14179	Laban Drinks (Alkhamayil)	0.58	0.37	0.03	0.17	0.00	0.00	0.89	0.10	0.10	0.10	0.10	0.89	1.94	4.22	126.18	29.86	0.00
UIL.21.17404	Balade Farms Ayran Original Laban	1.27	1.02	0.20	0.05	0.00	375.00	0.00	0.00	0.00	0.00	0.00	0.00	1.65	3.40	132.84	31.63	0.95
UIL.21.14553	Cream (Puck)	24.51	11.62	3.46	9.30	0.13	2.65	0.00	0.00	0.00	0.00	0.00	0.00	2.65	2.88	1000.88	242.71	0.01
UIL.21.13830	Nestle Cream	23.44	13.40	1.64	8.40	0.00	0.08	0.00	0.00	0.00	0.00	0.00	0.00	3.09	4.90	1003.11	242.92	0.00

Sample ID (from lab)	Name of Product & Brand	Amounts per 100 g/ml from Laboratory Analysis														Amounts per 100 g/ml calculated		
		Total Fat (g)	SFA (g)	PUFA (g)	MUFA (g)	TFA (g)	Sodium (mg)	Total Sugars (g)	Glucose (g)	Fructose (g)	Sucrose (g)	Maltosa (g)	Lactose (g)	Protein (g)	Carbo-hydrate (g)	Energy (kJ)	Energy (kcal)	Salt (g)
UIL.21.14572	Chedder Cheese (Almarai)	24.97	16.77	2.53	5.67	0.00	1898.77	0.00	0.00	0.00	0.00	0.00	0.00	17.90	0.00	1228.07	296.30	4.82
UIL.21.13835	Haloumi Pinar	25.87	14.12	4.52	7.23	0.00	1896.30	0.00	0.00	0.00	0.00	0.00	0.00	24.32	3.95	1437.78	345.91	4.82
UIL.21.14571	Chedder Cheese (Kraft)	25.56	17.84	2.56	4.24	0.96	1800.00	0.00	0.00	0.00	0.00	0.00	0.00	13.56	5.64	1272.12	306.84	4.57
UIL.21.13840	Almarai Burger Slice Cheese	25.30	15.15	2.80	7.35	0.01	1378.00	0.00	0.00	0.00	0.00	0.00	0.00	15.20	5.75	1292.25	311.50	3.50
UIL.21.13839	Kraft Slice Cheese Original	21.56	13.17	0.84	6.91	0.65	1356.20	0.00	0.00	0.00	0.00	0.00	0.00	15.14	7.43	1181.41	284.32	3.44
UIL.21.13834	Haloumi Cheese Al-Marai	27.10	24.20	0.60	2.30	0.01	1254.00	0.00	0.00	0.00	0.00	0.00	0.00	23.87	3.53	1468.50	353.50	3.19
UIL.21.13848	Kraft Cheddar Cheese Spread Original	28.21	19.12	3.56	3.71	1.82	1215.60	0.00	0.00	0.00	0.00	0.00	0.00	8.34	4.21	1257.12	304.09	3.09
UIL.21.13844	Almarai Cream Cheese Cheddar	30.87	15.50	3.93	11.43	0.01	1152.00	0.00	0.00	0.00	0.00	0.00	0.00	10.23	4.53	1393.10	336.87	2.93
UIL.21.13838	Cheese Triangles Puck	25.52	12.95	7.30	5.15	0.12	1098.60	0.00	0.00	0.00	0.00	0.00	0.00	8.47	5.83	1187.34	286.88	2.79
UIL.21.13837	Cheese Triangles Al Maraai	27.30	17.87	5.20	4.23	0.01	952.00	0.00	0.00	0.00	0.00	0.00	0.00	10.37	3.50	1245.83	301.17	2.42
UIL.21.13847	Puck Cream Cheese	31.85	20.97	2.16	7.72	1.00	836.20	0.00	0.00	0.00	0.00	0.00	0.00	8.65	2.49	1367.83	331.21	2.12
UIL.21.13846	Puck Cream Cheese Cheddar	32.01	19.20	3.00	6.83	0.98	795.60	0.00	0.00	0.00	0.00	0.00	0.00	8.52	2.62	1373.75	332.65	2.02
UIL.21.13836	Triangle Cheese Abu Alwald	21.02	11.92	4.14	4.64	0.32	745.30	0.00	0.00	0.00	0.00	0.00	0.00	8.22	8.11	1055.35	254.50	1.89
UIL.21.13845	Almarai Cream Cheese	34.10	25.13	0.93	6.77	1.30	745.00	0.00	0.00	0.00	0.00	0.00	0.00	5.20	4.27	1422.63	344.77	1.89
UIL.21.14554	Pinar Processed Cream Cheese Spread	31.02	27.80	1.13	1.96	0.12	709.36	0.00	0.00	0.00	0.00	0.00	0.00	6.66	1.61	1288.33	312.26	1.80
UIL.21.13841	Puck Slice Cheese Original	27.66	14.04	3.35	10.27	0.00	608.50	0.00	0.00	0.00	0.00	0.00	0.00	14.81	5.68	1371.75	330.90	1.55
UIL.21.13843	Mazoon Spread Cheese	33.48	24.12	1.48	7.88	0.00	395.90	0.00	0.00	0.00	0.00	0.00	0.00	6.72	3.08	1405.36	340.52	1.01
UIL.21.13842	Mazoon Spread Cheese Cheddar	33.52	23.82	1.49	0.58	0.00	395.20	0.00	0.00	0.00	0.00	0.00	0.00	7.12	2.36	1401.40	339.60	1.00
UIL.21.13456	Britannia Toastea Wheat Rusk	9.74	4.35	1.52	3.90	0.01	223.23	26.71	9.84	9.90	6.94	0.00	0.00	9.61	77.48	1841.10	436.06	0.57
UIL.21.13455	Tiffany Whole Wheat Rusks	8.49	3.80	1.28	3.37	0.00	225.20	23.11	4.93	5.08	13.10	0.00	0.00	10.13	76.93	1794.15	424.65	0.57
UIL.21.13429	Indomie Fried Noodles	20.92	11.35	2.68	6.89	0.00	1364.30	8.24	3.56	0.66	4.02	0.10	0.10	8.54	62.06	1974.24	470.68	3.47
UIL.21.14573	Family Corned Beef	10.59	6.54	0.23	3.82	0.00	425.60	4.02	3.41	0.10	0.61	0.10	0.10	24.87	5.10	901.32	215.19	1.08
UIL.21.14202	Al-Islami Ground Beef	9.25	4.16	1.54	3.55	0.00	420.50	1.38	1.08	0.18	0.10	0.12	0.10	11.74	2.34	581.61	139.57	1.07

Sample ID (from lab)	Name of Product & Brand	Amounts per 100 g/ml from Laboratory Analysis														Amounts per 100 g/ml calculated		
		Total Fat (g)	SFA (g)	PUFA (g)	MUFA (g)	TFA (g)	Sodium (mg)	Total Sugars (g)	Glucose (g)	Fructose (g)	Sucrose (g)	Maltosa (g)	Lactose (g)	Protein (g)	Carbo-hydrate (g)	Energy (kJ)	Energy (kcal)	Salt (g)
UIL.21.14201	Corned Beef (Taybat)	8.42	4.14	0.84	3.44	0.00	211.20	0.54	0.32	0.16	0.20	0.06	0.20	12.50	12.78	741.30	176.90	0.54
UIL.21.14205	Al-Safa Chicken Franks	14.88	8.06	0.35	6.47	0.01	1301.06	0.00	0.00	0.00	0.00	0.00	0.00	12.29	5.85	859.15	206.53	3.30
UIL.21.14200	Beef Burger (Taybat)	13.52	6.99	1.43	5.10	0.00	620.63	0.00	0.00	0.00	0.00	0.00	0.00	19.11	6.40	933.91	223.72	1.58
UIL.21.14204	Americana Beef Hotdog	12.56	5.27	5.94	1.35	0.00	603.21	0.00	0.00	0.00	0.00	0.00	0.00	15.05	2.11	756.44	181.68	1.53
UIL.21.14198	Sadia Chicken Burger	15.24	5.93	4.18	5.13	0.00	595.00	0.00	0.00	0.00	0.00	0.00	0.00	14.21	6.78	920.71	221.12	1.51
UIL.21.14203	Al-Kabeer Jumbo Hotdogs	7.69	4.31	2.93	0.24	0.21	539.62	0.00	0.00	0.00	0.00	0.00	0.00	17.12	4.25	647.82	154.69	1.37
UIL.21.14194	Al-Safa Breaded Chicken Finger	8.31	2.56	2.33	3.42	0.00	485.60	0.00	0.00	0.00	0.00	0.00	0.00	18.80	19.68	961.63	228.71	1.23
UIL.21.14206	Sadia Chicken Franks	15.65	6.77	0.65	8.23	0.00	461.36	0.00	0.00	0.00	0.00	0.00	0.00	12.94	2.70	844.93	203.41	1.17
UIL.21.14199	Beef Burger Sadia	20.57	10.32	5.62	4.63	0.00	455.60	0.00	0.00	0.00	0.00	0.00	0.00	14.21	6.70	1116.56	268.77	1.16
UIL.21.14195	Sadia Chicken Nuggets Crispy	10.35	3.21	2.86	4.28	0.00	415.70	0.00	0.00	0.00	0.00	0.00	0.00	11.89	20.87	939.87	224.19	1.06
UIL.21.14196	Al-Safa Chicken Burger	10.41	3.01	2.85	4.55	0.00	395.20	0.00	0.00	0.00	0.00	0.00	0.00	16.86	3.94	738.77	176.89	1.00
UIL.21.13292	Tuna Alalali In Water	0.01	0.00	0.00	0.00	0.00	479.50	0.89	0.89	0.10	0.10	0.10	0.10	23.19	3.29	450.53	106.01	1.22
UIL.21.13294	Skipjack Tuna California Garden In Sun Flower Oil	2.98	0.36	1.90	0.71	0.00	303.20	0.89	0.24	0.10	0.10	0.10	0.10	25.06	1.99	570.11	135.02	0.77
UIL.21.13295	Light Chunks Tuna California Garden In Water	1.07	0.80	0.24	0.03	0.00	215.30	0.84	0.84	0.10	0.10	0.10	0.10	23.69	0.95	458.47	108.19	0.55
UIL.21.13291	Skipjack Tuna Alalali In Sun Flower Oil	11.02	1.46	2.65	6.92	0.00	480.20	0.80	0.10	0.10	0.10	0.10	0.80	23.12	3.96	868.10	207.50	1.22
UIL.21.13293	White Tuna Meat In Sun Flower Oil Delmonte	5.58	0.80	3.04	1.72	0.00	425.20	0.36	0.36	0.10	0.10	0.10	0.10	24.75	2.96	677.53	161.06	1.08
UIL.21.13434	White Tuna Meat In Brine	0.61	0.46	0.03	0.13	0.00	380.70	0.10	0.10	0.10	0.10	0.10	0.10	25.57	1.97	490.75	115.65	0.97
UIL.21.14397	Diamond Strawberry Jam	0.31	0.27	0.00	0.04	0.00	15.20	63.84	33.03	15.93	5.88	9.00	0.10	0.39	66.47	1148.09	270.23	0.04
UIL.21.14405	Mango Thokku Pickle In Oil / Priya	4.21	1.03	1.53	1.66	0.00	4470.50	22.40	19.70	2.80	0.10	0.10	0.10	4.07	28.04	701.64	166.33	11.36
UIL.21.14404	Tomato Paste / Alalali	0.72	0.38	0.27	0.07	0.00	410.50	12.30	11.63	0.68	0.10	0.10	0.10	3.54	15.21	345.39	81.48	1.04
UIL.21.13278	Baked Beans California Garden	1.20	0.38	0.11	0.71	0.00	312.88	10.40	1.16	1.18	8.06	0.08	0.08	5.20	12.42	343.86	81.26	0.79
UIL.21.14399	Tomato Paste / Almudhish	1.02	0.39	0.56	0.06	0.00	0.43	10.40	5.00	5.30	0.10	0.10	0.10	5.15	16.84	411.57	97.14	0.00

Sample ID (from lab)	Name of Product & Brand	Amounts per 100 g/ml from Laboratory Analysis														Amounts per 100 g/ml calculated		
		Total Fat (g)	SFA (g)	PUFA (g)	MUFA (g)	TFA (g)	Sodium (mg)	Total Sugars (g)	Glucose (g)	Fructose (g)	Sucrose (g)	Maltosa (g)	Lactose (g)	Protein (g)	Carbo-hydrate (g)	Energy (kJ)	Energy (kcal)	Salt (g)
UIL.21.13276	Green Giant Sweet Corn	1.63	0.38	0.81	0.44	0.00	203.50	7.48	0.46	0.19	6.83	0.13	0.13	2.01	15.16	352.10	83.33	0.52
UIL.21.13290	Sweet Corn American Garden	1.29	0.29	0.40	0.60	0.00	178.56	6.41	0.30	0.14	5.96	0.08	0.08	2.50	17.23	383.17	90.54	0.45
UIL.21.13282	Baked Beans Almazraa	0.63	0.16	0.08	0.04	0.00	392.46	6.12	0.74	0.85	4.53	0.08	0.08	4.40	25.97	539.62	127.15	1.00
UIL.21.14400	Tomato Paste / Jumbo	0.65	0.25	0.36	0.04	0.00	301.20	4.14	1.32	2.20	0.62	0.10	0.10	2.09	17.76	361.50	85.25	0.77
UIL.21.14556	LUNA Baked Beans In Tomato Sauce	0.30	0.19	0.07	0.04	0.00	320.00	3.96	0.72	1.06	2.18	0.00	0.00	7.07	14.44	376.77	88.74	0.81
UIL.21.13280	Sweet Corn California Garden	1.08	0.28	0.37	0.42	0.00	132.69	3.08	0.08	0.08	3.08	0.08	0.08	2.15	19.98	416.07	98.22	0.34
UIL.21.14559	Peas Processed	0.01	0.00	0.00	0.00	0.00	214.70	2.46	0.48	0.08	1.43	0.08	0.08	3.99	17.19	360.42	84.81	0.55
UIL.21.14555	La Ming Beans In Tomato Sauce	0.98	0.23	0.54	0.21	0.00	0.00	2.40	0.45	0.77	1.17	0.10	0.10	7.64	88.34	1667.92	392.74	0.00
UIL.21.13288	Sweet Corn Libbys	0.55	0.16	0.20	0.20	0.00	222.80	2.09	0.79	0.08	0.90	0.08	0.08	1.68	9.59	212.05	50.06	0.57
UIL.21.13279	Processed Peas California Garden	0.01	0.15	0.00	0.00	0.00	329.31	1.64	1.42	0.22	0.08	0.08	0.08	4.59	16.37	356.63	83.92	0.84
UIL.21.13287	Processed Peas Almazraa	0.01	0.00	0.00	0.00	0.00	325.54	1.64	0.28	0.32	1.05	0.08	0.08	4.48	15.42	338.58	79.67	0.83
UIL.21.14557	Foul Medammas (American Garden)	0.01	0.00	0.00	0.00	0.00	340.98	1.22	0.78	0.08	0.45	0.08	0.08	6.83	16.53	397.43	93.52	0.87
UIL.21.14558	Lunafoul Medames Extra Grade Fava Beans	0.41	0.21	0.20	0.00	0.00	330.00	1.17	0.80	0.10	0.16	0.21	0.10	7.30	16.68	422.83	99.61	0.84
UIL.21.13277	Plain Medammes California Garden	0.01	0.00	0.00	0.00	0.00	342.62	0.93	0.43	0.08	0.50	0.08	0.00	6.02	17.51	400.31	94.19	0.87
UIL.21.13281	Chick Peas California Garden	0.64	0.13	0.23	0.28	0.00	266.54	0.84	0.22	0.08	0.62	0.08	0.08	7.12	19.98	484.32	114.15	0.68
UIL.21.13286	Chick Peas Almazraa	0.61	0.11	0.17	0.32	0.00	294.15	0.81	0.08	0.08	0.81	0.08	0.08	6.04	16.14	399.49	94.18	0.75
UIL.21.13283	Foul Medamas Almazraa	0.01	0.00	0.00	0.00	0.00	434.00	0.77	0.67	0.08	0.08	0.08	0.08	5.18	15.11	345.12	81.21	1.10
UIL.21.13284	Chick Peas Luna	1.60	0.74	0.21	0.65	0.00	346.00	0.76	0.39	0.10	0.36	0.01	0.01	4.71	16.44	418.75	99.00	0.88
UIL.21.13289	Boildchick Peas Mara	1.31	0.26	0.37	0.68	0.00	153.50	0.74	0.10	0.10	0.74	0.10	0.10	4.11	11.37	311.63	73.71	0.39
UIL.21.13285	Green Peas Luna	0.42	0.12	0.11	0.19	0.00	237.00	0.61	0.13	0.10	0.49	0.10	0.10	4.27	16.41	367.10	86.50	0.60
UIL.21.14403	Mango Pickles In Oil / Ahmed	20.80	2.30	6.80	11.70	0.01	3016.05	0.50	0.50	0.50	0.50	0.50	0.50	3.05	9.35	980.40	236.80	7.66
UIL.21.14406	Mango Pickle / Aeroplane	4.08	1.23	2.19	0.66	0.00	5040.00	0.10	0.10	0.10	0.10	0.10	0.10	1.46	9.62	339.32	81.04	12.80
UIL.21.14394	American Garden BBQ Sauce Original	0.03	0.01	0.01	0.01	0.01	989.67	34.14	17.75	15.58	0.83	0.11	0.28	0.03	41.64	709.36	166.92	2.51

Sample ID (from lab)	Name of Product & Brand	Amounts per 100 g/ml from Laboratory Analysis													Amounts per 100 g/ml calculated		
		Total Fat (g)	SFA (g)	PUFA (g)	MUFA (g)	TFA (g)	Sodium (mg)	Total Sugars (g)	Glucose (g)	Fructose (g)	Sucrose (g)	Maltosa (g)	Lactose (g)	Protein (g)	Carbo-hydrate (g)	Energy (kJ)	Energy (kcal)	Salt (g)
UIL.21.14395	Heinz Classic BBQ Smokey And Rich	0.35	0.15	0.15	0.05	0.00	495.60	28.68	6.09	6.28	16.31	0.10	0.10	0.85	33.77	601.49	141.63	1.26
UIL.21.14411	Tomato Ketchup/ Jumbo	0.31	0.19	0.08	0.03	0.00	163.52	26.94	8.59	9.35	9.00	0.10	0.10	0.99	29.35	527.25	124.15	0.42
UIL.21.14413	Tomato Ketchup / Heinz	0.01	0.00	0.00	0.00	0.00	721.49	22.58	5.81	6.54	10.23	0.10	0.10	1.40	23.30	420.27	98.89	1.83
UIL.21.14408	Tomato Ketchup / Hayat	0.07	0.01	0.01	0.01	0.01	815.93	22.20	7.07	7.33	0.67	7.73	0.67	0.60	26.60	464.87	109.40	2.07
UIL.21.14392	Delicio French Dressing	36.86	6.64	23.00	7.21	0.01	1117.21	19.79	5.57	1.14	13.14	0.71	0.71	1.07	15.50	1645.43	398.00	2.84
UIL.21.14393	Delicio 1000 Island	36.93	6.64	23.21	7.07	0.01	1040.36	14.29	6.86	2.43	5.07	0.71	0.71	0.86	9.64	1544.86	374.36	2.64
UIL.21.14402	Hot Sauce / Excellence	0.01	0.00	0.00	0.00	0.00	2289.60	9.79	7.39	0.29	2.11	0.10	0.10	0.33	10.62	186.52	43.89	5.82
UIL.21.13430	Maggi Chicken Stock	21.89	12.87	2.05	6.95	0.02	18220.00	8.23	3.56	0.66	4.02	0.00	0.00	5.16	14.51	1144.32	275.69	46.28
UIL.21.14407	Pizza Sauce Olive& Mushrooms / Alalali	1.58	0.36	0.17	1.05	0.00	358.20	6.38	2.65	3.10	0.62	0.10	0.10	2.05	9.65	257.36	61.02	0.91
UIL.21.14415	Classic Mayonnaise / Mazola	79.21	13.64	43.57	22.00	0.01	498.43	3.71	0.86	0.79	0.71	2.14	0.71	0.07	3.57	2992.86	727.50	1.27
UIL.21.14416	Creamy Classic Mayonnaise / Heinz	35.85	7.32	20.44	8.09	0.00	807.17	3.46	0.62	0.62	2.21	0.10	0.10	0.07	12.14	1534.02	371.49	2.05
UIL.21.14401	Tabasco Hot Sauce	0.01	0.00	0.00	0.00	0.00	690.32	2.65	0.85	0.10	0.10	1.80	0.10	1.59	2.84	75.68	17.81	1.75
UIL.21.14398	Soy Sauce Classic / American Garden	0.20	0.04	0.04	0.04	0.04	2853.00	2.00	2.00	2.00	2.00	2.00	2.00	0.60	2.00	51.60	12.20	7.25
UIL.21.14417	Hot Sause / American Garden	0.20	0.04	0.04	0.04	0.04	2790.00	2.00	2.00	2.00	2.00	2.00	2.00	0.60	2.00	51.60	12.20	7.09
UIL.21.13296	Hayat Hot Sauce	0.70	0.00	0.00	0.00	0.00	2615.90	0.72	0.72	0.10	0.10	0.10	0.10	0.63	0.77	49.70	11.90	6.64
UIL.21.13297	Jumbo Hot Sauce	0.10	0.00	0.00	0.00	0.00	1610.20	0.58	0.58	0.10	0.10	0.10	0.10	0.34	0.86	24.10	5.70	4.09
UIL.21.14564	Maggi Beef Flavour	0.00	0.00	0.00	0.00	0.00	19900.00	0.00	0.00	0.00	0.00	0.00	0.00	0.00	12.94	219.98	51.76	50.55
UIL.21.14565	Maggi Chicken Stock Less Salt	0.00	0.00	0.00	0.00	0.00	14780.00	0.00	0.00	0.00	0.00	0.00	0.00	0.00	29.10	494.70	116.40	37.54
UIL.21.14561	Knour CHICKEN Stock	0.00	0.00	0.00	0.00	0.00	539.21	0.00	0.00	0.00	0.00	0.00	0.00	0.31	0.37	11.56	2.72	1.37
UIL.21.14562	Knour Beef Flavoured Stock	0.00	0.00	0.00	0.00	0.00	460.63	0.00	0.00	0.00	0.00	0.00	0.00	0.25	0.52	13.09	3.08	1.17
UIL.21.14563	Knor Vegetable Stock Cubes	0.00	0.00	0.00	0.00	0.00	442.89	0.00	0.00	0.00	0.00	0.00	0.00	0.29	0.44	12.41	2.92	1.12
UIL.21.17402	Tang (Orange) (Powder)	0.00	0.00	0.00	0.00	0.00	180.92	96.92	0.40	0.40	96.92	0.40	0.40	0.04	96.92	1648.32	387.84	0.46
UIL.21.13850	Al Deyafa Orang Flavour (Powder)	0.00	0.00	0.00	0.00	0.00	0.17	94.69	0.10	0.10	94.96	0.10	0.10	0.01	95.73	1627.58	382.96	0.00
UIL.21.13828	Al Deyafa Fruit Cordial	0.00	0.00	0.00	0.00	0.00	15.20	60.59	31.86	28.73	0.10	0.10	0.10	0.00	64.33	1093.61	257.32	0.04

Sample ID (from lab)	Name of Product & Brand	Amounts per 100 g/ml from Laboratory Analysis														Amounts per 100 g/ml calculated		
		Total Fat (g)	SFA (g)	PUFA (g)	MUFA (g)	TFA (g)	Sodium (mg)	Total Sugars (g)	Glucose (g)	Fructose (g)	Sucrose (g)	Maltosa (g)	Lactose (g)	Protein (g)	Carbo-hydrate (g)	Energy (kJ)	Energy (kcal)	Salt (g)
UIL.21.14138	Al Rawabi Red Grape Juice	0.10	0.00	0.00	0.00	0.00	0.00	13.96	6.95	7.01	0.10	0.10	0.10	0.25	15.18	266.01	62.62	0.00
UIL.21.14135	Almarai Graps And Berries (Farms Select)	0.05	0.00	0.00	0.00	0.00	0.00	13.61	2.04	2.63	8.94	0.05	0.05	0.26	14.08	245.63	57.81	0.00
UIL.21.13829	Mango Juice Mazoon 200 ml	0.00	0.00	0.00	0.00	0.00	0.00	13.44	0.10	0.71	12.63	0.10	0.10	0.00	16.44	279.48	65.76	0.00
UIL.21.13827	Natural Mango Asafwa 200 ml	0.00	0.00	0.00	0.00	0.00	5.30	13.13	0.39	0.99	11.75	0.10	0.10	0.00	13.74	233.58	54.96	0.01
UIL.21.14142	Fruit Nectar (Lacnor)	0.10	0.00	0.00	0.00	0.00	1.62	12.73	2.74	3.42	6.57	0.10	0.10	0.09	13.20	229.63	54.06	0.00
UIL.21.14132	Al Rabie Fruit Cocktail Nectar	0.00	0.00	0.00	0.00	0.00	0.00	12.47	3.47	4.26	4.73	0.10	0.10	0.00	15.49	263.33	61.96	0.00
UIL.21.13851	Vimto	0.00	0.00	0.00	0.00	0.00	0.00	12.39	6.59	5.80	0.04	0.12	0.15	0.00	12.58	213.86	50.32	0.00
UIL.21.14140	Sun Top Orange Fruit Drink	0.10	0.00	0.00	0.00	0.00	3.50	11.84	5.59	6.25	0.10	0.10	0.10	0.35	12.67	225.04	52.98	0.01
UIL.21.14134	Al Marai Mango Juice	0.10	0.00	0.00	0.00	0.00	0.00	11.81	0.10	0.10	11.81	0.10	0.10	0.38	15.40	271.96	64.02	0.00
UIL.21.13824	Mixed Fruit Juice (Mazoon)	0.00	0.00	0.00	0.00	0.00	0.00	11.77	5.59	5.72	0.46	0.10	0.10	0.00	13.24	225.08	52.96	0.00
UIL.21.14143	Fruit Drink (Orange) Rani	0.10	0.00	0.00	0.00	0.00	7.60	11.56	4.46	4.71	2.38	0.10	0.10	0.16	13.13	229.63	54.06	0.02
UIL.21.14139	A'safwah Mixed Fruit Juice	0.10	0.00	0.00	0.00	0.00	0.00	11.56	0.24	0.47	10.85	0.10	0.10	0.08	14.07	244.25	57.50	0.00
UIL.21.14141	Orange Drink (Top Fruit)	0.10	0.00	0.00	0.00	0.00	5.60	10.72	0.23	2.80	7.92	0.10	0.10	0.09	11.92	207.87	48.94	0.01
UIL.21.14136	Nada Mango Juice	0.01	0.00	0.00	0.00	0.00	0.00	10.50	1.34	1.30	7.86	0.05	0.05	0.26	12.78	221.87	52.21	0.00
UIL.21.14137	Nada Orange Juice	0.05	0.00	0.00	0.00	0.00	0.00	10.46	2.00	2.61	5.85	0.05	0.05	0.70	10.27	188.34	44.33	0.00
UIL.21.13821	Asafwa Natural Orange 200 ml	0.00	0.00	0.00	0.00	0.00	165.20	10.25	2.07	2.55	5.63	0.10	0.10	0.00	12.94	219.98	51.76	0.42
UIL.21.13822	Almarai Orange Juice 100% 300 ml	0.00	0.00	0.00	0.00	0.00	0.00	9.39	2.40	2.45	4.54	0.03	0.03	2.40	9.59	203.77	47.95	0.00
UIL.21.13825	Top fruit. Fruit Cocktail 200 ml	0.00	0.00	0.00	0.00	0.00	23.00	9.28	4.38	4.90	0.10	0.10	0.10	0.00	11.38	193.46	45.52	0.06
UIL.21.14133	Al Rawabi Apple Juice	0.10	0.02	0.00	0.00	0.00	0.00	9.15	5.81	3.32	0.02	0.00	0.00	0.26	10.35	184.07	43.34	0.00
UIL.21.13826	Orange No Add Sugar (Top Fruit)	0.00	0.00	0.00	0.00	0.00	8.50	9.14	2.83	3.05	3.26	0.10	0.10	0.00	10.45	177.65	41.80	0.02
UIL.21.13823	Alrawabi Orange 1005juice	0.00	0.00	0.00	0.00	0.00	60.10	6.65	1.40	1.43	3.82	0.10	0.10	0.00	11.00	187.00	44.00	0.15
UIL.21.13450	Galaxy Smooth Milk	0.38	0.08	0.08	0.08	0.08	0.04	73.08	3.85	3.85	57.69	3.85	3.85	3.85	92.31	1648.85	388.08	0.00
UIL.21.14569	Milk Powder (Majan)	29.21	20.19	3.33	5.69	0.00	329.74	47.83	1.83	0.10	0.12	0.10	45.88	24.10	40.99	2187.30	523.25	0.84

Sample ID (from lab)	Name of Product & Brand	Amounts per 100 g/ml from Laboratory Analysis														Amounts per 100 g/ml calculated		
		Total Fat (g)	SFA (g)	PUFA (g)	MUFA (g)	TFA (g)	Sodium (mg)	Total Sugars (g)	Glucose (g)	Fructose (g)	Sucrose (g)	Maltosa (g)	Lactose (g)	Protein (g)	Carbo-hydrate (g)	Energy (kJ)	Energy (kcal)	Salt (g)
UIL.21.14570	Milk Powder (Almudhish)	29.52	19.24	3.10	6.49	0.70	280.95	38.95	0.10	0.10	0.10	0.10	38.95	24.88	40.39	2201.83	526.76	0.71
UIL.21.13552	Strwaberry Milk (Asafwah)	2.42	1.78	0.12	0.50	0.00	58.30	14.14	0.10	0.10	7.93	0.00	6.21	3.42	14.15	388.23	92.06	0.15
UIL.21.13553	Strawberry Flavored Milk	2.50	1.74	0.17	0.58	0.00	58.08	12.03	0.04	0.04	6.98	0.04	5.05	3.04	12.13	350.50	83.21	0.15
UIL.21.13554	Strwberry Fresh Milk (Alrawabi)	3.28	2.32	0.15	0.79	0.00	150.60	11.77	0.00	0.00	7.09	0.00	4.68	4.05	11.45	385.01	91.56	0.38
UIL.21.14171	Strawberry Flavored Milk (Lacnor)	3.26	1.94	0.41	0.91	0.00	69.50	9.84	0.10	0.10	5.06	0.10	4.78	2.82	10.96	354.88	84.46	0.18
UIL.21.14170	Strawberry Milk (Nada)	3.21	2.27	0.16	0.78	0.00	48.90	9.67	0.10	0.10	5.75	0.10	3.92	2.18	11.68	354.39	84.33	0.12
UIL.21.14129	Evaporated Milk (Rainbow)	8.85	6.25	0.60	1.94	0.06	105.30	9.54	0.10	0.10	0.10	0.10	9.54	5.94	10.57	608.12	145.69	0.27
UIL.21.14130	Tea Milk (Almudhish)	8.12	4.05	1.22	2.83	0.02	105.60	9.28	0.10	0.10	0.10	0.10	9.28	5.49	10.46	571.59	136.88	0.27
UIL.21.13551	Mazoon Fresh Milk Strawberry Flavour	3.21	2.36	0.16	0.68	0.01	54.20	6.47	0.10	0.10	3.47	0.10	3.00	3.07	10.75	353.71	84.17	0.14
UIL.21.13849	Power Horse Energy	0.00	0.00	0.00	0.00	0.00	66.24	11.28	5.84	3.44	2.00	0.04	0.04	0.04	11.82	201.55	47.42	0.17
UIL.21.14173	Energy Drink (Red Bull)	0.01	0.00	0.00	0.00	0.00	45.20	10.15	4.18	2.41	3.56	0.10	0.10	0.01	11.58	197.40	46.45	0.11
UIL.21.14178	Vitaene Extra (Pokka)	0.01	0.00	0.00	0.00	0.00	0.00	13.36	4.90	7.95	0.08	0.24	0.27	0.01	14.78	251.63	59.21	0.00
UIL.21.14176	Mountain Dew	0.01	0.00	0.00	0.00	0.00	15.60	11.55	4.03	4.28	3.24	0.10	0.10	0.01	12.31	209.81	49.37	0.04
UIL.21.14175	Pepsi	0.01	0.00	0.00	0.00	0.00	10.40	9.98	4.96	4.94	0.09	0.10	0.10	0.01	10.20	173.94	40.93	0.03
UIL.21.14174	Coca Cola	0.01	0.00	0.00	0.00	0.00	15.30	9.78	4.59	4.69	0.50	0.10	0.10	0.01	10.95	186.69	43.93	0.04
UIL.21.14172	Lipton Peach Ice Tea	0.01	0.00	0.00	0.00	0.00	10.60	4.40	1.16	1.52	1.72	0.10	0.10	0.10	4.51	78.74	18.53	0.03
UIL.21.14177	Malt Beverage (Barbican)	0.01	0.00	0.00	0.00	0.00	10.20	2.22	0.34	0.13	0.75	1.00	0.10	0.26	2.97	55.28	13.01	0.03

Table S5: Creation of Front of Pack (FoP) nutrition labelling in units of mass and units per portion.

Sr.No	Sample ID (from lab)	Name of Product & Brand	Portion (g/ml)	Nutrient Profile Group	Food Item	Energy (kJ per 100 g)	Energy (kcal per 100 g)	Fat (g per 100g)	Fat (g per serving)	FINAL FAT	SFA[1] (g per 100g)	SFA[1] (g per serving)	FINAL SFA[1]	Sugars (g per 100g)	Sugars (g per serving)	FINAL Sugars	Salt (g per 100g)	Salt (g per serving)	FINAL Salt
17 J	UIL.21.14414	Natural Honey / Alshifa	100	0	Food	1421.88	334.56	0.00	0.00		0.00	0.00		80.30	80.30		0.00	0.00	
15 J	UIL.21.14412	Pure honey / Capilano	100	0	Food	1392.98	327.76	0.00	0.00		0.00	0.00		81.00	81.00		0.00	0.00	
FA19	UIL.21.13442	Batook Specialmint Chewing Gum	2.5	1	Food	1572.00	370.00	0.40	0.01		0.08	0.00		2.00	0.05		0.00	0.00	
FA16	UIL.21.13439	Bounty Minis	28.5	1	Food	1843.66	438.02	14.34	4.09		8.38	2.39		15.02	4.28		0.82	0.23	
SI3	UIL.21.13560	Cream Caramel Almaraei	100	1	Food	524.34	124.03	2.23	2.23		1.66	1.66		20.92	20.92		0.15	0.15	
13 J	UIL.21.14410	Custard Powder / Alalali	100	1	Food	1543.29	363.13	0.01	0.01		0.00	0.00		0.10	0.10		0.21	0.21	
J12	UIL.21.14409	Custard Powder/Tiara	100	1	Food	1509.10	355.15	0.23	0.23		0.14	0.14		0.21	0.21		0.00	0.00	
A15	UIL.21.14396	Daily Fresh/ Custard Powder	100	1	Food	1444.20	340.00	0.64	0.64		0.21	0.21		3.07	3.07		1.78	1.78	
FA49	UIL.21.13550	Danet Cream Caramel	100	1	Food	486.20	115.05	2.21	2.21		1.59	1.59		17.80	17.80		0.16	0.16	
FA18	UIL.21.13441	Extra Peppermint	1.9	1	Food	2166.84	510.00	0.53	0.01		0.11	0.00		52.63	1.00		0.00	0.00	
FA12	UIL.21.13435	Kinder Joy	100	1	Food	2279.29	545.78	32.22	32.22		15.15	15.15		49.62	49.62		0.49	0.49	
FA14	UIL.21.13437	Kitkat	100	1	Food	2157.18	515.01	25.29	25.29		13.76	13.76		45.37	45.37		0.19	0.19	
FA17	UIL.21.13440	Mars Minis	13	1	Food	1989.15	473.31	17.92	2.33		11.54	1.50		57.77	7.51		0.56	0.07	
FA21	UIL.21.13444	Mentos Sugar free Chewing Gum	1.75	1	Food	1624.00	382.29	0.57	0.01		0.11	0.00		32.00	0.56		0.00	0.00	
A10	UIL.21.14391	Nutella Hazelnut Spread With Cocoa	100	1	Food	2282.86	546.32	31.20	31.20		10.53	10.53		57.94	57.94		0.11	0.11	
FA15	UIL.21.13438	Snickers Minis	15	1	Food	2130.60	509.20	26.80	4.02		6.73	1.01		52.53	7.88		0.67	0.10	
H10	UIL.21.13454	Twix Minis	20	1	Food	2192.55	523.35	25.35	5.07		11.45	2.29		48.60	9.72		0.47	0.09	
FA20	UIL.21.13443	Wrigleys Doublemint Chewing Gum	2.6	1	Food	1583.46	372.69	0.38	0.01		0.08	0.00		81.54	2.12		0.00	0.00	
A3	UIL.21.14384	Al Mudhish Puffed Corn Cheese Balls	12	2	Food	2369.67	568.08	35.75	4.29		16.58	1.99		4.42	0.53		1.58	0.19	
FA10	UIL.21.13433	Alrifai (Mixed Nuts)	28	2	Food	2243.49	537.42	32.43	9.08		4.64	1.30		7.18	2.01		0.61	0.17	
FA8	UIL.21.13431	American Garden Popcorn Butter	33	2	Food	1972.12	471.21	24.42	8.06		10.91	3.60		0.30	0.10		2.50	0.83	
FA9	UIL.21.13432	Best Salted Mixed Nuts	100	2	Food	2669.29	643.33	51.89	51.89		8.31	8.31		4.28	4.28		0.85	0.85	
J25	UIL.21.17408	Cheese Balls (Pofak Oman)	15	2	Food	2437.67	583.67	34.33	5.15		7.67	1.15		7.47	1.12		3.92	0.59	
J53	UIL.21.14418	Cheese pastry (Dhahabi)	100	2	Food	2415.34	579.83	39.15	39.15		16.79	16.79		4.17	4.17		1.50	1.50	
J65	UIL.21.14551	Cheetos / Flamin Hot	100	2	Food	1585.62	378.54	18.54	18.54		4.74	4.74		3.85	3.85		0.78	0.78	
A1	UIL.21.13463	Cheez Balls (Mr. Krispy)	30	2	Food	2394.93	573.70	34.63	10.39		9.73	2.92		1.07	0.32		1.78	0.53	
A5	UIL.21.14386	chips Oman	100	2	Food	2355.38	565.61	38.77	38.77		16.89	16.89		0.00	0.00		1.28	1.28	
A2	UIL.21.14383	Lay's (Salt)	14	2	Food	2354.14	564.36	35.50	4.97		16.07	2.25		0.00	0.00		1.51	0.21	
A9	UIL.21.14390	Mani (Mixed Nuts)	100	2	Food	2589.53	623.81	49.33	49.33		7.92	7.92		8.55	8.55		0.80	0.80	
H14	UIL.21.13458	Nabil Cracker Salted Biscuits	100	2	Food	1995.78	475.26	19.26	19.26		13.50	13.50		20.04	20.04		2.54	2.54	
A8	UIL.21.14389	Popcorn Cheese / American Garden	33	2	Food	2202.39	528.27	34.21	11.29		15.67	5.17		0.00	0.00		2.51	0.83	
A4	UIL.21.14385	potato chips ready salted	28	2	Food	2343.21	561.96	36.11	10.11		15.75	4.41		0.00	0.00		1.36	0.38	
H18	UIL.21.13462	Pringles Original	100	2	Food	2303.16	551.77	33.49	33.49		3.53	3.53		1.05	1.05		1.15	1.15	
A7	UIL.21.14388	Shai (Mix Nut)	100	2	Food	2572.58	619.56	48.44	48.44		6.87	6.87		6.85	6.85		0.38	0.38	
J74	UIL.21.14560	Sohar Chips	100	2	Food	2285.15	547.20	32.36	32.36		14.31	14.31		0.00	0.00		2.41	2.41	

Sr.No	Sample ID (from lab)	Name of Product & Brand	Portion (g/ml)	Nutrient Profile Group	Food Item	Energy (kJ per 100 g)	Energy (kcal per 100 g)	Fat (g per 100g)	Fat (g per serving)	FINAL FAT	SFA[1] (g per 100g)	SFA[1] (g per serving)	FINAL SFA[1]	Sugars (g per 100g)	Sugars (g per serving)	FINAL Sugars	Salt (g per 100g)	Salt (g per serving)	FINAL Salt
J51	UIL.21.13833	vanilla ice cream (Igloo)	53	4	Food	824.38	197.25	11.13	5.90		6.68	3.54		18.60	9.86		0.17	0.09	
J52	UIL.21.13832	vanilla ice cream (Mazoon)	100	4	Food	569.36	136.22	7.66	7.66		4.46	4.46		12.37	12.37		0.12	0.12	
FA5	UIL.21.13300	KELLOGG'S Coco Pops	100	5	Food	1624.48	382.96	2.48	2.48		1.15	1.15		24.48	24.48		0.00	0.00	
A6	UIL.21.14387	Kellogg's special (cereal)	39	5	Food	1631.64	384.56	2.21	0.86		0.59	0.23		13.92	5.43		1.77	0.69	
FA4	UIL.21.13299	NESTLE Chocapic	100	5	Food	1721.17	406.39	4.79	4.79		1.50	1.50		24.95	24.95		0.26	0.26	
FA3	UIL.21.13298	Nestle Gold Corn Flakes	100	5	Food	1626.83	383.36	1.96	1.96		0.38	0.38		10.57	10.57		1.08	1.08	
A34	UIL.21.14574	7 days mini croissant	11	6	Food	1969.55	471.82	28.55	3.14		16.73	1.84		16.82	1.85		0.52	0.06	
H16	UIL.21.13460	Americana Premium Butter Cookies	100	6	Food	2053.55	489.15	20.27	20.27		9.16	9.16		29.40	29.40		0.29	0.29	
J57	UIL.21.14552	Apple pastry	100	6	Food	1513.98	360.86	15.74	15.74		8.37	8.37		16.32	16.32		0.03	0.03	
J56	UIL.21.17405	Apple puff (Lusine)	70	6	Food	1450.54	345.16	13.10	9.17		5.89	4.12		19.66	13.76		0.70	0.49	
H31	UIL.21.14566	Atyab Twin Cake Vanilla	100	6	Food	1736.42	413.99	18.43	18.43		7.17	7.17		27.82	27.82		0.56	0.56	
H7	UIL.21.13451	Britannia Chocolate Flavoured Premium Crème Wafer	32	6	Food	2152.25	513.88	25.38	8.12		15.75	5.04		33.81	10.82		0.63	0.20	
J55	UIL.21.17406	Cheese Puff (Lusine)	70	6	Food	1602.89	383.63	22.03	15.42		10.30	7.21		8.77	6.14		1.56	1.10	
J58	UIL.21.14568	Chocolate jumbo croissant	100	6	Food	1763.93	422.01	23.69	23.69		13.05	13.05		12.08	12.08		0.51	0.51	
J67H3 2	UIL.21.17531	Croissant (Dhahabi)	100	6	Food	1832.10	437.90	23.18	23.18		10.21	10.21		11.95	11.95		1.58	1.58	
H17	UIL.21.13461	Danish Butter Cookies	100	6	Food	2138.08	510.56	25.44	25.44		18.34	18.34		26.04	26.04		0.32	0.32	
J54	UIL.21.17407	Donut (Switz)	35	6	Food	1699.29	405.00	17.57	6.15		5.57	1.95		19.74	6.91		0.61	0.21	
H13	UIL.21.13457	Lotus Biscoff	7.8	6	Food	2065.38	491.67	19.36	1.51		13.59	1.06		36.41	2.84		1.05	0.08	
H2	UIL.21.13446	Nabil cream wafers (chocolate flavour)	100	6	Food	2077.35	495.05	21.29	21.29		12.74	12.74		20.63	20.63		0.37	0.37	
H1	UIL.21.13445	Nabil glucose	100	6	Food	1940.11	460.72	14.36	14.36		7.42	7.42		14.88	14.88		0.00	0.00	
H3	UIL.21.13447	Nabil Nice (sugar sprinkled coconut biscuits)	100	6	Food	1949.91	463.32	15.36	15.36		7.12	7.12		21.16	21.16		0.95	0.95	
H8	UIL.21.13452	Nutro Chocolate Flavoured Cream Wafers	30	6	Food	2239.97	535.93	30.20	9.06		26.80	8.04		36.17	10.85		0.52	0.16	
H15	UIL.21.13459	Original Oreo	19	6	Food	2027.74	482.84	19.47	3.70		8.79	1.67		42.11	8.00		1.48	0.28	
H30	UIL.21.14567	Switz Mini Cup Cake Vanilla	32	6	Food	2035.25	487.53	29.41	9.41		12.75	4.08		29.00	9.28		1.03	0.33	
H9	UIL.21.13453	Tiffany Crunch And Cream	100	6	Food	2147.08	512.36	24.36	24.36		21.51	21.51		36.23	36.23		0.31	0.31	
H5	UIL.21.13449	Tiffany Everyday Nice (Sugar Sprinkled Coconut Biscuits)	31	6	Food	2073.55	493.87	20.32	6.30		9.16	2.84		25.48	7.90		0.90	0.28	
H4	UIL.21.13448	Tiffany Glucose (Milk and Honey Biscuits)	31	6	Food	2014.55	479.26	17.84	5.53		9.16	2.84		22.90	7.10		0.91	0.28	
FA48	UIL.21.17403	Activia Full Fat Fresh Laban	100	7	Drink	254.81	60.92	3.28	3.28		2.26	2.26		4.46	4.46		0.13	0.13	
FA41	UIL.21.17409	Activia Full Fat Plain Yoghurt	100	7	Food	187.69	44.77	2.08	2.08		1.53	1.53		3.32	3.32		0.09	0.09	
FA32	UIL.21.13544	Almarai Fresh Laban Full Fat	180	7	Drink	260.74	62.42	3.64	6.56		2.57	4.63		4.09	7.36		0.14	0.25	
FA34	UIL.21.13545	Almarai Laban Up	200	7	Drink	160.94	38.21	1.15	2.29		0.81	1.62		3.99	7.97		0.80	1.61	
FA33	UIL.21.13548	Almarai Strawberry Laban	340	7	Drink	371.94	87.91	1.36	4.63		0.91	3.09		15.29	52.00		0.14	0.48	
F40	UIL.21.13546	Alrawabi Up: Laban Drink	100	7	Drink	157.69	37.63	1.79	1.79		1.20	1.20		3.04	3.04		0.00	0.00	
FA42	UIL.21.17404	Balade Farms Ayran Original Laban	100	7	Drink	132.84	31.63	1.27	1.27		1.02	1.02		0.00	0.00		0.95	0.95	
J64	UIL.21.14131	Cream (Luna)	100	7	Food	905.27	219.39	21.71	21.71		16.71	16.71		3.10	3.10		0.00	0.00	

Sr.No	Sample ID (from lab)	Name of Product & Brand	Portion (g/ml)	Nutrient Profile Group	Food Item	Energy (kJ per 100 g)	Energy (kcal per 100 g)	Fat (g per 100g)	Fat (g per serving)	FINAL FAT	SFA[1] (g per 100g)	SFA[1] (g per serving)	FINAL SFA[1]	Sugars (g per 100g)	Sugars (g per serving)	FINAL Sugars	Salt (g per 100g)	Salt (g per serving)	FINAL Salt
J21	UIL.21.14553	Cream (Puck)	100	7	Food	1000.88	242.71	24.51	24.51		11.62	11.62		0.00	0.00		0.01	0.01	
J50	UIL.21.13539	Fresh Laban (Alrawabi)	100	7	Drink	255.29	61.03	3.27	3.27		2.29	2.29		3.97	3.97		0.00	0.00	
F35	UIL.21.13594	Fresh Labneh, Full Fat Al-Marai	100	7	Food	644.71	154.97	11.13	11.13		7.82	7.82		6.63	6.63		0.54	0.54	
F33	UIL.21.13592	Fresh Labneh: Full Fat Mazoon	55	7	Food	726.60	174.75	12.85	7.07		8.82	4.85		7.33	4.03		0.57	0.31	
F36	UIL.21.13558	Fresh Yougurt Full Fat: Al-Marai	170	7	Drink	303.34	72.44	3.61	6.14		2.57	4.37		5.62	9.55		0.18	0.31	
F37	UIL.21.13556	Fresh Yougurt Full Fat: Al-Safwah	100	7	Drink	278.97	66.59	3.23	3.23		2.01	2.01		4.78	4.78		0.18	0.18	
J26	UIL.21.13549	Laban Drink (Unikai)	100	7	Drink	137.54	32.58	0.74	0.74		0.48	0.48		3.02	3.02		4.76	4.76	
J75	UIL.21.14179	Laban Drinks (Alkhamayil)	100	7	Drink	126.18	29.86	0.58	0.58		0.37	0.37		0.89	0.89		0.00	0.00	
J28	UIL.21.13543	Laban Fresh Full Cream (Asafwah)	100	7	Drink	265.44	63.43	3.31	3.31		2.37	2.37		4.07	4.07		0.17	0.17	
J27	UIL.21.13541	Laban Up (Alsafwa)	100	7	Drink	168.87	40.19	1.55	1.55		1.04	1.04		3.89	3.89		0.87	0.87	
F34	UIL.21.13593	Labneh Turkish Recipe: Puck	100	7	Food	786.38	189.21	14.21	14.21		9.82	9.82		6.43	6.43		0.79	0.79	
FA35	UIL.21.13542	Mazoon Fresh Laban Full Fat	100	7	Drink	270.51	64.62	3.30	3.30		2.22	2.22		4.48	4.48		0.13	0.13	
FA38	UIL.21.13555	Mazoon Fresh Yoghurt Full Fat	100	7	Food	285.20	68.05	3.21	3.21		2.29	2.29		5.04	5.04		0.15	0.15	
FA37	UIL.21.13540	Mazoon Laban Up	100	7	Drink	128.47	30.49	0.89	0.89		0.60	0.60		3.70	3.70		0.88	0.88	
FA40	UIL.21.14180	Mazoon Sambharam Spicy Laban Drink	100	7	Drink	121.04	28.73	0.85	0.85		0.54	0.54		1.68	1.68		0.90	0.90	
FA36	UIL.21.13547	Mazoon Strawberry Laban	100	7	Drink	429.18	102.01	3.49	3.49		2.30	2.30		14.27	14.27		0.14	0.14	
A23	UIL.21.13830	Nestle cream	100	7	Food	1003.11	242.92	23.44	23.44		13.40	13.40		0.00	0.00		0.00	0.00	
F39	UIL.21.13557	Yoghurt: Full Cream Unikai	100	7	Food	259.98	62.11	3.19	3.19		2.41	2.41		4.41	4.41		0.21	0.21	
F38	UIL.21.13559	Youghurt;Full Cream AlRawabi	100	7	Drink	288.22	68.79	3.31	3.31		2.37	2.37		4.85	4.85		0.22	0.22	
FA31	UIL.21.13840	Almarai Burger Slice Cheese	20	9	Food	1292.25	311.50	25.30	5.06		15.15	3.03		0.00	0.00		3.50	0.70	
FA27	UIL.21.13845	Almarai Cream Cheese	30	9	Food	1422.63	344.77	34.10	10.23		25.13	7.54		0.00	0.00		1.89	0.57	
FA28	UIL.21.13844	Almarai Cream Cheese Cheddar	30	9	Food	1393.10	336.87	30.87	9.26		15.50	4.65		0.00	0.00		2.93	0.88	
J23	UIL.21.14572	Chedder cheese (Almarai)	30	9	Food	1228.07	296.30	24.97	7.49		16.77	5.03		0.00	0.00		4.82	1.45	
J22	UIL.21.14571	Chedder cheese (Kraft)	100	9	Food	1272.12	306.84	25.56	25.56		17.84	17.84		0.00	0.00		4.57	4.57	
A19	UIL.21.13837	Cheese Triangles Al Maraai	30	9	Food	1245.83	301.17	27.30	8.19		17.87	5.36		0.00	0.00		2.42	0.73	
A18	UIL.21.13838	Cheese Triangles Puck	100	9	Food	1187.34	286.88	25.52	25.52		12.95	12.95		0.00	0.00		2.79	2.79	
F32	UIL.21.13835	Haloumi Cheese Al-Marai	100	9	Food	1437.78	345.91	25.87	25.87		14.12	14.12		0.00	0.00		4.82	4.82	
F31	UIL.21.13834	Haloumi Pinar	30	9	Food	1468.50	353.50	27.10	8.13		24.20	7.26		0.00	0.00		3.19	0.96	
FA22	UIL.21.13848	Kraft Cheddar Cheese Spread Original	100	9	Food	1257.12	304.09	28.21	28.21		19.12	19.12		0.00	0.00		3.09	3.09	
FA29	UIL.21.13839	Kraft Slice Cheese Original	100	9	Food	1181.41	284.32	21.56	21.56		13.17	13.17		0.00	0.00		3.44	3.44	
FA23	UIL.21.13843	Mazoon Spread Cheese	100	9	Food	1405.36	340.52	33.48	33.48		24.12	24.12		0.00	0.00		1.01	1.01	
FA24	UIL.21.13842	Mazoon Spread Cheese Cheddar	100	9	Food	1401.40	339.60	33.52	33.52		23.82	23.82		0.00	0.00		1.00	1.00	
H37	UIL.21.14554	Pinar Processed Cream Cheese Spread	100	9	Food	1288.33	312.26	31.02	31.02		27.80	27.80		0.00	0.00		1.80	1.80	
FA25	UIL.21.13847	Puck Cream Cheese	100	9	Food	1367.83	331.21	31.85	31.85		20.97	20.97		0.00	0.00		2.12	2.12	
FA26	UIL.21.13846	Puck Cream Cheese Cheddar	100	9	Food	1373.75	332.65	32.01	32.01		19.20	19.20		0.00	0.00		2.02	2.02	
FA30	UIL.21.13841	Puck Slice Cheese Original	100	9	Food	1371.75	330.90	27.66	27.66		14.04	14.04		0.00	0.00		1.55	1.55	
A17	UIL.21.13836	Triangle Cheese Abu Alwald	100	9	Food	1055.35	254.50	21.02	21.02		11.92	11.92		0.00	0.00		1.89	1.89	

Sr.No	Sample ID (from lab)	Name of Product & Brand	Portion (g/ml)	Nutrient Profile Group	Food Item	Energy (kJ per 100g)	Energy (kcal per 100g)	Fat (g per 100g)	Fat (g per serving)	FINAL FAT	SFA[1] (g per 100g)	SFA[1] (g per serving)	FINAL SFA[1]	Sugars (g per 100g)	Sugars (g per serving)	FINAL Sugars	Salt (g per 100g)	Salt (g per serving)	FINAL Salt
H12	UIL.21.13456	Britannia Toastea Wheat Rusk	31	11	Food	1841.10	436.06	9.74	3.02		4.35	1.35		26.71	8.28		0.57	0.18	
H11	UIL.21.13455	Tiffany Whole Wheat Rusks	100	11	Food	1794.15	424.65	8.49	8.49		3.80	3.80		23.11	23.11		0.57	0.57	
FA6	UIL.21.13429	Indomie Fried Noodles	100	12	Food	1974.24	470.68	20.92	20.92		11.35	11.35		8.24	8.24		3.47	3.47	
F25	UIL.21.14202	Al-Islami Ground Beef	100	14	Food	581.61	139.57	9.25	9.25		4.16	4.16		1.38	1.38		1.07	1.07	
F24	UIL.21.14203	Al-Kabeer Jumbo Hot Dogs	100	14	Food	647.82	154.69	7.69	7.69		4.31	4.31		0.00	0.00		1.37	1.37	
F30	UIL.21.14194	Al-Safa Breaded Chicken Finger	100	14	Food	961.63	228.71	8.31	8.31		2.56	2.56		0.00	0.00		1.23	1.23	
F29	UIL.21.14196	Al-Safa Chicken Burger	100	14	Food	738.77	176.89	10.41	10.41		3.01	3.01		0.00	0.00		1.00	1.00	
F26	UIL.21.14205	Al-Safa Chicken Franks	34	14	Food	859.15	206.53	14.88	5.06		8.06	2.74		0.00	0.00		3.30	1.12	
F28	UIL.21.14204	Americana Beef Hot dog	100	14	Food	756.44	181.68	12.56	12.56		5.27	5.27		0.00	0.00		1.53	1.53	
J48	UIL.21.14200	Beef Burger (Taybat)	100	14	Food	933.91	223.72	13.52	13.52		6.99	6.99		0.00	0.00		1.58	1.58	
F23	UIL.21.14199	Beef Burger Sadia	100	14	Food	1116.56	268.77	20.57	20.57		10.32	10.32		0.00	0.00		1.16	1.16	
SI1	UIL.21.14201	Corned Beef (Taybat)	50	14	Food	741.30	176.90	8.42	4.21		4.14	2.07		0.54	0.27		0.54	0.27	
H29	UIL.21.14573	Family Corned Beef	100	14	Food	901.32	215.19	10.59	10.59		6.54	6.54		4.02	4.02		1.08	1.08	
F22	UIL.21.14198	Sadia Chicken Burger	100	14	Food	920.71	221.12	15.24	15.24		5.93	5.93		0.00	0.00		1.51	1.51	
F27	UIL.21.14206	Sadia Chicken Franks	100	14	Food	844.93	203.41	15.65	15.65		6.77	6.77		0.00	0.00		1.17	1.17	
F21	UIL.21.14195	Sadia Chicken Nuggets Crispy	100	14	Food	939.87	224.19	10.35	10.35		3.21	3.21		0.00	0.00		1.06	1.06	
I20	UIL.21.13295	Light Chunks Tuna California Garden In Water	100	15	Food	458.47	108.19	1.07	1.07		0.80	0.80		0.84	0.84		0.55	0.55	
I16	UIL.21.13291	Skipjack Tuna Alalali In Sun Flower Oil	100	15	Food	868.10	207.50	11.02	11.02		1.46	1.46		0.80	0.80		1.22	1.22	
I19	UIL.21.13294	Skipjack Tuna California Garden In Sun Flower Oil	100	15	Food	570.11	135.02	2.98	2.98		0.36	0.36		0.89	0.89		0.77	0.77	
I17	UIL.21.13292	Tuna Alalali In Water	100	15	Food	450.53	106.01	0.01	0.01		0.00	0.00		0.89	0.89		1.22	1.22	
FA11	UIL.21.13434	White Tuna Meat In Brine	100	15	Food	490.75	115.65	0.61	0.61		0.46	0.46		0.10	0.10		0.97	0.97	
I18	UIL.21.13293	White Tuna Meat In Sun Flower Oil Delmonte	100	15	Food	677.53	161.06	5.58	5.58		0.80	0.80		0.36	0.36		1.08	1.08	
I14	UIL.21.13289	Baked Beans Almazraa	100	17	Food	311.63	73.71	1.31	1.31		0.26	0.26		0.74	0.74		0.39	0.39	
I7	UIL.21.13282	Baked Beans California Garden	130	17	Food	539.62	127.15	0.63	0.82		0.16	0.21		6.12	7.96		1.00	1.30	
I3	UIL.21.13278	Boildchick Peas Mara	130	17	Food	343.86	81.26	1.20	1.56		0.38	0.49		10.40	13.52		0.79	1.03	
I11	UIL.21.13286	Chick Peas Almazraa	130	17	Food	399.49	94.18	0.61	0.79		0.11	0.14		0.81	1.05		0.75	0.97	
I6	UIL.21.13281	Chick Peas California Garden	130	17	Food	484.32	114.15	0.64	0.83		0.13	0.17		0.84	1.09		0.68	0.88	
I9	UIL.21.13284	Chick Peas Luna	100	17	Food	418.75	99.00	1.60	1.60		0.74	0.74		0.76	0.76		0.88	0.88	
A16	UIL.21.14397	Diamond Strawberry Jam	100	17	Food	1148.09	270.23	0.31	0.31		0.27	0.27		63.84	63.84		0.04	0.04	
I8	UIL.21.13283	Foul Medamas Almazraa	130	17	Food	345.12	81.21	0.01	0.01		0.00	0.00		0.77	1.00		1.10	1.43	
J76	UIL.21.14557	Foul Medammas (American Garden)	130	17	Food	397.43	93.52	0.01	0.01		0.00	0.00		1.22	1.59		0.87	1.13	
I1	UIL.21.13276	Green Giant Sweet Corn	80	17	Food	352.10	83.33	1.63	1.30		0.38	0.30		7.48	5.98		0.52	0.41	
I10	UIL.21.13285	Green Peas Luna	100	17	Food	367.10	86.50	0.42	0.42		0.12	0.12		0.61	0.61		0.60	0.60	
H36	UIL.21.14555	La Ming Beans In Tomato Sauce	100	17	Food	1667.92	392.74	0.98	0.98		0.23	0.23		2.40	2.40		0.00	0.00	
H26	UIL.21.14556	Luna Baked Beans In Tomato Sauce	100	17	Food	376.77	88.74	0.30	0.30		0.19	0.19		3.96	3.96		0.81	0.81	
H27	UIL.21.14558	Lunafoul Medames Extra Grade Fava Beans	100	17	Food	422.83	99.61	0.41	0.41		0.21	0.21		1.17	1.17		0.84	0.84	
J9	UIL.21.14406	Mango Pickle / Aeroplane	100	17	Food	339.32	81.04	4.08	4.08		1.23	1.23		0.10	0.10		12.80	12.80	

Sr.No	Sample ID (from lab)	Name of Product & Brand	Portion (g/ml)	Nutrient Profile Group	Food Item	Energy (kJ per 100g)	Energy (kcal per 100g)	Fat (g per 100g)	Fat (g per serving)	FINAL FAT	SFA[1] (g per 100g)	SFA[1] (g per serving)	FINAL SFA[1]	Sugars (g per 100g)	Sugars (g per serving)	FINAL Sugars	Salt (g per 100g)	Salt (g per serving)	FINAL Salt
J6	UIL.21.14403	Mango Pickles In Oil / Ahmed	20	17	Food	980.40	236.80	20.80	4.16		2.30	0.46		0.50	0.10		7.66	1.53	
J8	UIL.21.14405	Mango Thokku Pickle In Oil / Priya	100	17	Food	701.64	166.33	4.21	4.21		1.03	1.03		22.40	22.40		11.36	11.36	
J77	UIL.21.14559	Peas processed	130	17	Food	360.42	84.81	0.01	0.01		0.00	0.00		2.46	3.20		0.55	0.71	
I2	UIL.21.13277	Plain Medammes California Garden	130	17	Food	400.31	94.19	0.01	0.01		0.00	0.00		0.93	1.21		0.87	1.13	
I12	UIL.21.13287	Processed Peas Almazraa	130	17	Food	338.58	79.67	0.01	0.01		0.00	0.00		1.64	2.13		0.83	1.07	
I4	UIL.21.13279	Processed Peas California Garden	130	17	Food	356.63	83.92	0.01	0.01		0.15	0.20		1.64	2.13		0.84	1.09	
I15	UIL.21.13290	Sweet Corn American Garden	125	17	Food	383.17	90.54	1.29	1.61		0.29	0.36		6.41	8.01		0.45	0.57	
I5	UIL.21.13280	Sweet Corn California Garden	130	17	Food	416.07	98.22	1.08	1.40		0.28	0.36		3.08	4.01		0.34	0.44	
I13	UIL.21.13288	Sweet Corn Libbys	125	17	Food	212.05	50.06	0.55	0.69		0.16	0.20		2.09	2.61		0.57	0.71	
J7	UIL.21.14404	Tomato Paste / Alalali	100	17	Food	345.39	81.48	0.72	0.72		0.38	0.38		12.30	12.30		1.04	1.04	
J 2	UIL.21.14399	Tomato Paste / Almudhish	100	17	Food	411.57	97.14	1.02	1.02		0.39	0.39		10.40	10.40		0.00	0.00	
J 3	UIL.21.14400	Tomato Paste / Jumbo	100	17	Food	361.50	85.25	0.65	0.65		0.25	0.25		4.14	4.14		0.77	0.77	
A13	UIL.21.14394	American Garden BBQ Sauce Original	36	18	Food	709.36	166.92	0.03	0.01		0.01	0.00		34.14	12.29		2.51	0.90	
18 J	UIL.21.14415	Classic Mayonnaise / Mazola	14	18	Food	2992.86	727.50	79.21	11.09		13.64	1.91		3.71	0.52		1.27	0.18	
19 j	UIL.21.14416	Creamy Classic Mayonnaise / Heinz	10	18	Food	1534.02	371.49	35.85	3.59		7.32	0.73		3.46	0.35		2.05	0.21	
A12	UIL.21.14393	Delicio 1000 Island	14	18	Food	1544.86	374.36	36.93	5.17		6.64	0.93		14.29	2.00		2.64	0.37	
A11	UIL.21.14392	Delicio French Dressing	14	18	Food	1645.43	398.00	36.86	5.16		6.64	0.93		19.79	2.77		2.84	0.40	
FA1	UIL.21.13296	Hayat Hot Sauce	100	18	Food	49.70	11.90	0.70	0.70		0.00	0.00		0.72	0.72		6.64	6.64	
A14	UIL.21.14395	Heinz Classic BBQ Smokey And Rich	100	18	Food	601.49	141.63	0.35	0.35		0.15	0.15		28.68	28.68		1.26	1.26	
J 5	UIL.21.14402	Hot Sauce / Excellence	100	18	Food	186.52	43.89	0.01	0.01		0.00	0.00		9.79	9.79		5.82	5.82	
20 J	UIL.21.14417	Hot Sause / American Garden	5	18	Food	51.60	12.20	0.20	0.01		0.04	0.00		2.00	0.10		7.09	0.35	
FA2	UIL.21.13297	Jumbo Hot Sauce	100	18	Food	24.10	5.70	0.10	0.10		0.00	0.00		0.58	0.58		4.09	4.09	
A 33	UIL.21.14563	Knour Beef Flavoured Stock	250	18	Food	12.41	2.92	0.00	0.00		0.00	0.00		0.00	0.00		1.12	2.81	
H34	UIL.21.14562	Knour CHICKEN Stock	250	18	Food	13.09	3.08	0.00	0.00		0.00	0.00		0.00	0.00		1.17	2.93	
H35	UIL.21.14561	Knour Vegetable Stock Cubes	250	18	Food	11.56	2.72	0.00	0.00		0.00	0.00		0.00	0.00		1.37	3.42	
H33	UIL.21.14564	Maggi Beef Flavour	100	18	Food	219.98	51.76	0.00	0.00		0.00	0.00		0.00	0.00		50.55	50.55	
FA7	UIL.21.13430	Maggi Chicken Stock	100	18	Food	1144.32	275.69	21.89	21.89		12.87	12.87		8.23	8.23		46.28	46.28	
A32	UIL.21.14565	Maggi Chicken Stock Less Salt	100	18	Food	494.70	116.40	0.00	0.00		0.00	0.00		0.00	0.00		37.54	37.54	
J10	UIL.21.14407	Pizza Sauce Olive& Mushrooms / Alalali	100	18	Food	257.36	61.02	1.58	1.58		0.36	0.36		6.38	6.38		0.91	0.91	
J 1	UIL.21.14398	Soy Sauce Classic / American Garden	5	18	Food	51.60	12.20	0.20	0.01		0.04	0.00		2.00	0.10		7.25	0.36	
J4	UIL.21.14401	Tabasco Hot Sauce	100	18	Food	75.68	17.81	0.01	0.01		0.00	0.00		2.65	2.65		1.75	1.75	
J11	UIL.21.14408	Tomato Ketchup / Hayat	15	18	Food	464.87	109.40	0.07	0.01		0.01	0.00		22.20	3.33		2.07	0.31	
16 J	UIL.21.14413	Tomato Ketchup / Heinz	100	18	Food	420.27	98.89	0.01	0.01		0.00	0.00		22.58	22.58		1.83	1.83	
14 J	UIL.21.14411	Tomato Ketchup/ Jumbo	100	18	Food	527.25	124.15	0.31	0.31		0.19	0.19		26.94	26.94		0.42	0.42	
A25	UIL.21.14133	Al Deyafa Fruit Cordial	100	3a	Drink	184.07	43.34	0.10	0.10		0.02	0.02		9.15	9.15		0.00	0.00	
A22	UIL.21.13828	Al Deyafa Orange Flavour (Powder)	100	3a	Drink	1093.61	257.32	0.00	0.00		0.00	0.00		60.59	60.59		0.04	0.04	
A20	UIL.21.13850	Al Marai Mango Juice	100	3a	Food	1627.58	382.96	0.00	0.00		0.00	0.00		94.69	94.69		0.00	0.00	
A26	UIL.21.14134	Al Rabie Fruit Cocktail Nectar	100	3a	Drink	271.96	64.02	0.10	0.10		0.00	0.00		11.81	11.81		0.00	0.00	
A27	UIL.21.14132	Al Rawabi Apple Juice	100	3a	Drink	263.33	61.96	0.00	0.00		0.00	0.00		12.47	12.47		0.00	0.00	
FA44	UIL.21.14138	Al Rawabi Red Grape Juice	100	3a	Drink	266.01	62.62	0.10	0.10		0.00	0.00		13.96	13.96		0.00	0.00	
A28	UIL.21.14135	Almarai Graps And Berries (Farms Select)	200	3a	Drink	245.63	57.81	0.05	0.10		0.00	0.00		13.61	27.22		0.00	0.00	

Sr.No	Sample ID (from lab)	Name of Product & Brand	Portion (g/ml)	Nutrient Profile Group	Food Item	Energy (kJ per 100g)	Energy (kcal per 100g)	Fat (g per 100g)	Fat (g per serving)	FINAL FAT	SFA[1] (g per 100g)	SFA[1] (g per serving)	FINAL SFA[1]	Sugars (g per 100g)	Sugars (g per serving)	FINAL Sugars	Salt (g per 100g)	Salt (g per serving)	FINAL Salt
H23	UIL.21.13822	Almarai Orange Juice 100% 300 ml	300	3a	Drink	203.77	47.95	0.00	0.00		0.00	0.00		9.39	28.16		0.00	0.00	
H21	UIL.21.13823	Alrawabi Orange 1005juice	100	3a	Drink	187.00	44.00	0.00	0.00		0.00	0.00		6.65	6.65		0.15	0.15	
H25	UIL.21.13821	Asafwa Natural Orange 200 ml	100	3a	Drink	219.98	51.76	0.00	0.00		0.00	0.00		10.25	10.25		0.42	0.42	
FA45	UIL.21.14139	A'safwah Mixed Fruit Juice	100	3a	Drink	244.25	57.50	0.10	0.10		0.00	0.00		11.56	11.56		0.00	0.00	
J69	UIL.21.14143	Fruit Drink (Orange) , Rani	100	3a	Drink	229.63	54.06	0.10	0.10		0.00	0.00		11.56	11.56		0.02	0.02	
J71	UIL.21.14142	Fruit Nectar (Lacnor)	100	3a	Drink	229.63	54.06	0.10	0.10		0.00	0.00		12.73	12.73		0.00	0.00	
H19	UIL.21.13829	Mango Juice Mazoon 200ml	100	3a	Drink	279.48	65.76	0.00	0.00		0.00	0.00		13.44	13.44		0.00	0.00	
J49	UIL.21.13824	Mixed Fruit Juice (Mazoon)	100	3a	Drink	225.08	52.96	0.00	0.00		0.00	0.00		11.77	11.77		0.00	0.00	
A30	UIL.21.14136	Nada Mango Juice	200	3a	Drink	221.87	52.21	0.01	0.01		0.00	0.00		10.50	21.00		0.00	0.00	
A29	UIL.21.14137	Nada Orange Juice	200	3a	Drink	188.34	44.33	0.05	0.10		0.00	0.00		10.46	20.92		0.00	0.00	
H20	UIL.21.13827	Natural Mango Asafwa 200ml	100	3a	Drink	233.58	54.96	0.00	0.00		0.00	0.00		13.13	13.13		0.01	0.01	
J70	UIL.21.14141	Orange Drink (Top Fruit)	100	3a	Drink	207.87	48.94	0.10	0.10		0.00	0.00		10.72	10.72		0.01	0.01	
SI2	UIL.21.13826	Orange No Add Sugar (Top Fruit)	100	3a	Drink	177.65	41.80	0.00	0.00		0.00	0.00		9.14	9.14		0.02	0.02	
H28	UIL.21.14140	Sun Top Orange Fruit Drink	100	3a	Drink	225.04	52.98	0.10	0.10		0.00	0.00		11.84	11.84		0.01	0.01	
J24	UIL.21.17402	Tang (Orange) (Powder)	25	3a	Food	1648.32	387.84	0.00	0.00		0.00	0.00		96.92	24.23		0.46	0.11	
H22	UIL.21.13825	Top fruit Fruit Cocktail 200ml	100	3a	Drink	193.46	45.52	0.00	0.00		0.00	0.00		9.28	9.28		0.06	0.06	
A21	UIL.21.13851	Vimto	100	3a	Drink	213.86	50.32	0.00	0.00		0.00	0.00		12.39	12.39		0.00	0.00	
J62	UIL.21.14129	Evaporated Milk (Rainbow)	100	3c	Drink	608.12	145.69	8.85	8.85		6.25	6.25		9.54	9.54		0.27	0.27	
H6	UIL.21.13450	Galaxy Smooth Milk	36	3c	Drink	1648.85	388.08	0.38	0.14		0.08	0.03		73.08	26.31		0.00	0.00	
FA39	UIL.21.13551	Mazoon Fresh Milk Strawberry Flavour	100	3c	Drink	353.71	84.17	3.21	3.21		2.36	2.36		6.47	6.47		0.14	0.14	
J60	UIL.21.14570	Milk Powder (Almudhish)	100	3c	Food	2201.83	526.76	29.52	29.52		19.24	19.24		38.95	38.95		0.71	0.71	
J61	UIL.21.14569	Milk Powder (Majan)	100	3c	Food	2187.30	523.25	29.21	29.21		20.19	20.19		47.83	47.83		0.84	0.84	
J31	UIL.21.13553	Strawberry flavored milk	250	3c	Drink	350.50	83.21	2.50	6.26		1.74	4.35		12.03	30.07		0.15	0.37	
J73	UIL.21.14171	Strawberry Flavored Milk (Lacnor)	100	3c	Drink	354.88	84.46	3.26	3.26		1.94	1.94		9.84	9.84		0.18	0.18	
J72	UIL.21.14170	Strawberry Fresh Milk (Alrawabi)	100	3c	Drink	354.39	84.33	3.21	3.21		2.27	2.27		9.67	9.67		0.12	0.12	
J29	UIL.21.13552	Strawberry Milk (Nada)	100	3c	Drink	388.23	92.06	2.42	2.42		1.78	1.78		14.14	14.14		0.15	0.15	
J30	UIL.21.13554	Strwaberry Milk (Asafwah)	100	3c	Drink	385.01	91.56	3.28	3.28		2.32	2.32		11.77	11.77		0.38	0.38	
J63	UIL.21.14130	Tea Milk (Almudhish)	100	3c	Drink	571.59	136.88	8.12	8.12		4.05	4.05		9.28	9.28		0.27	0.27	
J34	UIL.21.14173	Energy drink (Red bull)	100	3d	Drink	197.40	46.45	0.01	0.01		0.00	0.00		10.15	10.15		0.11	0.11	
A24	UIL.21.13849	Power Horse Energy	250	3d	Drink	201.55	47.42	0.00	0.00		0.00	0.00		11.28	28.20		0.17	0.42	
J38	UIL.21.14174	Coca cola	100	3e	Drink	186.69	43.93	0.01	0.01		0.00	0.00		9.78	9.78		0.04	0.04	
A31	UIL.21.14172	Lipton Peach Ice tea	100	3e	Drink	78.74	18.53	0.01	0.01		0.00	0.00		4.40	4.40		0.03	0.03	
J32	UIL.21.14177	Malt beverage (Barbican)	100	3e	Drink	55.28	13.01	0.01	0.01		0.00	0.00		2.22	2.22		0.03	0.03	
J35	UIL.21.14176	Mountain Dew	100	3e	Drink	209.81	49.37	0.01	0.01		0.00	0.00		11.55	11.55		0.04	0.04	
J36	UIL.21.14175	Pepsi	100	3e	Drink	173.94	40.93	0.01	0.01		0.00	0.00		9.98	9.98		0.03	0.03	
J33	UIL.21.14178	Vitaene Extra (Pokka)	120	3e	Drink	251.63	59.21	0.01	0.01		0.00	0.00		13.36	16.03		0.00	0.00	

1: SFA = Saturated Fatty Acids. Red colour: means the food contains high amounts of this nutrient. Amber colour: means the food is neither high nor low in this nutrient. Green colour: means the food is low in this nutrient.

Table S6: Percentage reference intake (% RI) and Scores of IR information.

Sr.No	Sample ID (from lab)	Name of Product & Brand	Percentage reference intake (% RI) information								Score of Percentage reference intake (% RI) Information							
			%RI[1] Energy (kJ)	%RI Energy (kcal)	%RI Fat	%RI SFA[2]	%RI Carbohy-drates	%RI Sugars	%RI Protein	%RI Salt	Score %RI[3] Energy (kJ)	Score %RI Energy (kcal)	Score %RI Fat	Score %RI Saturates	Score %RI Carbohydrates	Score %RI Sugars	Score %RI Protein	Score %RI Salt
17 J	UIL.21.14414	Natural Honey / Alshifa	16.93	16.73	0.00	0.00	32.17	89.22	0.00	0.00	Good	Good	Low	Low	High	High	Low	Low
15 J	UIL.21.14412	Pure honey / Capilano	16.58	16.39	0.00	0.00	31.52	90.00	0.00	0.00	Good	Good	Low	Low	High	High	Low	Low
FA19	UIL.21.13442	Batook Specialmint Chewing Gum	18.71	18.50	0.57	0.40	34.77	2.22	2.40	0.00	Good	Good	Low	Low	High	Low	Low	Low
FA16	UIL.21.13439	Bounty Minis	21.95	21.90	20.49	41.90	26.04	16.69	19.08	13.60	High	High	High	High	High	Good	Good	Good
SI3	UIL.21.13560	Cream Caramel Almaraei	6.24	6.20	3.19	8.30	8.75	23.24	6.46	2.55	Medium	Medium	Low	Medium	Medium	High	Medium	Low
13 J	UIL.21.14410	Custard Powder / Alalali	18.37	18.16	0.01	0.01	34.90	0.11	0.02	3.44	Good	Good	Low	Low	High	Low	Low	Low
J12	UIL.21.14409	Custard Powder/Tiara	17.97	17.76	0.33	0.70	33.83	0.23	0.64	0.01	Good	Good	Low	Low	High	Low	Low	Low
A15	UIL.21.14396	Daily Fresh/ Custard Powder	17.19	17.00	0.91	1.05	32.01	3.41	0.66	29.63	Good	Good	Low	Low	High	Low	Low	High
FA49	UIL.21.13550	Danet Cream Caramel	5.79	5.75	3.16	7.95	7.84	19.78	6.82	2.65	Medium	Medium	Low	Medium	Medium	Good	Medium	Low
FA18	UIL.21.13441	Extra Peppermint	25.80	25.50	0.75	0.53	48.18	58.48	2.11	0.00	High	High	Low	Low	High	High	Low	Low
FA12	UIL.21.13435	Kinder Joy	27.13	27.29	46.03	75.75	21.24	55.13	17.44	8.11	High	High	High	High	High	High	Good	Medium
FA14	UIL.21.13437	Kitkat	25.68	25.75	36.13	68.80	24.55	50.41	16.06	3.09	High	High	High	High	High	High	Good	Low
FA17	UIL.21.13440	Mars Minis	23.68	23.67	25.60	57.69	28.34	64.19	8.62	9.41	High	High	High	High	High	High	Medium	Medium
FA21	UIL.21.13444	Mentos Sugar free Chewing Gum	19.33	19.11	0.82	0.57	35.82	35.56	2.29	0.00	Good	Good	Low	Low	High	High	Low	Low
A10	UIL.21.14391	Nutella Hazelnut Spread With Cocoa	27.18	27.32	44.57	52.65	23.45	64.38	10.80	1.86	High	High	High	High	High	High	Medium	Low
FA15	UIL.21.13438	Snickers Minis	25.36	25.46	38.29	33.67	22.21	58.37	18.53	11.15	High	High	High	High	High	High	Good	Good
H10	UIL.21.13454	Twix Minis	26.10	26.17	36.21	57.25	26.52	54.00	9.70	7.87	High	High	High	High	High	High	Medium	Medium
FA20	UIL.21.13443	Wrigleys Doublemint Chewing Gum	18.85	18.63	0.55	0.38	35.21	90.60	1.54	0.00	Good	Good	Low	Low	High	High	Low	Low
A3	UIL.21.14384	Al Mudhish Puffed Corn Cheese Balls	28.21	28.40	51.07	82.92	21.22	4.91	12.83	26.29	High	High	High	High	High	Low	Good	High
FA10	UIL.21.13433	Alrifai (Mixed Nuts)	26.71	26.87	46.33	23.21	15.38	7.98	42.78	10.16	High	High	High	High	Good	Medium	High	Medium
FA8	UIL.21.13431	American Garden Popcorn Butter	23.48	23.56	34.89	54.55	21.21	0.34	15.39	41.70	High	High	High	High	High	Low	Good	High
FA9	UIL.21.13432	Best Salted Mixed Nuts	31.78	32.17	74.13	41.55	7.52	4.76	49.04	14.23	High	High	High	High	Medium	Low	High	Good
J25	UIL.21.17408	Cheese Balls (Pofak Oman)	29.02	29.18	49.05	38.33	23.79	8.30	13.60	65.26	High	High	High	High	High	Medium	Good	High
J53	UIL.21.14418	Cheese pastry (Dhahabi)	28.75	28.99	55.93	83.95	18.07	4.63	19.78	24.94	High	High	High	High	Good	Low	Good	High
J65	UIL.21.14551	Cheetos / Flamin Hot	18.88	18.93	26.49	23.70	15.90	4.28	23.14	12.99	Good	Good	High	High	Good	Low	High	Good
A1	UIL.21.13463	Cheez Balls (Mr. Krispy)	28.51	28.69	49.48	48.67	22.91	1.19	11.87	29.66	High	High	High	High	High	Low	Good	High
A5	UIL.21.14386	chips Oman	28.04	28.28	55.39	84.45	18.32	0.00	13.08	21.30	High	High	High	High	Good	Low	Good	High
A2	UIL.21.14383	Lay's (Salt)	28.03	28.22	50.71	80.36	20.66	0.00	15.00	25.16	High	High	High	High	High	Low	Good	High
A9	UIL.21.14390	Mani (Mixed Nuts)	30.83	31.19	70.47	39.60	7.29	9.50	52.02	13.39	High	High	High	High	Medium	Medium	High	Good
H14	UIL.21.13458	Nabil Cracker Salted Biscuits	23.76	23.76	27.51	67.50	25.35	22.27	19.16	42.27	High	High	High	High	High	High	Good	High

Sr.No	Sample ID (from lab)	Name of Product & Brand	Percentage reference intake (% RI) information								Score of Percentage reference intake (% RI) Information							
			%RI[1] Energy (kJ)	%RI Energy (kcal)	%RI Fat	%RI SFA[2]	%RI Carbohy-drates	%RI Sugars	%RI Protein	%RI Salt	Score %RI[3] Energy (kJ)	Score %RI Energy (kcal)	Score %RI Fat	Score %RI Saturates	Score %RI Carbohy drates	Score %RI Sugars	Score %RI Protein	Score %RI Salt
A8	UIL.21.14389	Popcorn Cheese / American Garden	26.22	26.41	48.87	78.33	18.66	0.00	13.15	41.86	High	High	High	High	Good	Low	Good	High
A4	UIL.21.14385	potato chips ready salted	27.90	28.10	51.58	78.75	20.05	0.00	14.21	22.68	High	High	High	High	High	Low	Good	High
H18	UIL.21.13462	Pringles Original	27.42	27.59	47.84	17.65	22.33	1.17	9.06	19.16	High	High	High	Good	High	Low	Medium	Good
A7	UIL.21.14388	Shai (Mix Nut)	30.63	30.98	69.20	34.35	7.96	7.61	50.42	6.38	High	High	High	High	Medium	Medium	High	Medium
J74	UIL.21.14560	Sohar Chips	27.20	27.36	46.23	71.55	23.39	0.00	6.36	40.25	High	High	High	High	High	Low	Medium	High
J51	UIL.21.13833	vanilla ice cream (Igloo)	9.81	9.86	15.90	33.40	7.90	20.67	7.47	2.87	Medium	Medium	Good	High	Medium	High	Medium	Low
J52	UIL.21.13832	vanilla ice cream (Mazoon)	6.78	6.81	10.94	22.30	5.28	13.74	6.18	1.93	Medium	Medium	Medium	High	Medium	Good	Medium	Low
FA5	UIL.21.13300	KELLOGG'S Coco Pops	19.34	19.15	3.54	5.75	31.01	27.20	19.08	0.01	Good	Good	Low	Medium	High	High	Good	Low
A6	UIL.21.14387	Kellogg's special (cereal)	19.42	19.23	3.15	2.95	28.05	15.47	36.51	29.45	Good	Good	Low	Low	High	Good	High	High
FA4	UIL.21.13299	NESTLE Chocapic	20.49	20.32	6.84	7.50	31.56	27.72	17.52	4.32	High	High	Medium	Medium	High	High	Good	Low
FA3	UIL.21.13298	Nestle Gold Corn Flakes	19.37	19.17	2.80	1.90	32.38	11.74	14.48	18.03	Good	Good	Low	Low	High	Good	Good	Good
A34	UIL.21.14574	7 days mini croissant	23.45	23.59	40.78	83.64	17.03	18.69	18.91	8.59	High	High	High	High	Good	Good	Good	Medium
H16	UIL.21.13460	Americana Premium Butter Cookies	24.45	24.46	28.96	45.80	27.12	32.67	12.34	4.76	High	High	High	High	High	High	Good	Low
J57	UIL.21.14552	Apple pastry	18.02	18.04	22.49	41.85	18.80	18.13	11.82	0.53	Good	Good	High	High	Good	Good	Good	Low
J56	UIL.21.17405	Apple puff (Lusine)	17.27	17.26	18.71	29.43	18.43	21.84	17.80	11.65	Good	Good	Good	High	Good	High	Good	Good
H31	UIL.21.14566	Atyab Twin Cake Vanilla	20.67	20.70	26.33	35.85	21.62	30.91	11.64	9.39	High	High	High	High	High	High	Good	Medium
H7	UIL.21.13451	Britannia Chocolate Flavoured Premium Crème Wafer	25.62	25.69	36.25	78.75	24.81	37.57	13.75	10.48	High	High	High	High	High	High	Good	Medium
J55	UIL.21.17406	Cheese Puff (Lusine)	19.08	19.18	31.47	51.50	13.91	9.75	20.34	26.08	Good	Good	High	High	Good	Medium	High	High
J58	UIL.21.14568	Chocolate jumbo croissant	21.00	21.10	33.84	65.25	17.10	13.42	15.50	8.46	High	High	High	High	Good	Good	Good	Medium
J67H32	UIL.21.17531	Croissant (Dhahabi)	21.81	21.90	33.11	51.05	19.27	13.28	14.42	26.31	High	High	High	High	Good	Good	Good	High
H17	UIL.21.13461	Danish Butter Cookies	25.45	25.53	36.34	91.70	24.95	28.93	11.04	5.32	High	High	High	High	High	High	Good	Medium
J54	UIL.21.17407	Donut (Switz)	20.23	20.25	25.10	27.86	21.19	21.94	13.26	10.19	High	High	High	High	High	High	Good	Medium
H13	UIL.21.13457	Lotus Biscoff	24.59	24.58	27.66	67.95	28.35	40.46	11.28	17.48	High	High	High	High	High	High	Good	Good
H2	UIL.21.13446	Nabil cream wafers (chocolate flavour)	24.73	24.75	30.41	63.70	27.02	22.92	11.22	6.23	High	High	High	High	High	High	Good	Medium
H1	UIL.21.13445	Nabil glucose	23.10	23.04	20.51	37.10	28.30	16.53	18.60	0.00	High	High	High	High	High	Good	Good	Low
H3	UIL.21.13447	Nabil Nice (sugar sprinkled coconut biscuits)	23.21	23.17	21.94	35.60	27.60	23.51	19.04	15.88	High	High	High	High	High	High	Good	Good
H8	UIL.21.13452	Nutro Chocolate Flavoured Cream Wafers	26.67	26.80	43.14	134.00	24.05	40.19	7.00	8.64	High	High	High	High	High	High	Medium	Medium
H15	UIL.21.13459	Original Oreo	24.14	24.14	27.82	43.95	27.53	46.78	10.63	24.64	High	High	High	High	High	High	Medium	High
H30	UIL.21.14567	Switz Mini Cup Cake Vanilla	24.23	24.38	42.01	63.75	18.69	32.22	14.25	17.11	High	High	High	High	Good	High	Good	Good
H9	UIL.21.13453	Tiffany Crunch And Cream	25.56	25.62	34.80	107.55	24.86	40.26	17.30	5.09	High	High	High	High	High	High	Good	Medium
H5	UIL.21.13449	Tiffany Everyday Nice (Sugar Sprinkled Coconut Biscuits)	24.69	24.69	29.03	45.81	27.42	28.32	12.90	15.02	High	High	High	High	High	High	Good	Good

Sr.No	Sample ID (from lab)	Name of Product & Brand	Percentage reference intake (% RI) information								Score of Percentage reference intake (% RI) Information							
			%RI[1] Energy (kJ)	%RI Energy (kcal)	%RI Fat	%RI SFA[2]	%RI Carbohy-drates	%RI Sugars	%RI Protein	%RI Salt	Score %RI[3] Energy (kJ)	Score %RI Energy (kcal)	Score %RI Fat	Score %RI Saturates	Score %RI Carbohydrates	Score %RI Sugars	Score %RI Protein	Score %RI Salt
H4	UIL.21.13448	Tiffany Glucose (Milk and Honey Biscuits)	23.98	23.96	25.48	45.81	27.79	25.45	14.84	15.09	High	High	High	High	High	High	Good	Good
FA48	UIL.21.17403	Activia Full Fat Fresh Laban	3.03	3.05	4.69	11.30	1.79	4.96	6.40	2.21	Low	Low	Low	Good	Low	Low	Medium	Low
FA41	UIL.21.17409	Activia Full Fat Plain Yoghurt	2.23	2.24	2.97	7.63	1.44	3.69	5.53	1.48	Low	Low	Low	Medium	Low	Low	Medium	Low
FA32	UIL.21.13544	Almarai Fresh Laban Full Fat	3.10	3.12	5.21	12.86	1.67	4.54	6.14	2.34	Low	Low	Medium	Good	Low	Low	Medium	Low
FA34	UIL.21.13545	Almarai Laban Up	1.92	1.91	1.64	4.05	1.64	4.43	5.41	13.38	Low	Low	Low	Low	Low	Low	Medium	Good
FA33	UIL.21.13548	Almarai Strawberry Laban	4.43	4.40	1.95	4.54	5.92	16.99	7.04	2.37	Low	Low	Low	Low	Medium	Good	Medium	Low
F40	UIL.21.13546	Alrawabi Up: Laban Drink	1.88	1.88	2.56	6.00	1.24	3.38	4.30	0.01	Low	Low	Low	Medium	Low	Low	Low	Low
FA42	UIL.21.17404	Balade Farms Ayran Original Laban	1.58	1.58	1.81	5.10	1.31	0.00	3.30	15.88	Low	Low	Low	Medium	Low	Low	Low	Good
J64	UIL.21.14131	Cream (Luna)	10.78	10.97	31.01	83.55	1.53	3.44	4.06	0.00	Medium	Medium	High	High	Low	Low	Low	Low
J21	UIL.21.14553	Cream (Puck)	11.92	12.14	35.01	58.10	1.11	0.00	5.30	0.11	Good	Good	High	High	Low	Low	Medium	Low
J50	UIL.21.13539	Fresh Laban (Alrawabi)	3.04	3.05	4.67	11.45	1.78	4.41	6.56	0.00	Low	Low	Low	Good	Low	Low	Medium	Low
F35	UIL.21.13594	Fresh Labneh, Full Fat Al-Marai	7.68	7.75	15.90	39.10	2.75	7.37	13.08	9.04	Medium	Medium	Good	High	Low	Medium	Good	Medium
F33	UIL.21.13592	Fresh Labneh: Full Fat Mazoon	8.65	8.74	18.36	44.09	3.41	8.14	11.82	9.47	Medium	Medium	Good	High	Low	Medium	Good	Medium
F36	UIL.21.13558	Fresh Yougurt Full Fat: Al-Marai	3.61	3.62	5.16	12.85	2.21	6.24	8.46	3.02	Low	Low	Medium	Good	Low	Medium	Medium	Low
F37	UIL.21.13556	Fresh Yougurt Full Fat: Al-Safwah	3.32	3.33	4.61	10.05	2.24	5.31	7.12	3.05	Low	Low	Low	Medium	Low	Medium	Medium	Low
J26	UIL.21.13549	Laban Drink (Unikai)	1.64	1.63	1.06	2.40	1.27	3.36	6.38	79.40	Low	Low	Low	Low	Low	Low	Medium	High
J75	UIL.21.14179	Laban Drinks (Alkhamayil)	1.50	1.49	0.83	1.85	1.62	0.99	3.88	0.00	Low	Low	Low	Low	Low	Low	Low	Low
J28	UIL.21.13543	Laban Fresh Full Cream (Asafwah)	3.16	3.17	4.73	11.85	1.96	4.52	6.62	2.90	Low	Low	Low	Good	Low	Low	Medium	Low
J27	UIL.21.13541	Laban Up (Alsafwa)	2.01	2.01	2.21	5.20	1.52	4.32	5.20	14.42	Low	Low	Low	Medium	Low	Low	Medium	Good
F34	UIL.21.13593	Labneh Turkish Recipe: Puck	9.36	9.46	20.30	49.10	2.66	7.14	16.82	13.19	Medium	Medium	High	High	Low	Medium	Good	Good
FA35	UIL.21.13542	Mazoon Fresh Laban Full Fat	3.22	3.23	4.71	11.10	1.78	4.98	8.18	2.18	Low	Low	Low	Good	Low	Low	Medium	Low
FA38	UIL.21.13555	Mazoon Fresh Yoghurt Full Fat	3.40	3.40	4.59	11.45	2.45	5.60	6.82	2.47	Low	Low	Low	Good	Low	Medium	Medium	Low
FA37	UIL.21.13540	Mazoon Laban Up	1.53	1.52	1.27	3.00	1.42	4.11	3.84	14.62	Low	Low	Low	Low	Low	Low	Low	Good
FA40	UIL.21.14180	Mazoon Sambharam Spicy Laban Drink	1.44	1.44	1.21	2.70	1.28	1.87	3.88	15.03	Low	Low	Low	Low	Low	Low	Low	Good
FA36	UIL.21.13547	Mazoon Strawberry Laban	5.11	5.10	4.99	11.50	5.53	15.86	6.56	2.30	Medium	Medium	Low	Good	Medium	Good	Medium	Low
A23	UIL.21.13830	Nestle cream	11.94	12.15	33.49	67.00	1.88	0.00	6.18	0.00	Good	Good	High	High	Low	Low	Medium	Low
F39	UIL.21.13557	Yoghurt: Full Cream Unikai	3.10	3.11	4.56	12.05	1.86	4.90	7.04	3.50	Low	Low	Low	Good	Low	Low	Medium	Low
F38	UIL.21.13559	Youghurt;Full Cream AlRawabi	3.43	3.44	4.73	11.85	2.09	5.39	8.62	3.65	Low	Low	Low	Good	Low	Medium	Medium	Low
FA31	UIL.21.13840	Almarai Burger Slice Cheese	15.38	15.58	36.14	75.75	2.21	0.00	30.40	58.34	Good	Good	High	High	Low	Low	High	High
FA27	UIL.21.13845	Almarai Cream Cheese	16.94	17.24	48.71	125.67	1.64	0.00	10.40	31.54	Good	Good	High	High	Low	Low	Medium	High
FA28	UIL.21.13844	Almarai Cream Cheese Cheddar	16.58	16.84	44.10	77.50	1.74	0.00	20.47	48.77	Good	Good	High	High	Low	Low	High	High
J23	UIL.21.14572	Chedder cheese (Almarai)	14.62	14.82	35.67	83.83	0.00	0.00	35.80	80.38	Good	Good	High	High	Low	Low	High	High

Sr.No	Sample ID (from lab)	Name of Product & Brand	Percentage reference intake (% RI) information								Score of Percentage reference intake (% RI) Information							
			%RI[1] Energy (kJ)	%RI Energy (kcal)	%RI Fat	%RI SFA[3]	%RI Carbohy-drates	%RI Sugars	%RI Protein	%RI Salt	Score %RI[3] Energy (kJ)	Score %RI Energy (kcal)	Score %RI Fat	Score %RI Saturates	Score %RI Carbohydrates	Score %RI Sugars	Score %RI Protein	Score %RI Salt
J22	UIL.21.14571	Chedder cheese (Kraft)	15.14	15.34	36.51	89.20	2.17	0.00	27.12	76.20	Good	Good	High	High	Low	Low	High	High
A19	UIL.21.13837	Cheese Triangles Al Maraai	14.83	15.06	39.00	89.33	1.35	0.00	20.73	40.30	Good	Good	High	High	Low	Low	High	High
A18	UIL.21.13838	Cheese Triangles Puck	14.14	14.34	36.46	64.75	2.24	0.00	16.94	46.51	Good	Good	High	High	Low	Low	Good	High
F32	UIL.21.13835	Haloumi Cheese Al-Marai	17.12	17.30	36.96	70.60	1.52	0.00	48.64	80.28	Good	Good	High	High	Low	Low	High	High
F31	UIL.21.13834	Haloumi Pinar	17.48	17.68	38.71	121.00	1.36	0.00	47.73	53.09	Good	Good	High	High	Low	Low	High	High
FA22	UIL.21.13848	Kraft Cheddar Cheese Spread Original	14.97	15.20	40.30	95.60	1.62	0.00	16.68	51.46	Good	Good	High	High	Low	Low	Good	High
FA29	UIL.21.13839	Kraft Slice Cheese Original	14.06	14.22	30.80	65.85	2.86	0.00	30.28	57.41	Good	Good	High	High	Low	Low	High	High
FA23	UIL.21.13843	Mazoon Spread Cheese	16.73	17.03	47.83	120.60	1.18	0.00	13.44	16.76	Good	Good	High	High	Low	Low	Good	Good
FA24	UIL.21.13842	Mazoon Spread Cheese Cheddar	16.68	16.98	47.89	119.10	0.91	0.00	14.24	16.73	Good	Good	High	High	Low	Low	Good	Good
H37	UIL.21.14554	Pinar Processed Cream Cheese Spread	15.34	15.61	44.31	139.00	0.62	0.00	13.32	30.03	Good	Good	High	High	Low	Low	Good	High
FA25	UIL.21.13847	Puck Cream Cheese	16.28	16.56	45.50	104.85	0.96	0.00	17.30	35.40	Good	Good	High	High	Low	Low	Good	High
FA26	UIL.21.13846	Puck Cream Cheese Cheddar	16.35	16.63	45.73	96.00	1.01	0.00	17.04	33.68	Good	Good	High	High	Low	Low	Good	High
FA30	UIL.21.13841	Puck Slice Cheese Original	16.33	16.55	39.51	70.20	2.18	0.00	29.62	25.76	Good	Good	High	High	Low	Low	High	High
A17	UIL.21.13836	Triangle Cheese Abu Alwald	12.56	12.73	30.03	59.60	3.12	0.00	16.44	31.55	Good	Good	High	High	Low	Low	Good	High
H12	UIL.21.13456	Britannia Toastea Wheat Rusk	21.92	21.80	13.92	21.77	29.80	29.67	19.23	9.45	High	High	Good	High	High	High	Good	Medium
H11	UIL.21.13455	Tiffany Whole Wheat Rusks	21.36	21.23	12.13	19.00	29.59	25.68	20.26	9.53	High	High	Good	Good	High	High	High	Medium
FA6	UIL.21.13429	Indomie Fried Noodles	23.50	23.53	29.89	56.75	23.87	9.16	17.08	57.76	High	High	High	High	High	Medium	Good	High
F25	UIL.21.14202	Al-Islami Ground Beef	6.92	6.98	13.21	20.80	0.90	1.53	23.48	17.80	Medium	Medium	Good	High	Low	Low	High	Good
F24	UIL.21.14203	Al-Kabeer Jumbo Hot Dogs	7.71	7.73	10.99	21.55	1.63	0.00	34.24	22.84	Medium	Medium	Medium	High	Low	Low	High	High
F30	UIL.21.14194	Al-Safa Breaded Chicken Finger	11.45	11.44	11.87	12.80	7.57	0.00	37.60	20.56	Good	Good	Good	Good	Medium	Low	High	High
F29	UIL.21.14196	Al-Safa Chicken Burger	8.79	8.84	14.87	15.05	1.52	0.00	33.72	16.73	Medium	Medium	Good	Good	Low	Low	High	Good
F26	UIL.21.14205	Al-Safa Chicken Franks	10.23	10.33	21.26	40.29	2.25	0.00	24.59	55.08	Medium	Medium	High	High	Low	Low	High	High
F28	UIL.21.14204	Americana Beef Hot dog	9.01	9.08	17.94	26.35	0.81	0.00	30.10	25.54	Medium	Medium	Good	High	Low	Low	High	High
J48	UIL.21.14200	Beef Burger (Taybat)	11.12	11.19	19.31	34.95	2.46	0.00	38.22	26.27	Good	Good	Good	High	Low	Low	High	High
F23	UIL.21.14199	Beef Burger Sadia	13.29	13.44	29.39	51.60	2.58	0.00	28.42	19.29	Good	Good	High	High	Low	Low	High	Good
SI1	UIL.21.14201	Corned Beef (Taybat)	8.83	8.85	12.03	20.70	4.92	0.60	25.00	8.94	Medium	Medium	Good	High	Low	Low	High	Medium
H29	UIL.21.14573	Family Corned Beef	10.73	10.76	15.13	32.70	1.96	4.47	49.74	18.02	Medium	Medium	Good	High	Low	Low	High	Good
F22	UIL.21.14198	Sadia Chicken Burger	10.96	11.06	21.77	29.65	2.61	0.00	28.42	25.19	Medium	Medium	High	High	Low	Low	High	High
F27	UIL.21.14206	Sadia Chicken Franks	10.06	10.17	22.36	33.85	1.04	0.00	25.88	19.53	Medium	Medium	High	High	Low	Low	High	Good
F21	UIL.21.14195	Sadia Chicken Nuggets Crispy	11.19	11.21	14.79	16.05	8.03	0.00	23.78	17.60	Good	Good	Good	Good	Medium	Low	High	Good
I20	UIL.21.13295	Light Chunks Tuna California Garden In Water	5.46	5.41	1.53	4.00	0.37	0.93	47.38	9.11	Medium	Medium	Low	Low	Low	Low	High	Medium

Sr.No	Sample ID (from lab)	Name of Product & Brand	Percentage reference intake (% RI) information								Score of Percentage reference intake (% RI) Information							
			%RI[1] Energy (kJ)	%RI Energy (kcal)	%RI Fat	%RI SFA[3]	%RI Carbohy-drates	%RI Sugars	%RI Protein	%RI Salt	Score %RI[3] Energy (kJ)	Score %RI Energy (kcal)	Score %RI Fat	Score %RI Saturates	Score %RI Carbohydrates	Score %RI Sugars	Score %RI Protein	Score %RI Salt
I16	UIL.21.13291	Skipjack Tuna Alalali In Sun Flower Oil	10.33	10.38	15.74	7.30	1.52	0.89	46.24	20.33	Medium	Medium	Good	Medium	Low	Low	High	High
I19	UIL.21.13294	Skipjack Tuna California Garden In Sun Flower Oil	6.79	6.75	4.26	1.80	0.77	0.99	50.12	12.84	Medium	Medium	Low	Low	Low	Low	High	Good
I17	UIL.21.13292	Tuna Alalali In Water	5.36	5.30	0.01	0.01	1.27	0.99	46.38	20.30	Medium	Medium	Low	Low	Low	Low	High	High
FA11	UIL.21.13434	White Tuna Meat In Brine	5.84	5.78	0.87	2.30	0.76	0.11	51.14	16.12	Medium	Medium	Low	Low	Low	Low	High	Good
I18	UIL.21.13293	White Tuna Meat In Sun Flower Oil Delmonte	8.07	8.05	7.97	4.00	1.14	0.40	49.50	18.00	Medium	Medium	Medium	Low	Low	Low	High	Good
I14	UIL.21.13289	Baked Beans Almazraa	3.71	3.69	1.87	1.30	4.37	0.82	8.22	6.50	Low	Low	Low	Low	Low	Low	Medium	Medium
I7	UIL.21.13282	Baked Beans California Garden	6.42	6.36	0.90	0.81	9.99	6.80	8.80	16.61	Medium	Medium	Low	Low	Medium	Medium	Medium	Good
I3	UIL.21.13278	Boildchick Peas Mara	4.09	4.06	1.71	1.88	4.78	11.56	10.40	13.25	Low	Low	Low	Low	Low	Good	Medium	Good
I11	UIL.21.13286	Chick Peas Almazraa	4.76	4.71	0.87	0.54	6.21	0.90	12.08	12.45	Low	Low	Low	Low	Medium	Low	Good	Good
I6	UIL.21.13281	Chick Peas California Garden	5.77	5.71	0.91	0.65	7.68	0.93	14.25	11.28	Medium	Medium	Low	Low	Medium	Low	Good	Good
I9	UIL.21.13284	Chick Peas Luna	4.99	4.95	2.29	3.70	6.32	0.84	9.42	14.65	Low	Low	Low	Low	Medium	Low	Medium	Good
A16	UIL.21.14397	Diamond Strawberry Jam	13.67	13.51	0.44	1.35	25.57	70.93	0.78	0.64	Good	Good	Low	Low	High	High	Low	Low
I8	UIL.21.13283	Foul Medamas Almazraa	4.11	4.06	0.01	0.01	5.81	0.85	10.35	18.37	Low	Low	Low	Low	Medium	Low	Medium	Good
J76	UIL.21.14557	Foul Medammas (American Garden)	4.73	4.68	0.01	0.01	6.36	1.36	13.66	14.43	Low	Low	Low	Low	Medium	Low	Good	Good
I1	UIL.21.13276	Green Giant Sweet Corn	4.19	4.17	2.32	1.88	5.83	8.31	4.03	8.61	Low	Low	Low	Low	Medium	Medium	Low	Medium
I10	UIL.21.13285	Green Peas Luna	4.37	4.33	0.60	0.60	6.31	0.68	8.54	10.03	Low	Low	Low	Low	Medium	Low	Medium	Medium
H36	UIL.21.14555	La Ming Beans In Tomato Sauce	19.86	19.64	1.40	1.15	33.98	2.67	15.28	0.00	Good	Good	Low	Low	High	Low	Good	Low
H26	UIL.21.14556	Luna Baked Beans In Tomato Sauce	4.49	4.44	0.43	0.95	5.55	4.40	14.14	13.55	Low	Low	Low	Low	Medium	Low	Good	Good
H27	UIL.21.14558	Lunafoul Medames Extra Grade Fava Beans	5.03	4.98	0.59	1.05	6.42	1.30	14.60	13.97	Low	Low	Low	Low	Medium	Low	Good	Good
J9	UIL.21.14406	Mango Pickle / Aeroplane	4.04	4.05	5.83	6.15	3.70	0.11	2.92	213.36	Low	Low	Medium	Medium	Low	Low	Low	High
J6	UIL.21.14403	Mango Pickles In Oil / Ahmed	11.67	11.84	29.71	11.50	3.60	0.56	6.10	127.68	Good	Good	High	Good	Low	Low	Medium	High
J8	UIL.21.14405	Mango Thokku Pickle In Oil / Priya	8.35	8.32	6.01	5.15	10.78	24.89	8.14	189.25	Medium	Medium	Medium	Medium	Medium	High	Medium	High
J77	UIL.21.14559	Peas processed	4.29	4.24	0.01	0.01	6.61	2.74	7.98	9.09	Low	Low	Low	Low	Medium	Low	Medium	Medium
I2	UIL.21.13277	Plain Medammes California Garden	4.77	4.71	0.01	0.01	6.73	1.03	12.05	14.50	Low	Low	Low	Low	Medium	Low	Good	Good
I12	UIL.21.13287	Processed Peas Almazraa	4.03	3.98	0.01	0.01	5.93	1.82	8.95	13.78	Low	Low	Low	Low	Medium	Low	Medium	Good
I4	UIL.21.13279	Processed Peas California Garden	4.25	4.20	0.01	0.77	6.30	1.82	9.18	13.94	Low	Low	Low	Low	Medium	Low	Medium	Good
I15	UIL.21.13290	Sweet Corn American Garden	4.56	4.53	1.84	1.44	6.63	7.12	5.01	7.56	Low	Low	Low	Low	Medium	Medium	Medium	Medium
I5	UIL.21.13280	Sweet Corn California Garden	4.95	4.91	1.54	1.38	7.68	3.43	4.31	5.62	Low	Low	Low	Low	Medium	Low	Low	Medium
I13	UIL.21.13288	Sweet Corn Libbys	2.52	2.50	0.79	0.80	3.69	2.32	3.36	9.43	Low	Low	Low	Low	Low	Low	Low	Medium
J7	UIL.21.14404	Tomato Paste / Alalali	4.11	4.07	1.03	1.90	5.85	13.67	7.08	17.38	Low	Low	Low	Low	Medium	Good	Medium	Good
J 2	UIL.21.14399	Tomato Paste / Almudhish	4.90	4.86	1.46	1.95	6.48	11.56	10.30	0.02	Low	Low	Low	Low	Medium	Good	Medium	Low
J 3	UIL.21.14400	Tomato Paste / Jumbo	4.30	4.26	0.93	1.25	6.83	4.60	4.18	12.75	Low	Low	Low	Low	Medium	Low	Low	Good

Sr.No	Sample ID (from lab)	Name of Product & Brand	Percentage reference intake (% RI) information								Score of Percentage reference intake (% RI) Information							
			%RI[1] Energy (kJ)	%RI Energy (kcal)	%RI Fat	%RI SFA[3]	%RI Carbohy-drates	%RI Sugars	%RI Protein	%RI Salt	Score %RI[3] Energy (kJ)	Score %RI Energy (kcal)	Score %RI Fat	Score %RI Saturates	Score %RI Carbohy drates	Score %RI Sugars	Score %RI Protein	Score %RI Salt
A13	UIL.21.14394	American Garden BBQ Sauce Original	8.44	8.35	0.04	0.03	16.01	37.93	0.06	41.90	Medium	Medium	Low	Low	Good	High	Low	High
18 J	UIL.21.14415	Classic Mayonnaise / Mazola	35.63	36.38	113.16	68.21	1.37	4.13	0.14	21.10	High	High	High	High	Low	Low	Low	High
19 j	UIL.21.14416	Creamy Classic Mayonnaise / Heinz	18.26	18.57	51.21	36.60	4.67	3.84	0.14	34.17	Good	Good	High	High	Low	Low	Low	High
A12	UIL.21.14393	Delicio 1000 Island	18.39	18.72	52.76	33.21	3.71	15.87	1.71	44.04	Good	Good	High	High	Low	Good	Low	High
A11	UIL.21.14392	Delicio French Dressing	19.59	19.90	52.65	33.21	5.96	21.98	2.14	47.30	Good	Good	High	High	Medium	High	Low	High
FA1	UIL.21.13296	Hayat Hot Sauce	0.59	0.60	1.00	0.01	0.30	0.80	1.26	110.74	Low	Low	Low	Low	Low	Low	Low	High
A14	UIL.21.14395	Heinz Classic BBQ Smokey And Rich	7.16	7.08	0.50	0.75	12.99	31.87	1.70	20.98	Medium	Medium	Low	Low	Good	High	Low	High
J 5	UIL.21.14402	Hot Sauce / Excellence	2.22	2.19	0.01	0.01	4.08	10.88	0.66	96.93	Low	Low	Low	Low	Low	Medium	Low	High
20 J	UIL.21.14417	Hot Sause / American Garden	0.61	0.61	0.29	0.20	0.77	2.22	1.20	118.11	Low	Low	Low	Low	Low	Low	Low	High
FA2	UIL.21.13297	Jumbo Hot Sauce	0.29	0.29	0.14	0.01	0.33	0.64	0.68	68.17	Low	Low	Low	Low	Low	Low	Low	High
A 33	UIL.21.14563	Knour Beef Flavoured Stock	0.15	0.15	0.00	0.00	0.17	0.00	0.58	18.75	Low	Low	Low	Low	Low	Low	Low	Good
H34	UIL.21.14562	Knour CHICKEN Stock	0.16	0.15	0.00	0.00	0.20	0.00	0.50	19.50	Low	Low	Low	Low	Low	Low	Low	Good
H35	UIL.21.14561	Knour Vegetable Stock Cubes	0.14	0.14	0.00	0.00	0.14	0.00	0.62	22.83	Low	Low	Low	Low	Low	Low	Low	High
H33	UIL.21.14564	Maggi Beef Flavour	2.62	2.59	0.00	0.00	4.98	0.00	0.00	842.43	Low	Low	Low	Low	Low	Low	Low	High
FA7	UIL.21.13430	Maggi Chicken Stock	13.62	13.78	31.27	64.35	5.58	9.14	10.32	771.31	Good	Good	High	High	Medium	Medium	Medium	High
A32	UIL.21.14565	Maggi Chicken Stock Less Salt	5.89	5.82	0.00	0.00	11.19	0.00	0.00	625.69	Medium	Medium	Low	Low	Good	Low	Low	High
J10	UIL.21.14407	Pizza Sauce Olive& Mushrooms / Alalali	3.06	3.05	2.26	1.80	3.71	7.09	4.10	15.16	Low	Low	Low	Low	Low	Medium	Low	Good
J 1	UIL.21.14398	Soy Sauce Classic / American Garden	0.61	0.61	0.29	0.20	0.77	2.22	1.20	120.78	Low	Low	Low	Low	Low	Low	Low	High
J4	UIL.21.14401	Tabasco Hot Sauce	0.90	0.89	0.01	0.01	1.09	2.94	3.18	29.22	Low	Low	Low	Low	Low	Low	Low	High
J11	UIL.21.14408	Tomato Ketchup / Hayat	5.53	5.47	0.10	0.07	10.23	24.67	1.20	34.54	Medium	Medium	Low	Low	Medium	High	Low	High
16 J	UIL.21.14413	Tomato Ketchup / Heinz	5.00	4.94	0.01	0.01	8.96	25.09	2.80	30.54	Low	Low	Low	Low	Medium	High	Low	High
14 J	UIL.21.14411	Tomato Ketchup/ Jumbo	6.28	6.21	0.44	0.95	11.29	29.93	1.98	6.92	Medium	Medium	Low	Low	Good	High	Low	Medium
A25	UIL.21.14133	Al Deyafa Fruit Cordial	2.19	2.17	0.14	0.10	3.98	10.17	0.52	0.00	Low	Low	Low	Low	Low	Medium	Low	Low
A22	UIL.21.13828	Al Deyafa Orange Flavour (Powder)	13.02	12.87	0.00	0.00	24.74	67.32	0.00	0.64	Good	Good	Low	Low	High	High	Low	Low
A20	UIL.21.13850	Al Marai Mango Juice	19.38	19.15	0.00	0.00	36.82	105.21	0.02	0.01	Good	Good	Low	Low	High	High	Low	Low
A26	UIL.21.14134	Al Rabie Fruit Cocktail Nectar	3.24	3.20	0.14	0.01	5.92	13.12	0.76	0.00	Low	Low	Low	Low	Medium	Good	Low	Low
A27	UIL.21.14132	Al Rawabi Apple Juice	3.13	3.10	0.00	0.00	5.96	13.86	0.00	0.00	Low	Low	Low	Low	Medium	Good	Low	Low
FA44	UIL.21.14138	Al Rawabi Red Grape Juice	3.17	3.13	0.14	0.01	5.84	15.51	0.50	0.00	Low	Low	Low	Low	Medium	Good	Low	Low
A28	UIL.21.14135	Almarai Graps And Berries (Farms Select)	2.92	2.89	0.07	0.01	5.42	15.12	0.52	0.00	Low	Low	Low	Low	Medium	Good	Low	Low
H23	UIL.21.13822	Almarai Orange Juice 100% 300 ml	2.43	2.40	0.00	0.00	3.69	10.43	4.80	0.00	Low	Low	Low	Low	Low	Medium	Low	Low
H21	UIL.21.13823	Alrawabi Orange 1005juice	2.23	2.20	0.00	0.00	4.23	7.39	0.00	2.54	Low	Low	Low	Low	Low	Medium	Low	Low
H25	UIL.21.13821	Asafwa Natural Orange 200 ml	2.62	2.59	0.00	0.00	4.98	11.39	0.00	6.99	Low	Low	Low	Low	Low	Good	Low	Medium
FA45	UIL.21.14139	A'safwah Mixed Fruit Juice	2.91	2.88	0.14	0.01	5.41	12.84	0.16	0.00	Low	Low	Low	Low	Medium	Good	Low	Low

Sr.No	Sample ID (from lab)	Name of Product & Brand	Percentage reference intake (% RI) information								Score of Percentage reference intake (% RI) Information							
			%RI[1] Energy (kJ)	%RI Energy (kcal)	%RI Fat	%RI SFA[2]	%RI Carbohy-drates	%RI Sugars	%RI Protein	%RI Salt	Score %RI[3] Energy (kJ)	Score %RI Energy (kcal)	Score %RI Fat	Score %RI Saturates	Score %RI Carbohydrates	Score %RI Sugars	Score %RI Protein	Score %RI Salt
J69	UIL.21.14143	Fruit Drink (Orange) , Rani	2.73	2.70	0.14	0.01	5.05	12.84	0.32	0.32	Low	Low	Low	Low	Medium	Good	Low	Low
J71	UIL.21.14142	Fruit Nectar (Lacnor)	2.73	2.70	0.14	0.01	5.08	14.14	0.18	0.07	Low	Low	Low	Low	Medium	Good	Low	Low
H19	UIL.21.13829	Mango Juice Mazoon 200ml	3.33	3.29	0.00	0.00	6.32	14.93	0.00	0.00	Low	Low	Low	Low	Medium	Good	Low	Low
J49	UIL.21.13824	Mixed Fruit Juice (Mazoon)	2.68	2.65	0.00	0.00	5.09	13.08	0.00	0.00	Low	Low	Low	Low	Medium	Good	Low	Low
A30	UIL.21.14136	Nada Mango Juice	2.64	2.61	0.01	0.01	4.92	11.67	0.52	0.00	Low	Low	Low	Low	Low	Good	Low	Low
A29	UIL.21.14137	Nada Orange Juice	2.24	2.22	0.07	0.01	3.95	11.62	1.40	0.00	Low	Low	Low	Low	Low	Good	Low	Low
H20	UIL.21.13827	Natural Mango Asafwa 200ml	2.78	2.75	0.00	0.00	5.28	14.59	0.00	0.22	Low	Low	Low	Low	Medium	Good	Low	Low
J70	UIL.21.14141	Orange Drink (Top Fruit)	2.47	2.45	0.14	0.01	4.58	11.91	0.18	0.24	Low	Low	Low	Low	Low	Good	Low	Low
SI2	UIL.21.13826	Orange No Add Sugar (Top Fruit)	2.11	2.09	0.00	0.00	4.02	10.16	0.00	0.36	Low	Low	Low	Low	Low	Medium	Low	Low
H28	UIL.21.14140	Sun Top Orange Fruit Drink	2.68	2.65	0.14	0.01	4.87	13.16	0.70	0.15	Low	Low	Low	Low	Low	Good	Low	Low
J24	UIL.21.17402	Tang (Orange) (Powder)	19.62	19.39	0.00	0.00	37.28	107.69	0.08	7.66	Good	Good	Low	Low	High	High	Low	Medium
H22	UIL.21.13825	Top fruit Fruit Cocktail 200ml	2.30	2.28	0.00	0.00	4.38	10.31	0.00	0.97	Low	Low	Low	Low	Low	Medium	Low	Low
A21	UIL.21.13851	Vimto	2.55	2.52	0.00	0.00	4.84	13.77	0.00	0.00	Low	Low	Low	Low	Low	Good	Low	Low
J62	UIL.21.14129	Evaporated Milk (Rainbow)	7.24	7.28	12.64	31.25	4.07	10.60	11.88	4.46	Medium	Medium	Good	High	Low	Medium	Good	Low
H6	UIL.21.13450	Galaxy Smooth Milk	19.63	19.40	0.55	0.38	35.50	81.20	7.69	0.00	Good	Good	Low	Low	High	High	Medium	Low
FA39	UIL.21.13551	Mazoon Fresh Milk Strawberry Flavour	4.21	4.21	4.59	11.80	4.13	7.19	6.14	2.29	Low	Low	Low	Good	Low	Medium	Medium	Low
J60	UIL.21.14570	Milk Powder (Almudhish)	26.21	26.34	42.17	96.20	15.53	43.28	49.76	11.89	High	High	High	High	Good	High	High	Good
J61	UIL.21.14569	Milk Powder (Majan)	26.04	26.16	41.73	100.95	15.77	53.14	48.20	13.96	High	High	High	High	Good	High	High	Good
J31	UIL.21.13553	Strawberry flavored milk	4.17	4.16	3.58	8.70	4.67	13.36	6.07	2.46	Low	Low	Low	Medium	Low	Good	Medium	Low
J73	UIL.21.14171	Strawberry Flavored Milk (Lacnor)	4.22	4.22	4.66	9.70	4.22	10.93	5.64	2.94	Low	Low	Low	Medium	Low	Medium	Medium	Low
J72	UIL.21.14170	Strawberry Fresh Milk (Alrawabi)	4.22	4.22	4.59	11.35	4.49	10.74	4.36	2.07	Low	Low	Low	Good	Low	Medium	Low	Low
J29	UIL.21.13552	Strawberry Milk (Nada)	4.62	4.60	3.46	8.90	5.44	15.71	6.84	2.47	Low	Low	Low	Medium	Medium	Good	Medium	Low
J30	UIL.21.13554	Strwaberry Milk (Asafwah)	4.58	4.58	4.69	11.61	4.41	13.08	8.10	6.38	Low	Low	Low	Good	Low	Good	Medium	Medium
J63	UIL.21.14130	Tea Milk (Almudhish)	6.80	6.84	11.60	20.25	4.02	10.31	10.98	4.47	Medium	Medium	Good	High	Low	Medium	Medium	Low
J34	UIL.21.14173	Energy drink (Red bull)	2.35	2.32	0.01	0.01	4.45	11.28	0.02	1.91	Low	Low	Low	Low	Low	Good	Low	Low
A24	UIL.21.13849	Power Horse Energy	2.40	2.37	0.00	0.00	4.54	12.53	0.08	2.80	Low	Low	Low	Low	Low	Good	Low	Low
J38	UIL.21.14174	Coca cola	2.22	2.20	0.01	0.01	4.21	10.87	0.02	0.65	Low	Low	Low	Low	Low	Medium	Low	Low
A31	UIL.21.14172	Lipton Peach Ice tea	0.94	0.93	0.01	0.01	1.73	4.89	0.20	0.45	Low	Low	Low	Low	Low	Low	Low	Low
J32	UIL.21.14177	Malt beverage (Barbican)	0.66	0.65	0.01	0.01	1.14	2.47	0.52	0.43	Low	Low	Low	Low	Low	Low	Low	Low

Sr.No	Sample ID (from lab)	Name of Product & Brand	Percentage reference intake (% RI) information								Score of Percentage reference intake (% RI) Information							
			%RI[1] Energy (kJ)	%RI Energy (kcal)	%RI Fat	%RI SFA[2]	%RI Carbohy-drates	%RI Sugars	%RI Protein	%RI Salt	Score %RI[3] Energy (kJ)	Score %RI Energy (kcal)	Score %RI Fat	Score %RI Saturates	Score %RI Carbohydrates	Score %RI Sugars	Score %RI Protein	Score %RI Salt
J35	UIL.21.14176	Mountain Dew	2.50	2.47	0.01	0.01	4.73	12.83	0.02	0.66	Low	Low	Low	Low	Low	Good	Low	Low
J36	UIL.21.14175	Pepsi	2.07	2.05	0.01	0.01	3.92	11.09	0.02	0.44	Low	Low	Low	Low	Low	Good	Low	Low
J33	UIL.21.14178	Vitaene Extra (Pokka)	3.00	2.96	0.01	0.01	5.68	14.84	0.02	0.00	Low	Low	Low	Low	Medium	Good	Low	Low

1: %RI = percent of reference intake. 2: SFA = Saturated Fatty Acid. 3: Score % RI = the formula used score % RI = ((Amount of per 100 g/ml)/RI) * 100

The food item is considered a "low" source of the considered nutrient if the percentage of reference intake (% RI) is < 5%; "medium" if between 5 and 10%; "good source" if % RI between 11 and 20%; "high" if % RI > 20%.

Table S7: Label and laboratory values comparison.

id_lab	Name of Product & Brand	Amounts per 100 g/ml from Laboratory analyses						Amounts per 100 g/ml from Labelling						Tolerances for the nutrition declaration on food products											
														Prot (g)		Carb (g)		TF (g)		SFA (g)		Sugars (g)		Na (g)	
		Prot[1] (g)	Carb[2] (g)	TF[3] (g)	SFA[4] (g)	Sugars (g)	Na5 (g)	Prot (g)	Carb (g)	TF (g)	SFA (g)	Sugars (g)	Na (g)	Low	High	Low	High	Low	High	Low	High	Low	High	Low	High
UIL.21.14414	Natural Honey / Alshifa	0.00	83.64	0.00	0.00	80.30	0.000	0.00	83.00	0.00	0.00	71.00	0.000	0.00	0.00	75.00	91.00	0.00	0.00	0.00	0.00	63.00	79.00	0.00	0.00
UIL.21.14412	Pure honey / Capilano	0.00	81.94	0.00	0.00	81.00	0.000	0.30	83.10	0.00	0.00	82.50	0.015	0.00	2.30	75.10	91.10	0.00	0.00	0.00	0.00	74.50	90.50	0.00	0.17
UIL.21.13442	BATOOK SPECIALMINT CHEWING GUM	1.20	90.40	0.40	0.08	2.00	0.000	0.00	2.00	0.00		2.00	0.000	0.00	0.00	0.00	4.00	0.00	0.00			0.00	4.00	0.00	0.00
UIL.21.13439	BOUNTY MINIS	9.54	67.70	14.34	8.38	15.02	0.321	1.00	17.00	7.00	6.00	14.00	0.028	0.00	3.00	13.60	20.40	5.50	8.50	4.80	7.20	11.20	16.80	0.00	0.18
UIL.21.13560	CREAM CARAMEL ALMARAEI	3.23	22.76	2.23	1.66	20.92	0.060	3.00	22.00	2.00	1.00	21.00	0.054	1.00	5.00	17.60	26.40	0.50	3.50	0.20	1.80	16.80	25.20	0.00	0.20
UIL.21.14410	Custard powder / Alalali	0.01	90.75	0.01	0.00	0.10	0.081	0.00	91.00	0.00	0.00	0.00	0.080	0.00	0.00	83.00	99.00	0.00	0.00	0.00	0.00	0.00	0.00	0.00	0.23
UIL.21.14409	Custard powder/Tiara	0.32	87.95	0.23	0.14	0.21	0.000																		
UIL.21.14396	daily fresh/ custard powder	0.33	83.23	0.64	0.21	3.07	0.700	0.10	82.20	0.50	0.10		0.698	0.00	2.10	74.20	90.20	0.00	2.00	0.00	0.90			0.56	0.84
UIL.21.13550	DANET CREAM CARAMEL	3.41	20.38	2.21	1.59	17.80	0.063	29.00	21.00	2.00			0.000	23.20	34.80	16.80	25.20	0.50	3.50					0.00	0.00
UIL.21.13441	EXTRA PEPPERMINT	1.05	125.26	0.53	0.11	52.63	0.000	0.00	1.00	0.00	0.00	0.00	0.000	0.00	0.00	0.00	3.00	0.00	0.00	0.00	0.00	0.00	0.00	0.00	0.00
UIL.21.13435	KINDER JOY	8.72	55.23	32.22	15.15	49.62	0.192	8.20	58.00	32.00	15.00	51.00	0.125	6.20	10.20	50.00	66.00	25.60	38.40	12.00	18.00	43.00	59.00	0.00	0.28
UIL.21.13437	KITKAT	8.03	63.82	25.29	13.76	45.37	0.073	7.90	59.90	25.00	13.80	46.00	0.070	5.90	9.90	51.90	67.90	20.00	30.00	11.04	16.56	38.00	54.00	0.00	0.22
UIL.21.13440	MARS MINIS	4.31	73.69	17.92	11.54	57.77	0.222	0.50	9.00	2.00	1.00	8.00	0.016	0.00	2.50	7.00	11.00	0.50	3.50	0.20	1.80	6.00	10.00	0.00	0.17
UIL.21.13444	MENTOS SUGARFREE CHEWING GUM	1.14	93.14	0.57	0.11	32.00	0.000	0.00	1.30	0.00	0.00	0.00	0.000	0.00	0.00	0.00	3.30	0.00	0.00	0.00	0.00	0.00	0.00	0.00	0.00
UIL.21.14391	Nutella hazelnut spread with cocoa	5.40	60.98	31.20	10.53	57.94	0.044	6.30	61.00	31.00	10.60	56.00	0.042	4.30	8.30	53.00	69.00	24.80	37.20	8.48	12.72	48.00	64.00	0.00	0.19
UIL.21.13438	SNICKERS MINIS	9.27	57.73	26.80	6.73	52.53	0.263	1.20	9.00	4.00	2.00	8.00	0.033	0.00	3.20	7.00	11.00	2.50	5.50	1.20	2.80	6.00	10.00	0.00	0.18
UIL.21.13454	Twix minis	4.85	68.95	25.35	11.45	48.60	0.186	0.90	14.00	5.00	3.00	10.00	0.032	0.00	2.90	11.20	16.80	3.50	6.50	2.20	3.80	8.00	12.00	0.00	0.18
UIL.21.13443	WRIGLEYS DOUBLEMINT CHEWING GUM	0.77	91.54	0.38	0.08	81.54	0.000	0.00	2.00	0.00	0.00	2.00	0.000	0.00	0.00	0.00	4.00	0.00	0.00	0.00	0.00	0.00	4.00	0.00	0.00
UIL.21.14384	Al Mudhish puffed corn cheese balls	6.42	55.17	35.75	16.58	4.42	0.621	0.60	7.00	4.20	1.80	0.30	0.073	0.00	2.60	5.00	9.00	2.70	5.70	1.00	2.60	0.00	2.30	0.00	0.22
UIL.21.13433	ALRIFAI MIXED NUTS	21.39	40.00	32.43	4.64	7.18	0.240	5.00	11.00	9.00	1.00	2.00	0.064	3.00	7.00	8.80	13.20	7.50	10.50	0.20	1.80	0.00	4.00	0.00	0.21
UIL.21.13431	AMERICAN GARDEN POPCORN BUTTER	7.70	55.15	24.42	10.91	0.30	0.985	3.00	19.00	8.00	4.00	0.00	0.320	1.00	5.00	15.20	22.80	6.50	9.50	3.20	4.80	0.00	0.00	0.17	0.47
UIL.21.13432	BEST SALTED MIXED NUTS	24.52	19.56	51.89	8.31	4.28	0.336	24.20	19.40	51.70	8.00	4.40	0.320	19.36	29.04	15.52	23.28	43.70	59.70	6.40	9.60	2.40	6.40	0.17	0.47
UIL.21.17408	Cheese balls (Mr. Krispy)	6.80	61.87	34.33	7.67	7.47	1.542	1.00	9.00	5.00	1.00	1.00	0.220	0.00	3.00	7.00	11.00	3.50	6.50	0.20	1.80	0.00	3.00	0.07	0.37
UIL.21.14418	Cheese balls(pofak oman)	9.89	46.98	39.15	16.79	4.17	0.589	9.63	48.20	28.80			0.590	7.63	11.63	40.20	56.20	23.04	34.56					0.47	0.71
UIL.21.14551	Cheese pastry (Dhahabi))	11.57	41.35	18.54	4.74	3.85	0.307	10.59	42.61	18.24	4.20	2.22	0.307	8.47	12.71	34.61	50.61	14.59	21.89	3.36	5.04	0.22	4.22	0.16	0.46
UIL.21.13463	Cheetos /Flamin Hot	5.93	59.57	34.63	9.73	1.07	0.701	1.60	16.00	10.00	2.90	0.30	0.203	0.00	3.60	12.80	19.20	8.00	12.00	2.10	3.70	0.00	2.30	0.05	0.35
UIL.21.14386	chips Oman	6.54	47.63	38.77	16.89	0.00	0.503	5.00		73.00			0.500	3.00	7.00			65.00	81.00					0.40	0.60
UIL.21.14383	Lay's (salt)	7.50	53.71	35.50	16.07	0.00	0.594	1.00	6.70	5.00	2.20	0.10	0.082	0.00	3.00	4.70	8.70	3.50	6.50	1.40	3.00	0.00	2.10	0.00	0.23
UIL.21.14390	Mani (mixed Nuts)	26.01	18.95	49.33	7.92	8.55	0.316	24.50	21.70	49.00	7.50	4.60	0.315	19.60	29.40	17.36	26.04	41.00	57.00	6.00	9.00	2.60	6.60	0.17	0.47
UIL.21.13458	Nabil cracker salted biscuits	9.58	65.90	19.26	13.50	20.04	0.999	9.40	65.90	19.10	12.60	20.20	0.993	7.40	11.40	57.90	73.90	15.28	22.92	10.08	15.12	16.16	24.24	0.79	1.19
UIL.21.14389	Popcorn cheese / American garden	6.58	48.52	34.21	15.67	0.00	0.989	2.00	16.00	11.00	5.50	0.00	0.320	0.00	4.00	12.80	19.20	8.80	13.20	4.40	6.60	0.00	0.00	0.17	0.47
UIL.21.14385	potato chips ready salted	7.11	52.14	36.11	15.75	0.00	0.536	1.90	15.00	10.00	4.50	0.06	0.140	0.00	3.90	12.00	18.00	8.00	12.00	3.60	5.40	0.00	2.06	0.00	0.29
UIL.21.13462	Pringles original	4.53	58.06	33.49	3.53	1.05	0.453	4.30	55.00	33.00	3.10	1.20	0.440	2.30	6.30	47.00	63.00	26.40	39.60	2.30	3.90	0.00	3.20	0.29	0.59
UIL.21.14388	Shai (mix Nut)	25.21	20.69	48.44	6.87	6.85	0.151																		
UIL.21.14560	Sohar chips	3.18	60.81	32.36	14.31	0.00	0.951	2.53	60.72	31.71			0.950	0.53	4.53	52.72	68.72	25.37	38.05					0.76	1.14
UIL.21.13833	vanilla icecream (Igloo)	3.74	20.53	11.13	6.68	18.60	0.068	2.00	12.00	4.00	3.00	11.00	0.034	0.00	4.00	9.60	14.40	2.50	5.50	2.20	3.80	8.80	13.20	0.00	0.18
UIL.21.13832	vanilla icecream (Mazoon)	3.09	13.73	7.66	4.46	12.37	0.046	2.00	13.00	7.00	4.50	8.00	0.040	0.00	4.00	10.40	15.60	5.50	8.50	3.60	5.40	6.00	10.00	0.00	0.19
UIL.21.13300	KELLOGGS Coco Pops	9.54	80.62	2.48	1.15	24.48	0.000	9.00	75.00	2.50	0.90	27.00	0.312	7.00	11.00	67.00	83.00	1.00	4.00	0.10	1.70	21.60	32.40	0.16	0.46
UIL.21.14387	Kelloggs special (cereal)	18.26	72.92	2.21	0.59	13.92	0.696	7.00	29.00	0.50	0.00	5.00	0.270	5.00	9.00	23.20	34.80	0.00	2.00	0.00	0.00	3.00	7.00	0.12	0.42

id_lab	Name of Product & Brand	Amounts per 100 g/ml from Laboratory analyses						Amounts per 100 g/ml from Labelling						Tolerances for the nutrition declaration on food products											
														Prot (g)		Carb (g)		TF (g)		SFA (g)		Sugars (g)		Na (g)	
		Prot[1] (g)	Carb[2] (g)	TF[3] (g)	SFA[4] (g)	Sugars (g)	Na5 (g)	Prot (g)	Carb (g)	TF (g)	SFA (g)	Sugars (g)	Na (g)	Low	High	Low	High	Low	High	Low	High	Low	High	Low	High
UIL.21.13299	NESTLE Chocapic	8.76	82.06	4.79	1.50	24.95	0.102	8.60	73.30	4.60	1.30	24.90	0.090	6.60	10.60	65.30	81.30	3.10	6.10	0.50	2.10	19.92	29.88	0.00	0.24
UIL.21.13298	NESTLE Gold Corn flakes	7.24	84.19	1.96	0.38	10.57	0.426	7.10	79.90	1.90	0.40	10.70	0.430	5.10	9.10	71.90	87.90	0.40	3.40	0.00	1.20	8.56	12.84	0.28	0.58
UIL.21.14574	7 days mini croissant	9.45	44.27	28.55	16.73	16.82	0.203	1.00	5.00	3.00	1.00	2.00	0.022	0.00	3.00	3.00	7.00	1.50	4.50	0.20	1.80	0.00	4.00	0.00	0.17
UIL.21.13460	Americana premium butter cookies	6.17	70.51	20.27	9.16	29.40	0.113	6.00	70.00	20.00	8.14	30.00	0.110	4.00	8.00	62.00	78.00	16.00	24.00	6.51	9.77	24.00	36.00	0.00	0.26
UIL.21.14552	Apple pastry	5.91	48.89	15.74	8.37	16.32	0.012	6.54	48.71	15.26	8.10	15.14	0.012	4.54	8.54	40.71	56.71	12.21	18.31	6.48	9.72	12.11	18.17	0.00	0.16
UIL.21.17405	Apple puff (Lusine)	8.90	47.91	13.10	5.89	19.66	0.275	6.00	33.00	9.00	4.00	13.00	0.187	4.00	8.00	26.40	39.60	7.50	10.50	3.20	4.80	10.40	15.60	0.04	0.34
UIL.21.14566	Atyab twin cake vanilla	5.82	56.21	18.43	7.17	27.82	0.222	4.10	55.80	17.50	8.30	26.50	0.213	2.10	6.10	47.80	63.80	14.00	21.00	6.64	9.96	21.20	31.80	0.06	0.36
UIL.21.13451	Britannia chocolate flavoured premium crème wafer	6.88	64.50	25.38	15.75	33.81	0.248	7.00	63.00	25.00	22.00	35.00	0.235	5.00	9.00	55.00	71.00	20.00	30.00	17.60	26.40	28.00	42.00	0.09	0.39
UIL.21.17406	Cheese puff (Lusine)	10.17	36.17	22.03	10.30	8.77	0.616	7.00	25.00	15.00	7.00	6.00	0.422	5.00	9.00	20.00	30.00	12.00	18.00	5.60	8.40	4.00	8.00	0.27	0.57
UIL.21.14568	Chocolate jumbo croissant	7.75	44.45	23.69	13.05	12.08	0.200	8.31	42.94	23.05	11.25	10.52	0.191	6.31	10.31	34.94	50.94	18.44	27.66	9.00	13.50	8.42	12.62	0.04	0.34
UIL.21.17531	Croissant (Dhahabi)	7.21	50.11	23.18	10.21	11.95	0.622	6.90	49.40	22.90	10.00	11.60	0.611	4.90	8.90	41.40	57.40	18.32	27.48	8.00	12.00	9.28	13.92	0.49	0.73
UIL.21.13461	Danish Butter Cookies	5.52	64.88	25.44	18.34	26.04	0.126	5.40	65.00	25.00	17.00	26.00	0.124	3.40	7.40	57.00	73.00	20.00	30.00	13.60	20.40	20.80	31.20	0.00	0.27
UIL.21.17407	Donut (Switz)	6.63	55.09	17.57	5.57	19.74	0.241	2.00	19.00	6.00	1.50	6.00	0.080	0.00	4.00	15.20	22.80	4.50	7.50	0.70	2.30	4.00	8.00	0.00	0.23
UIL.21.13457	Lotus biscoff	5.64	73.72	19.36	13.59	36.41	0.413	0.40	5.70	1.50	0.60	3.00	0.029	0.00	2.40	3.70	7.70	0.00	3.00	0.00	1.40	1.00	5.00	0.00	0.18
UIL.21.13446	Nabil cream wafers (chocolate flavour)	5.61	70.25	21.29	12.74	20.63	0.147	5.40	69.25	21.14	18.25	20.66	0.120	3.40	7.40	61.25	77.25	16.91	25.37	14.60	21.90	16.53	24.79	0.00	0.27
UIL.21.13445	Nabil glucose	9.30	73.57	14.36	7.42	14.88	0.000	9.10	72.90	14.20	8.90	15.00	0.128	7.10	11.10	64.90	80.90	11.36	17.04	7.12	10.68	12.00	18.00	0.00	0.28
UIL.21.13447	Nabil Nice (sugar sprinkled coconut biscuits)	9.52	71.75	15.36	7.12	21.16	0.375	9.00	71.80	15.00	9.70	22.00	0.370	7.00	11.00	63.80	79.80	12.00	18.00	7.76	11.64	17.60	26.40	0.22	0.52
UIL.21.13452	Nutro chocolate flavoured cream wafers	3.50	62.53	30.20	26.80	36.17	0.204	5.00	62.00	29.00	23.00	38.00	0.160	3.00	7.00	54.00	70.00	23.20	34.80	18.40	27.60	30.40	45.60	0.01	0.31
UIL.21.13459	Original Oreo	5.32	71.58	19.47	8.79	42.11	0.582	1.00	13.00	3.70	1.60	8.00	0.105	0.00	3.00	10.40	15.60	2.20	5.20	0.80	2.40	6.00	10.00	0.00	0.26
UIL.21.14567	Switz Mini cupcake vanilla	7.13	48.59	29.41	12.75	29.00	0.404	2.00	15.00	9.00	4.00	9.00	0.120	0.00	4.00	12.00	18.00	7.50	10.50	3.20	4.80	7.00	11.00	0.00	0.27
UIL.21.13453	tiffany Crunch and cream	8.65	64.63	24.36	21.51	36.23	0.120	8.40	59.80	24.00	19.40	36.40	0.116	6.40	10.40	51.80	67.80	19.20	28.80	15.52	23.28	29.12	43.68	0.00	0.27
UIL.21.13449	TIFFANY Everyday NICE (Sugar sprinkled coconut biscuits)	6.45	71.29	20.32	9.16	25.48	0.355	2.10	21.50	6.30	3.70	8.00	0.069	0.10	4.10	17.20	25.80	4.80	7.80	2.90	4.50	6.00	10.00	0.00	0.22
UIL.21.13448	Tiffany Glucose (milk and honey biscuits)	7.42	72.26	17.84	9.16	22.90	0.356	2.20	22.20	5.50	2.90	7.20	0.108	0.20	4.20	17.76	26.64	4.00	7.00	2.10	3.70	5.20	9.20	0.00	0.26
UIL.21.17403	ACTIVIA FULL FAT FRESH LABAN	3.20	4.65	3.28	2.26	4.46	0.052	3.00	4.60	3.60	2.30	4.60	0.048	1.00	5.00	2.60	6.60	2.10	5.10	1.50	3.10	2.60	6.60	0.00	0.20
UIL.21.17409	ACTIVIA FULL FAT PLAIN YOGHURT	2.77	3.75	2.08	1.53	3.32	0.035	4.00	5.40	3.00	2.00	5.40	0.049	2.00	6.00	3.40	7.40	1.50	4.50	1.20	2.80	3.40	7.40	0.00	0.20
UIL.21.13544	ALMARAI FRESH LABAN FULL FAT	3.07	4.33	3.64	2.57	4.09	0.055	5.00	8.00	6.00	4.00	8.00	0.097	3.00	7.00	6.00	10.00	4.50	7.50	3.20	4.80	6.00	10.00	0.00	0.25
UIL.21.13545	ALMARAI LABAN UP	2.71	4.27	1.15	0.81	3.99	0.316	5.00	7.00	2.00	1.00	7.00	0.621	3.00	7.00	5.00	9.00	0.50	3.50	0.20	1.80	5.00	9.00	0.50	0.75
UIL.21.13548	ALMARAI STRAWBERRY LABAN	3.52	15.40	1.36	0.91	15.29	0.056	11.00	51.00	4.00	3.00	51.00	0.181	8.80	13.20	43.00	59.00	2.50	5.50	2.20	3.80	43.00	59.00	0.03	0.33
UIL.21.13546	AlRawabi Up:Laban Drink	2.15	3.23	1.79	1.20	3.04	0.000	1.97	2.76	1.63	0.77	2.76	0.000	0.00	3.97	0.76	4.76	0.13	3.13	0.00	1.57	0.76	4.76	0.00	0.00
UIL.21.17404	BALADE FARMS AYRAN ORIGINAL LABAN	1.65	3.40	1.27	1.02	0.00	0.375	1.60	3.27	1.18			0.370	0.00	3.60	1.27	5.27	0.00	2.68					0.22	0.52
UIL.21.14131	Cream (Luna)	2.03	3.97	21.71	16.71	3.10	0.000	3.00	3.00	21.00	16.57	2.90	0.000	1.00	5.00	1.00	5.00	16.80	25.20	13.26	19.88	0.90	4.90	0.00	0.00
UIL.21.14553	cream (Puck)	2.65	2.88	24.51	11.62	0.00	0.003	2.90	3.50	23.00	11.00	3.40	0.049	0.90	4.90	1.50	5.50	18.40	27.60	8.80	13.20	1.40	5.40	0.00	0.20
UIL.21.13539	Fresh laban (Alrawabi)	3.28	4.62	3.27	2.29	3.97	0.000	3.00	4.60	3.20				1.00	5.00	2.60	6.60	1.70	4.70						
UIL.21.13594	Fresh Labnah:Full Fat Mazoon	6.54	7.16	11.13	7.82	6.63	0.214	6.00	8.00	11.00	8.30	7.20	0.200	4.00	8.00	6.00	10.00	8.80	13.20	6.64	9.96	5.20	9.20	0.05	0.35
UIL.21.13592	Fresh Labnah; Full Fat Al-Marai	5.91	8.85	12.85	8.82	7.33	0.224	3.00	4.00	7.00	5.00	4.00	0.110	1.00	5.00	2.00	6.00	5.50	8.50	4.00	6.00	2.00	6.00	0.00	0.26
UIL.21.13558	Fresh Yougurt Full Fat: Al-Marai	4.23	5.75	3.61	2.57	5.62	0.071	7.00	11.00	6.00	3.00	11.00	0.115	5.00	9.00	8.80	13.20	4.50	7.50	2.20	3.80	8.80	13.20	0.00	0.27
UIL.21.13556	Fresh Yougurt Full Fat: Al-Safwah	3.56	5.82	3.23	2.01	4.78	0.072	3.20	8.70	2.10			0.065	1.20	5.20	6.70	10.70	0.60	3.60					0.00	0.22

id_lab	Name of Product & Brand	Amounts per 100 g/ml from Laboratory analyses						Amounts per 100 g/ml from Labelling						Tolerances for the nutrition declaration on food products											
														Prot (g)		Carb (g)		TF (g)		SFA (g)		Sugars (g)		Na (g)	
		Prot[1] (g)	Carb[2] (g)	TF[3] (g)	SFA[4] (g)	Sugars (g)	Na5 (g)	Prot (g)	Carb (g)	TF (g)	SFA (g)	Sugars (g)	Na (g)	Low	High	Low	High	Low	High	Low	High	Low	High	Low	High
UIL.21.13549	Laban drink (Unikai)	3.19	3.29	0.74	0.48	3.02	1.876	2.50	3.50	0.70				0.50	4.50	1.50	5.50	0.00	2.20						
UIL.21.14179	Laban drinks (Alkhamayil)	1.94	4.22	0.58	0.37	0.89	0.000	0.03	0.03	0.01			0.003	0.00	2.03	0.00	2.03	0.00	1.51					0.00	0.15
UIL.21.13543	Laban fresh full creeam (Asafwah)	3.31	5.10	3.31	2.37	4.07	0.069	3.30	4.50	3.10	2.20	4.50	0.064	1.30	5.30	2.50	6.50	1.60	4.60	1.40	3.00	2.50	6.50	0.00	0.21
UIL.21.13541	Laban up (Alsafwa)	2.60	3.96	1.55	1.04	3.89	0.341	2.40	3.80	1.40	0.95	3.80	0.333	0.40	4.40	1.80	5.80	0.00	2.90	0.15	1.75	1.80	5.80	0.18	0.48
UIL.21.13593	Labneh Turkish Reciepe: Puck	8.41	6.92	14.21	9.82	6.43	0.312	8.00	8.00	14.00	10.00	4.00	0.298	6.00	10.00	6.00	10.00	11.20	16.80	8.00	12.00	2.00	6.00	0.15	0.45
UIL.21.13542	MAZOON FRESH LABAN FULL FAT	4.09	4.64	3.30	2.22	4.48	0.052	3.80	4.40	3.00	1.70	4.40	0.050	1.80	5.80	2.40	6.40	1.50	4.50	0.90	2.50	2.40	6.40	0.00	0.20
UIL.21.13555	MAZOON FRESH YOGHURT FULL FAT	3.41	6.38	3.21	2.29	5.04	0.058	3.80	5.70	3.00	1.70	5.70	0.050	1.80	5.80	3.70	7.70	1.50	4.50	0.90	2.50	3.70	7.70	0.00	0.20
UIL.21.13540	MAZOON LABAN UP	1.92	3.70	0.89	0.60	3.70	0.345	1.60	2.20	0.80	0.60	0.00	0.340	0.00	3.60	0.20	4.20	0.00	2.30	0.00	1.40	0.00	0.00	0.19	0.49
UIL.21.14180	MAZOON SAMBHARAM SPICY LABAN DRINK	1.94	3.33	0.85	0.54	1.68	0.355	1.60	2.20	0.80	0.60	0.00	0.340	0.00	3.60	0.20	4.20	0.00	2.30	0.00	1.40	0.00	0.00	0.19	0.49
UIL.21.13547	MAZOON STRAWBERRY LABAN	3.28	14.37	3.49	2.30	14.27	0.054	3.00	14.00	3.00	2.20	14.00	0.050	1.00	5.00	11.20	16.80	1.50	4.50	1.40	3.00	11.20	16.80	0.00	0.20
UIL.21.13830	Nestle cream	3.09	4.90	23.44	13.40	0.00	0.000	2.60		23.10	15.30			0.60	4.60			18.48	27.72	12.24	18.36				
UIL.21.13557	Yoghurt:Full Cream UNIKAI	3.52	4.83	3.19	2.41	4.41	0.083	3.40	5.10	3.00			0.000	1.40	5.40	3.10	7.10	1.50	4.50					0.00	0.00
UIL.21.13559	Youghurt;Full Cream AlRawabi	4.31	5.44	3.31	2.37	4.85	0.086	4.20	6.40	3.20				2.20	6.20	4.40	8.40	1.70	4.70						
UIL.21.13840	ALMARAI BURGER SLICE CHEESE	15.20	5.75	25.30	15.15	0.00	1.378	3.00	1.00	5.00	3.00	0.00	0.261	1.00	5.00	0.00	3.00	3.50	6.50	2.20	3.80	0.00	0.00	0.11	0.41
UIL.21.13845	ALMARAI CREAM CHEESE	5.20	4.27	34.10	25.13	0.00	0.745	1.60	1.00	10.00	6.00	0.70	0.216	0.00	3.60	0.00	3.00	8.00	12.00	4.80	7.20	0.00	2.70	0.07	0.37
UIL.21.13844	ALMARAI CREAM CHEESE CHEDDAR	10.23	4.53	30.87	15.50	0.00	1.152	3.00	1.00	9.00	6.00	1.00	0.341	1.00	5.00	0.00	3.00	7.50	10.50	4.80	7.20	0.00	3.00	0.19	0.49
UIL.21.14572	Chedder cheese (Almarai)	17.90	0.00	24.97	16.77	0.00	1.899	5.00	0.00	7.00	5.00	0.00	0.564	3.00	7.00	0.00	0.00	5.50	8.50	4.00	6.00	0.00	0.00	0.45	0.68
UIL.21.14571	Chedder cheese (Kraft)	13.56	5.64	25.56	17.84	0.00	1.800	16.00	2.40	24.00	15.00	0.20	1.728	12.80	19.20	0.40	4.40	19.20	28.80	12.00	18.00	0.00	2.20	1.38	2.07
UIL.21.13837	cheese triangles Al Maraai	10.37	3.50	27.30	17.87	0.00	0.952	3.00	1.00	8.00	4.00	0.00	0.274	1.00	5.00	0.00	3.00	6.50	9.50	3.20	4.80	0.00	0.00	0.12	0.42
UIL.21.13838	cheese triangles Puck	8.47	5.83	25.52	12.95	0.00	1.099	7.20	5.20	25.00	13.00	0.10	1.018	5.20	9.20	3.20	7.20	20.00	30.00	10.40	15.60	0.00	2.10	0.81	1.22
UIL.21.13835	Haloumi Pinar	24.32	3.95	25.87	14.12	0.00	1.896	24.00	2.00	25.00	14.00	1.90	1.800	19.20	28.80	0.00	4.00	20.00	30.00	11.20	16.80	0.00	3.90	1.44	2.16
UIL.21.13834	Haloumi Cheese Al-Marai	23.87	3.53	27.10	24.20	0.00	1.254	7.00	1.00	8.00	5.00	0.00	0.360	5.00	9.00	0.00	3.00	6.50	9.50	4.00	6.00	0.00	0.00	0.21	0.51
UIL.21.13848	KRAFT CHEDDAR CHEESE SPREAD ORIGINAL	8.34	4.21	28.21	19.12	0.00	1.216	7.80	3.00	27.00	19.00	2.80	1.116	5.80	9.80	1.00	5.00	21.60	32.40	15.20	22.80	0.80	4.80	0.89	1.34
UIL.21.13839	KRAFT SLICE CHEESE ORIGINAL	15.14	7.43	21.56	13.17	0.00	1.356	14.90	7.00	20.60	13.40	3.90	1.298	11.92	17.88	5.00	9.00	16.48	24.72	10.72	16.08	1.90	5.90	1.04	1.56
UIL.21.13843	MAZOON SPREAD CHEESE	6.72	3.08	33.48	24.12	0.00	0.396	6.00	2.00	33.00	22.40	0.00	0.385	4.00	8.00	0.00	4.00	26.40	39.60	17.92	26.88	0.00	0.00	0.24	0.54
UIL.21.13842	MAZOON SPREAD CHEESE CHEDDAR	7.12	2.36	33.52	23.82	0.00	0.395	6.00	2.00	33.00	22.40	0.00	0.385	4.00	8.00	0.00	4.00	26.40	39.60	17.92	26.88	0.00	0.00	0.24	0.54
UIL.21.14554	Pinar processed cream cheese spread	6.66	1.61	31.02	27.80	0.00	0.709	8.40	1.70	30.00	15.80	1.70	0.700	6.40	10.40	0.00	3.70	24.00	36.00	12.64	18.96	0.00	3.70	0.56	0.84
UIL.21.13847	PUCK CREAM CHEESE	8.65	2.49	31.85	20.97	0.00	0.836	8.70	1.90	31.00	19.00	1.90	0.824	6.70	10.70	0.00	3.90	24.80	37.20	15.20	22.80	0.00	3.90	0.66	0.99
UIL.21.13846	PUCK CREAM CHEESE CHEDDAR	8.52	2.62	32.01	19.20	0.00	0.796	8.70	1.90	31.00	19.00	1.90	0.786	6.70	10.70	0.00	3.90	24.80	37.20	15.20	22.80	0.00	3.90	0.63	0.94
UIL.21.13841	PUCK SLICE CHEESE ORIGINAL	14.81	5.68	27.66	14.04	0.00	0.609	14.00	5.50	27.00	14.00	5.50	0.600	11.20	16.80	3.50	7.50	21.60	32.40	11.20	16.80	3.50	7.50	0.48	0.72
UIL.21.13836	Triangle cheese Abu Alwald	8.22	8.11	21.02	11.92	0.00	0.745	7.50	7.00	20.50	12.00	5.50	0.720	5.50	9.50	5.00	9.00	16.40	24.60	9.60	14.40	3.50	7.50	0.58	0.86
UIL.21.13456	Britannia toastea wheat Rusk	9.61	77.48	9.74	4.35	26.71	0.223	2.00	25.00	3.00	2.00	8.50	0.065	0.00	4.00	20.00	30.00	1.50	4.50	1.20	2.80	6.50	10.50	0.00	0.22
UIL.21.13455	Tiffany whole wheat Rusks	10.13	76.93	8.49	3.80	23.11	0.225	10.00	70.00	8.40	4.00	23.20	0.219	8.00	12.00	62.00	78.00	6.90	9.90	3.20	4.80	18.56	27.84	0.07	0.37
UIL.21.13429	INDOMIE FRIED NOODLES	8.54	62.06	20.92	11.35	8.24	1.364	7.20	61.90	20.90	16.30	8.30	1.050	5.20	9.20	53.90	69.90	16.72	25.08	13.04	19.56	6.30	10.30	0.84	1.26
UIL.21.14202	Al-Islami Ground Beef	11.74	2.34	9.25	4.16	1.38	0.421	11.90	1.90	8.60				9.52	14.28	0.00	3.90	7.10	10.10						
UIL.21.14203	Al-Kabeer Jumbo Hotdogs	17.12	4.25	7.69	4.31	0.00	0.540	17.00	3.00	7.00	3.00	0.70	0.530	13.60	20.40	1.00	5.00	5.50	8.50	2.20	3.80	0.00	2.70	0.42	0.64
UIL.21.14194	Al-Safa Breaded Chiocken Finger	18.80	19.68	8.31	2.56	0.00	0.486	15.50	20.20	7.90	2.30	1.50	0.472	12.40	18.60	16.16	24.24	6.40	9.40	1.50	3.10	0.00	3.50	0.32	0.62
UIL.21.14196	Al-Safa Chicken Burger	16.86	3.94	10.41	3.01	0.00	0.395	16.00	4.60	9.40	2.80	0.80	0.383	12.80	19.20	2.60	6.60	7.90	10.90	2.00	3.60	0.00	2.80	0.23	0.53

id_lab	Name of Product & Brand	Amounts per 100 g/ml from Laboratory analyses						Amounts per 100 g/ml from Labelling						Tolerances for the nutrition declaration on food products											
														Prot (g)		Carb (g)		TF (g)		SFA (g)		Sugars (g)		Na (g)	
		Prot[1] (g)	Carb[2] (g)	TF[3] (g)	SFA[4] (g)	Sugars (g)	Na5 (g)	Prot (g)	Carb (g)	TF (g)	SFA (g)	Sugars (g)	Na (g)	Low	High	Low	High	Low	High	Low	High	Low	High	Low	High
UIL.21.14205	Al-Safa Chicken Franks	12.29	5.85	14.88	8.06	0.00	1.301	4.00	2.00	5.00			0.440	2.00	6.00	0.00	4.00	3.50	6.50					0.29	0.59
UIL.21.14204	Americana Beef Hotdog	15.05	2.11	12.56	5.27	0.00	0.603	14.00	2.00	12.00	4.50	1.00	0.600	11.20	16.80	0.00	4.00	9.60	14.40	3.60	5.40	0.00	3.00	0.48	0.72
UIL.21.14200	Beef burger (Taybat)	19.11	6.40	13.52	6.99	0.00	0.621	19.20	7.70	12.90	6.00	1.40	0.619	15.36	23.04	5.70	9.70	10.32	15.48	4.80	7.20	0.00	3.40	0.50	0.74
UIL.21.14199	Beef Burger Sadia	14.21	6.70	20.57	10.32	0.00	0.456	14.00	6.50	20.00	11.50	0.55		11.20	16.80	4.50	8.50	16.00	24.00	9.20	13.80	0.00	2.55		
UIL.21.14201	corned Beef (Taybat)	12.50	12.78	8.42	4.14	0.54	0.211	6.00	6.00	4.00				4.00	8.00	4.00	8.00	2.50	5.50						
UIL.21.14573	FAMILY CORNED BEEF	24.87	5.10	10.59	6.54	4.02	0.426																		
UIL.21.14198	Sadia Chicken Burger	14.21	6.78	15.24	5.93	0.00	0.595	13.50	7.50	14.50	7.50	0.95	0.665	10.80	16.20	5.50	9.50	11.60	17.40	6.00	9.00	0.00	2.95	0.53	0.80
UIL.21.14206	Sadia Chicken Franks	12.94	2.70	15.65	6.77	0.00	0.461	12.00	2.37	15.00	5.40	0.70	0.460	9.60	14.40	0.37	4.37	12.00	18.00	4.32	6.48	0.00	2.70	0.31	0.61
UIL.21.14195	Sadia Chicken Nuggets Crispy	11.89	20.87	10.35	3.21	0.00	0.416	12.00	19.00	10.00	5.00	0.35	0.405	9.60	14.40	15.20	22.80	8.00	12.00	4.00	6.00	0.00	2.35	0.26	0.56
UIL.21.13295	LIGHT CHUNKS TUNA CALIFORNIA GARDEN IN WATER	23.69	0.95	1.07	0.80	0.84	0.215	23.90	0.68	1.16			0.208	19.12	28.68	0.00	2.68	0.00	2.66					0.06	0.36
UIL.21.13291	SKIPJACK TUNA ALALALI IN SUN FLOWER OIL	23.12	3.96	11.02	1.46	0.80	0.480	23.00	0.00	10.60	0.56	0.00	0.472	18.40	27.60	0.00	0.00	8.48	12.72	0.00	1.36	0.00	0.00	0.32	0.62
UIL.21.13294	SKIPJACK TUNA CALIFORNIA GARDEN IN SUN FLOWER OIL	25.06	1.99	2.98	0.36	0.89	0.303	24.80	1.05	2.67			0.282	19.84	29.76	0.00	3.05	1.17	4.17					0.13	0.43
UIL.21.13292	TUNA ALALALI IN WATER	23.19	3.29	0.01	0.00	0.89	0.480	23.00	0.00	0.00	0.00	0.00	0.472	18.40	27.60	0.00	0.00	0.00	0.00	0.00	0.00	0.00	0.00	0.32	0.62
UIL.21.13434	WHITE TUNA MEAT IN BRINE	25.57	1.97	0.61	0.46	0.10	0.381	23.00	0.90	0.90	0.40	0.00	0.400	18.40	27.60	0.00	2.90	0.00	2.40	0.00	1.20	0.00	0.00	0.25	0.55
UIL.21.13293	WHITE TUNA MEAT IN SUN FLOWER OIL DELMONTE	24.75	2.96	5.58	0.80	0.36	0.425	24.00	2.00	4.60	0.70	0.00	0.400	19.20	28.80	0.00	4.00	3.10	6.10	0.00	1.50	0.00	0.00	0.25	0.55
UIL.21.13282	BAKED BEANS ALMAZRAA	4.40	25.97	0.63	0.16	6.12	0.392	6.00	25.00	1.00	0.00	8.00	0.500	4.00	8.00	20.00	30.00	0.00	2.50	0.00	0.00	6.00	10.00	0.40	0.60
UIL.21.13278	BAKED BEANS CALIFORNIA GARDEN	5.20	12.42	1.20	0.38	10.40	0.313	7.00	20.00	1.50	0.50	14.00	0.390	5.00	9.00	16.00	24.00	0.00	3.00	0.00	1.30	11.20	16.80	0.24	0.54
UIL.21.13289	BOILDCHICK PEAS MARA	4.11	11.37	1.31	0.26	0.74	0.154	3.80	9.00	1.50	0.20	0.00	0.152	1.80	5.80	7.00	11.00	0.00	3.00	0.00	1.00	0.00	0.00	0.00	0.30
UIL.21.13286	CHICK PEAS ALMAZRAA	6.04	16.14	0.61	0.11	0.81	0.294	8.00	19.00	1.00	0.00	1.00	0.370	6.00	10.00	15.20	22.80	0.00	2.50	0.00	0.00	0.00	3.00	0.22	0.52
UIL.21.13281	CHICK PEAS CALIFORNIA GARDEN	7.12	19.98	0.64	0.13	0.84	0.267	10.00	17.00	1.00	0.00	2.00	0.340	8.00	12.00	13.60	20.40	0.00	2.50	0.00	0.00	0.00	4.00	0.19	0.49
UIL.21.13284	CHICK PEAS LUNA	4.71	16.44	1.60	0.74	0.76	0.346	5.00	21.30	1.90	0.80	0.60	0.300	3.00	7.00	17.04	25.56	0.40	3.40	0.00	1.60	0.00	2.60	0.15	0.45
UIL.21.14397	diamond strawberry jam	0.39	66.47	0.31	0.27	63.84	0.015	0.40	65.00	0.30				0.00	2.40	57.00	73.00	0.00	1.80						
UIL.21.13283	FOUL MEDAMAS ALMAZRAA	5.18	15.11	0.01	0.00	0.77	0.434	7.00	18.00	0.00	0.00	2.00	0.550	5.00	9.00	14.40	21.60	0.00	0.00	0.00	0.00	0.00	4.00	0.44	0.66
UIL.21.14557	Foul Medammas (American Garden)	6.83	16.53	0.01	0.00	1.22	0.341	8.00	22.00	0.00	0.00	1.00	0.430	6.00	10.00	17.60	26.40	0.00	0.00	0.00	0.00	0.00	3.00	0.28	0.58
UIL.21.13276	GREEN GIANT SWEET CORN	2.01	15.16	1.63	0.38	7.48	0.204	1.90	9.40	1.40	0.30	5.90	0.128	0.00	3.90	7.40	11.40	0.00	2.90	0.00	1.10	3.90	7.90	0.00	0.28
UIL.21.13285	GREEN PEAS LUNA	4.27	16.41	0.42	0.12	0.61	0.237	4.50	16.00	0.50	0.25	0.40	0.200	2.50	6.50	12.80	19.20	0.00	2.00	0.00	1.05	0.00	2.40	0.05	0.35
UIL.21.14555	LA MING beans in tomato sauce	7.64	88.34	0.98	0.23	2.40	0.000																		
UIL.21.14556	LUNA baked beans in tomato sauce	7.07	14.44	0.30	0.19	3.96	0.320	5.86	16.00	0.36	0.20	3.90	0.300	3.86	7.86	12.80	19.20	0.00	1.86	0.00	1.00	1.90	5.90	0.15	0.45
UIL.21.14558	LUNAFOUL MEDAMES extra grade fava beans	7.30	16.68	0.41	0.21	1.17	0.330	6.50	16.80	0.20	0.08	0.60	0.300	4.50	8.50	13.44	20.16	0.00	1.70	0.00	0.88	0.00	2.60	0.15	0.45
UIL.21.14406	Mango pickle / Aeroplane	1.46	9.62	4.08	1.23	0.10	5.040	1.40	9.60	3.70	0.90	0.00	5.600	0.00	3.40	7.60	11.60	2.20	5.20	0.10	1.70	0.00	0.00	4.48	6.72
UIL.21.14403	Mango pickles in oil / Ahmed	3.05	9.35	20.80	2.30	0.50	3.016	0.40	2.00	4.00	0.60	0.00	0.600	0.00	2.40	0.00	4.00	2.50	5.50	0.00	1.40	0.00	0.00	0.48	0.72
UIL.21.14405	Mango thokku pickle in oil / Priya	4.07	28.04	4.21	1.03	22.40	4.471																		
UIL.21.14559	Peas processed	3.99	17.19	0.01	0.00	2.46	0.215	6.00	21.00	0.00	0.00	2.00	0.270	4.00	8.00	16.80	25.20	0.00	0.00	0.00	0.00	0.00	4.00	0.12	0.42
UIL.21.13277	PLAIN MEDAMMES CALIFORNIA GARDEN	6.02	17.51	0.01	0.00	0.93	0.343	8.00	22.00	0.00	0.00	1.00	0.430	6.00	10.00	17.60	26.40	0.00	0.00	0.00	0.00	0.00	3.00	0.28	0.58
UIL.21.13287	PROCESSED PEAS ALMAZRAA	4.48	15.42	0.01	0.00	1.64	0.326	6.00	20.00	0.00	0.00	2.00	0.400	4.00	8.00	16.00	24.00	0.00	0.00	0.00	0.00	0.00	4.00	0.25	0.55
UIL.21.13279	PROCESSED PEAS CALIFORNIA GARDEN	4.59	16.37	0.01	0.15	1.64	0.329	6.00	20.00	0.00	0.00	2.00	0.400	4.00	8.00	16.00	24.00	0.00	0.00	0.00	0.00	0.00	4.00	0.25	0.55
UIL.21.13290	SWEET CORN AMERICAN GARDEN	2.50	17.23	1.29	0.29	6.41	0.179	3.00	20.00	1.50	0.00	8.00	0.210	1.00	5.00	16.00	24.00	0.00	3.00	0.00	0.00	6.00	10.00	0.06	0.36

id_lab	Name of Product & Brand	Amounts per 100 g/ml from Laboratory analyses						Amounts per 100 g/ml from Labelling						Tolerances for the nutrition declaration on food products											
														Prot (g)		Carb (g)		TF (g)		SFA (g)		Sugars (g)		Na (g)	
		Prot[1] (g)	Carb[2] (g)	TF[3] (g)	SFA[4] (g)	Sugars (g)	Na5 (g)	Prot (g)	Carb (g)	TF (g)	SFA (g)	Sugars (g)	Na (g)	Low	High	Low	High	Low	High	Low	High	Low	High	Low	High
UIL.21.13280	SWEET CORN CALIFORNIA GARDEN	2.15	19.98	1.08	0.28	3.08	0.133	3.00	18.00	1.50	0.00	4.00	0.170	1.00	5.00	14.40	21.60	0.00	3.00	0.00	0.00	2.00	6.00	0.02	0.32
UIL.21.13288	SWEET CORN LIBBYS	1.68	9.59	0.55	0.16	2.09	0.223	1.00	9.00	0.50	0.00	2.00	0.260	0.00	3.00	7.00	11.00	0.00	2.00	0.00	0.00	0.00	4.00	0.11	0.41
UIL.21.14404	Tomato paste / Alalali	3.54	15.21	0.72	0.38	12.30	0.411	4.30	14.40	0.64	0.00	11.70	0.198	2.30	6.30	11.52	17.28	0.00	2.14	0.00	0.00	9.36	14.04	0.05	0.35
UIL.21.14399	Tomato paste / Almudhish	5.15	16.84	1.02	0.39	10.40	0.000	4.64	17.40	0.80		11.00	0.300	2.64	6.64	13.92	20.88	0.00	2.30			8.80	13.20	0.15	0.45
UIL.21.14400	Tomato paste / Jumbo	2.09	17.76	0.65	0.25	4.14	0.301	1.10	17.20	0.50			0.300	0.00	3.10	13.76	20.64	0.00	2.00					0.15	0.45
UIL.21.14394	american garden BBQ sauce original	0.03	41.64	0.03	0.01	34.14	0.990	0.00	15.00	0.00	0.00	14.00	0.350	0.00	0.00	12.00	18.00	0.00	0.00	0.00	0.00	11.20	16.80	0.20	0.50
UIL.21.14415	Classic Mayonnaise / Mazola	0.07	3.57	79.21	13.64	3.71	0.498	0.00	0.40	11.00	1.50	0.40	0.068	0.00	0.00	0.00	2.40	8.80	13.20	0.70	2.30	0.00	2.40	0.00	0.22
UIL.21.14416	creamy classic mayyonaise / Heinz	0.07	12.14	35.85	7.32	3.46	0.807	0.10	12.40	35.00	5.60	2.70	0.800	0.00	2.10	9.92	14.88	28.00	42.00	4.48	6.72	0.70	4.70	0.64	0.96
UIL.21.14393	Delicio 1000 island	0.86	9.64	36.93	6.64	14.29	1.040	0.10	1.40	5.00	1.00	1.20	0.142	0.00	2.10	0.00	3.40	3.50	6.50	0.20	1.80	0.00	3.20	0.00	0.29
UIL.21.14392	Delicio french dressing	1.07	15.50	36.86	6.64	19.79	1.117	0.10	2.30	5.00	1.00	2.00	0.156	0.00	2.10	0.30	4.30	3.50	6.50	0.20	1.80	0.00	4.00	0.01	0.31
UIL.21.13296	Hayat Hot Sauce	0.63	0.77	0.70	0.00	0.72	2.616																		
UIL.21.14395	Heinz classic BBQ smokey and rich	0.85	33.77	0.35	0.15	28.68	0.496	0.90	34.00	0.20	0.10	29.00	0.480	0.00	2.90	27.20	40.80	0.00	1.70	0.00	0.90	23.20	34.80	0.33	0.63
UIL.21.14402	Hot Sauce / Excellnce	0.33	10.62	0.01	0.00	9.79	2.290																		
UIL.21.14417	Hot sause / American garden	0.60	2.00	0.20	0.04	2.00	2.790	0.00	0.00	0.00	0.00	0.00	0.140	0.00	0.00	0.00	0.00	0.00	0.00	0.00	0.00	0.00	0.00	0.00	0.29
UIL.21.13297	Jumbo Hot Sauce	0.34	0.86	0.10	0.00	0.58	1.610	0.30	0.20	1.04	0.27	0.00	0.306	0.00	2.30	0.00	2.20	0.00	2.54	0.00	1.07	0.00	0.00	0.16	0.46
UIL.21.14563	Knor vegetable stock cubes	0.29	0.44	0.00	0.00	0.00	0.443	0.10	0.40	0.50	0.30	0.20	0.442	0.00	2.10	0.00	2.40	0.00	2.00	0.00	1.10	0.00	2.20	0.29	0.59
UIL.21.14562	Knour beef flavoured stock	0.25	0.52	0.00	0.00	0.00	0.461	0.10	0.40	0.40	0.30	0.10	0.460	0.00	2.10	0.00	2.40	0.00	1.90	0.00	1.10	0.00	2.10	0.31	0.61
UIL.21.14561	Knour CHICKEN stock	0.31	0.37	0.00	0.00	0.00	0.539	0.20	0.30	0.50	0.40	0.00	0.531	0.00	2.20	0.00	2.30	0.00	2.00	0.00	1.20	0.00	0.00	0.42	0.64
UIL.21.14564	Maggi beef flavour	0.00	12.94	0.00	0.00	0.00	19.900	6.90	12.00	20.80	13.20	3.70	18.630	4.90	8.90	9.60	14.40	16.64	24.96	10.56	15.84	1.70	5.70	14.90	22.36
UIL.21.13430	MAGGI CHICKEN STOCK	5.16	14.51	21.89	12.87	8.23	18.220	5.00	13.00	21.80	14.40	8.20	19.100	3.00	7.00	10.40	15.60	17.44	26.16	11.52	17.28	6.20	10.20	15.28	22.92
UIL.21.14565	MAGGI CHICKEN STOCK LESS SALT	0.00	29.10	0.00	0.00	0.00	14.780	5.10	29.70	26.00	14.80	10.90	13.800	3.10	7.10	23.76	35.64	20.80	31.20	11.84	17.76	8.72	13.08	11.04	16.56
UIL.21.14407	Pizza sauce olive& mushrooms / Alalali	2.05	9.65	1.58	0.36	6.38	0.358	2.20	9.50	1.30	0.20	5.30	0.350	0.20	4.20	7.50	11.50	0.00	2.80	0.00	1.00	3.30	7.30	0.20	0.50
UIL.21.14398	Soy sauce classic / American garden	0.60	2.00	0.20	0.04	2.00	2.853	0.00	2.00	0.00	0.00	1.00	0.108	0.00	0.00	0.00	4.00	0.00	0.00	0.00	0.00	0.00	3.00	0.00	0.26
UIL.21.14401	Tabasco hot sauce	1.59	2.84	0.01	0.00	2.65	0.690	1.00	1.60	0.00	0.00	0.10	0.687	0.00	3.00	0.00	3.60	0.00	0.00	0.00	0.00	0.00	2.10	0.55	0.82
UIL.21.14408	Tomato ketchup / Hayat	0.60	26.60	0.07	0.01	22.20	0.816	0.02	4.00	0.00	0.00	3.00	0.120	0.00	2.02	2.00	6.00	0.00	0.00	0.00	0.00	1.00	5.00	0.00	0.27
UIL.21.14413	Tomato ketchup / Heinz	1.40	23.30	0.01	0.00	22.58	0.721	1.20	23.00	0.00	0.00	23.00	0.720	0.00	3.20	18.40	27.60	0.00	0.00	0.00	0.00	18.40	27.60	0.58	0.86
UIL.21.14411	Tomato Ketchup/ Jumbo	0.99	29.35	0.31	0.19	26.94	0.164	1.04	29.27	0.28	0.20	26.88	0.162	0.00	3.04	23.42	35.12	0.00	1.78	0.00	1.00	21.50	32.26	0.01	0.31
UIL.21.13828	Al Deyafa fruit cordial	0.00	64.33	0.00	0.00	60.59	0.015	0.00	63.50	0.03	0.00	60.50	0.014	0.00	0.00	55.50	71.50	0.00	1.53	0.00	0.00	52.50	68.50	0.00	0.16
UIL.21.13850	Al Deyafa orang flavour (powder)	0.01	95.73	0.00	0.00	94.69	0.000	0.00	94.00	0.00		94.00	0.000	0.00	0.00	86.00	102.00	0.00	0.00			86.00	102.00	0.00	0.00
UIL.21.14134	Al Marai mango juice	0.38	15.40	0.10	0.00	11.81	0.000	0.00	12.50	0.00	0.00	12.20	0.000	0.00	0.00	10.00	15.00	0.00	0.00	0.00	0.00	9.76	14.64	0.00	0.00
UIL.21.14132	Al rabie fruit cocktail nectar	0.00	15.49	0.00	0.00	12.47	0.000		15.00	0.00		15.00	0.010			12.00	18.00	0.00	0.00			12.00	18.00	0.00	0.16
UIL.21.14133	al rawabi apple juice	0.26	10.35	0.10	0.02	9.15	0.000	0.11	10.85	0.11	0.02	10.37	0.040	0.00	2.11	8.68	13.02	0.00	1.61	0.00	0.82	8.30	12.44	0.00	0.19
UIL.21.14138	AL RAWABI RED GRAPE JUICE	0.25	15.18	0.10	0.00	13.96	0.000	0.23	15.40	0.12	0.00	15.40	0.004	0.00	2.23	12.32	18.48	0.00	1.62	0.00	0.00	12.32	18.48	0.00	0.15
UIL.21.14135	Almarai Graps and berries (farms select)	0.26	14.08	0.05	0.00	13.61	0.000	0.00	29.00	0.00	0.00	29.00	0.000	0.00	0.00	23.20	34.80	0.00	0.00	0.00	0.00	23.20	34.80	0.00	0.00
UIL.21.13822	ALMarai orange juice 100%300ml	2.40	9.59	0.00	0.00	9.39	0.000	1.00	29.00	0.00	0.00	29.00	0.006	0.00	3.00	23.20	34.80	0.00	0.00	0.00	0.00	23.20	34.80	0.00	0.16
UIL.21.13823	ALRAWABI ORANGE 1005JUICE	0.00	11.00	0.00	0.00	6.65	0.060		10.56	0.06	0.00	8.53				8.45	12.67	0.00	1.56	0.00	0.00	6.53	10.53		
UIL.21.13821	Asafwa natural Orange 200ml	0.00	12.94	0.00	0.00	10.25	0.165	0.90	13.40		0.10	160.00		0.00	2.90	10.72	16.08			0.00	0.90	152.00	168.00		
UIL.21.14139	A'SAFWAH MIXED FRUIT JUICE	0.08	14.07	0.10	0.00	11.56	0.000	0.10	12.30	0.10	0.00	12.00		0.00	2.10	9.84	14.76	0.00	1.60	0.00	0.00	9.60	14.40		
UIL.21.14143	Fruit drink (orange) , Rani	0.16	13.13	0.10	0.00	11.56	0.008	0.00	12.50	0.00	0.00	12.30	0.010	0.00	0.00	10.00	15.00	0.00	0.00	0.00	0.00	9.84	14.76	0.00	0.16

id_lab	Name of Product & Brand	Amounts per 100 g/ml from Laboratory analyses						Amounts per 100 g/ml from Labelling						Tolerances for the nutrition declaration on food products											
														Prot (g)		Carb (g)		TF (g)		SFA (g)		Sugars (g)		Na (g)	
		Prot[1] (g)	Carb[2] (g)	TF[3] (g)	SFA[4] (g)	Sugars (g)	Na5 (g)	Prot (g)	Carb (g)	TF (g)	SFA (g)	Sugars (g)	Na (g)	Low	High	Low	High	Low	High	Low	High	Low	High	Low	High
UIL.21.14142	Fruit Nectar (Lacnor)	0.09	13.20	0.10	0.00	12.73	0.002	0.10	13.95	0.10	0.10	13.95	0.002	0.00	2.10	11.16	16.74	0.00	1.60	0.00	0.90	11.16	16.74	0.00	0.15
UIL.21.13829	Mango Juice Mazoon 200ml	0.00	16.44	0.00	0.00	13.44	0.000	0.22	14.70	0.00				0.00	2.22	11.76	17.64	0.00	0.00						
UIL.21.13824	Mixed fruit juice (Mazoon)	0.00	13.24	0.00	0.00	11.77	0.000	0.22	14.70	0.00				0.00	2.22	11.76	17.64	0.00	0.00						
UIL.21.14136	Nada mango juice	0.26	12.78	0.01	0.00	10.50	0.000	0.40	24.80	0.00	0.00	22.00	0.000	0.00	2.40	19.84	29.76	0.00	0.00	0.00	0.00	17.60	26.40	0.00	0.00
UIL.21.14137	Nada orang juice	0.70	10.27	0.05	0.00	10.46	0.000	1.20	22.60	0.00	0.00	22.00	0.000	0.00	3.20	18.08	27.12	0.00	0.00	0.00	0.00	17.60	26.40	0.00	0.00
UIL.21.13827	Natural mango Asafwa 200ml	0.00	13.74	0.00	0.00	13.13	0.005	0.27	13.50	0.00	0.00	12.60	0.000	0.00	2.27	10.80	16.20	0.00	0.00	0.00	0.00	10.08	15.12	0.00	0.00
UIL.21.14141	Orange drink (Top fruit)	0.09	11.92	0.10	0.00	10.72	0.006	0.00	12.00	0.00	0.00	12.00	0.005	0.00	0.00	9.60	14.40	0.00	0.00	0.00	0.00	9.60	14.40	0.00	0.16
UIL.21.13826	Orange no add sugar (Top fruit)	0.00	10.45	0.00	0.00	9.14	0.009	0.00	10.00	0.00	0.00	9.00	0.008	0.00	0.00	8.00	12.00	0.00	0.00	0.00	0.00	7.00	11.00	0.00	0.16
UIL.21.14140	Sun top orange fruit drink	0.35	12.67	0.10	0.00	11.84	0.004	0.00	14.00	0.00	0.00	14.00	0.003	0.00	0.00	11.20	16.80	0.00	0.00	0.00	0.00	11.20	16.80	0.00	0.15
UIL.21.17402	Tang (orange) (powder)	0.04	96.92	0.00	0.00	96.92	0.181	0.10	23.00	0.00	0.00	23.00	0.043	0.00	2.10	18.40	27.60	0.00	0.00	0.00	0.00	18.40	27.60	0.00	0.19
UIL.21.13825	Topfruit fruit cocktail 200ml	0.00	11.38	0.00	0.00	9.28	0.023	0.00	11.00	0.00	0.00	11.00	0.020	0.00	0.00	8.80	13.20	0.00	0.00	0.00	0.00	8.80	13.20	0.00	0.17
UIL.21.13851	Vimto	0.00	12.58	0.00	0.00	12.39	0.000	0.10	12.50	0.00	0.00	12.50	0.002	0.00	2.10	10.00	15.00	0.00	0.00	0.00	0.00	10.00	15.00	0.00	0.15
UIL.21.14129	Evaporated milk (Rainbow)	5.94	10.57	8.85	6.25	9.54	0.105	6.90	10.00	8.50	5.80		0.100	4.90	8.90	8.00	12.00	7.00	10.00	4.64	6.96			0.00	0.25
UIL.21.13450	Galaxy smooth milk	3.85	92.31	0.38	0.08	73.08	0.000	2.30	20.00	12.00	7.00	20.00	0.029	0.30	4.30	16.00	24.00	9.60	14.40	5.60	8.40	16.00	24.00	0.00	0.18
UIL.21.13551	MAZOON FRESH MILK STRAWBERRY FLAVOUR	3.07	10.75	3.21	2.36	6.47	0.054	3.30	12.10	3.00	1.70	6.20	0.050	1.30	5.30	9.68	14.52	1.50	4.50	0.90	2.50	4.20	8.20	0.00	0.20
UIL.21.14570	Milk powder (Almudhish)	24.88	40.39	29.52	19.24	38.95	0.281	24.50	38.30	28.00	18.80	38.30	0.280	19.60	29.40	30.64	45.96	22.40	33.60	15.04	22.56	30.64	45.96	0.13	0.43
UIL.21.14569	Milk powder (Majan)	24.10	40.99	29.21	20.19	47.83	0.330	24.00	39.00	28.00	18.00	39.00	0.255	19.20	28.80	31.20	46.80	22.40	33.60	14.40	21.60	31.20	46.80	0.11	0.41
UIL.21.13553	Strawberry flavored milk	3.04	12.13	2.50	1.74	12.03	0.058	8.00	30.00	3.00	3.00	30.00	0.133	6.00	10.00	24.00	36.00	1.50	4.50	2.20	3.80	24.00	36.00	0.00	0.28
UIL.21.14171	Strawberry flavored milk (Lacnor)	2.82	10.96	3.26	1.94	9.84	0.070	2.70	11.80	3.00	1.80	11.50	0.066	0.70	4.70	9.44	14.16	1.50	4.50	1.00	2.60	9.20	13.80	0.00	0.22
UIL.21.14170	Strawberry milk (Nada)	2.18	11.68	3.21	2.27	9.67	0.049	3.10	11.50	3.00	2.10	11.50	0.043	1.10	5.10	9.20	13.80	1.50	4.50	1.30	2.90	9.20	13.80	0.00	0.19
UIL.21.13552	Strwaberry milk (Asafwah)	3.42	14.15	2.42	1.78	14.14	0.058	2.80	14.10	2.20	1.30	14.10	0.052	0.80	4.80	11.28	16.92	0.70	3.70	0.50	2.10	11.28	16.92	0.00	0.20
UIL.21.13554	strwberry fresh milk (Alrawabi)	4.05	11.45	3.28	2.32	11.77	0.151	2.84	12.43	3.11				0.84	4.84	9.94	14.92	1.61	4.61						
UIL.21.14130	Tea milk (Almudhish)	5.49	10.46	8.12	4.05	9.28	0.106	6.00	9.70	7.80	3.84	9.70	0.100	4.00	8.00	7.70	11.70	6.30	9.30	3.04	4.64	7.70	11.70	0.00	0.25
UIL.21.14173	Energy drink (Red bull)	0.01	11.58	0.01	0.00	10.15	0.045	0.00	10.70	0.00	0.00	10.70	0.041	0.00	0.00	8.56	12.84	0.00	0.00	0.00	0.00	8.56	12.84	0.00	0.19
UIL.21.13849	power horse energy	0.04	11.82	0.00	0.00	11.28	0.066	0.00	28.00	0.00	0.00	28.00	0.153	0.00	0.00	22.40	33.60	0.00	0.00	0.00	0.00	22.40	33.60	0.00	0.30
UIL.21.14174	Coca cola	0.01	10.95	0.01	0.00	9.78	0.015		10.60			10.60				8.48	12.72					8.48	12.72		
UIL.21.14172	Lipton Peach Ice tea	0.10	4.51	0.01	0.00	4.40	0.011	0.00	4.60	0.00	0.00	4.50	0.009	0.00	0.00	2.60	6.60	0.00	0.00	0.00	0.00	2.50	6.50	0.00	0.16
UIL.21.14177	Malt beverage (Barbican)	0.26	2.97	0.01	0.00	2.22	0.010	0.10	2.80	0.00	0.00	1.90	0.015	0.00	2.10	0.80	4.80	0.00	0.00	0.00	0.00	0.00	3.90	0.00	0.17
UIL.21.14176	Mountain Dew	0.01	12.31	0.01	0.00	11.55	0.016	0.00	12.00	0.00	0.00	12.00	0.015	0.00	0.00	9.60	14.40	0.00	0.00	0.00	0.00	9.60	14.40	0.00	0.17
UIL.21.14175	Pepsi	0.01	10.20	0.01	0.00	9.98	0.010	0.00	11.00	0.00	0.00	11.00	0.010	0.00	0.00	8.80	13.20	0.00	0.00	0.00	0.00	8.80	13.20	0.00	0.16
UIL.21.14178	Vitaene Extra (Pokka)	0.01	14.78	0.01	0.00	13.36	0.000	0.00	17.80	0.00		17.80	0.001	0.00	0.00	14.24	21.36	0.00	0.00			14.24	21.36	0.00	0.15

[1]: Protein. [2]: Carbohydrates. [3]: Total fat. [4]: Saturated fatty acids. [5]: Sugars. [6]: Sodium.
